# Systematic Engineering of Proteases in *Saccharopolyspora Spinosa* Reveals Synergistic Enhancement of Spinosad Biosynthesis via Substrate Flux Optimization

**DOI:** 10.1002/advs.202522638

**Published:** 2026-01-31

**Authors:** Duo Jin, Wangqiong Chen, Danlu Yang, Qing Liu, Baolong Bai, Zirui Dai, Xirong Liu, Zirong Zhu, Jie Rang, Liqiu Xia

**Affiliations:** ^1^ Hunan Provincial Key Laboratory of Microbial Molecular Biology College of Life Science Hunan Normal University Changsha China; ^2^ Hunan Norchem Pharmaceutical Co., Ltd Changsha Hunan China; ^3^ Key Laboratory for Matter Microstructure and Function of Hunan Province Key Laboratory of Low‐dimensional Quantum Structures and Quantum Control School of Physics and Electronics Hunan Normal University Changsha China

**Keywords:** metabolic regulation, proteases engineering, saccharopolyspora spinosa, spinosad, synergism

## Abstract

Natural product biosynthesis is tightly coupled to nitrogen allocation. However, scalable strategies to amplify pathway outputs remain limited. Here, atmospheric and room temperature plasma (ARTP) mutagenesis of *Saccharopolyspora spinosa* yielded a high‐performing strain, D184, which showed improved utilization of extracellular nitrogen sources together with enhanced spinosad production. To identify novel regulatory mechanisms linking nitrogen utilization with natural product biosynthetic efficiency, we established a combinatorial engineering workflow that integrates systematic protease genetic manipulation, multi‐omics analysis, and rational design. Using this workflow, the functional contributions of eight overexpressed high‐titer protease‐related targets were examined from a set of 21 protease‐related genes related to nitrogen utilization, which were categorized into three modules: membrane proteostasis (*htpX*), adenosine triphosphate (ATP)‐dependent proteolysis (*clpP*), and amino acid supply (*dap, pepP, metAP, alp*). Synergy modeling, coupled with cross‐module coordination, highlighted a representative triangular combination, *pepP‐clpP‐htpX*. Overexpressing this combination in D184 increased spinosad titer to 2318.87 mg/L in a nitrogen‐rich 50L bioreactor, corresponding to a 9.81 fold improvement over the wild type. Crucially, this study provided causal evidence that dynamic inter‐modular crosstalk governs nitrogen‐dependent metabolic flux in D184. These findings establish fundamental mechanistic insights into protease‐mediated regulation in *S. spinosa* and offer guidance for protease‐informed optimization of natural product biosynthesis.

## Graphical abstract

ARTP mutagenesis yielded *Saccharopolyspora spinosa* mutant D184 with improved extracellular nitrogen utilization. An integrated workflow of protease genetic manipulation, multi‐omics, and rational synergy design pinpointed a *pepP*‐*clpP*‐*htpX* synergistic triangular combination. Engineering this combination increased spinosad to 2318.87 mg/L in a 50L bioreactor, a 9.81 fold increase over the wild type, the first revealing cross‐module control of nitrogen‐dependent flux.

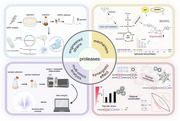

## 1 Introduction

Natural products, particularly secondary metabolites, are rich sources of bioactive compounds with diverse applications [1]. A prominent example is spinosad, a natural macrolide derived from *Saccharopolyspora spinosa*, that has been widely applied in agricultural pest management owing to its high efficacy, low toxicity, and environmental compatibility [2, 3, 4]. Although the biosynthetic pathway of spinosad has been largely elucidated, the intricate mechanisms of its metabolic regulation network and the dynamic balance between the secondary and primary metabolic pathways remain underexplored. In particular, the complex interactions among precursor supply, energy metabolism, and regulatory factors have not been fully elucidated. This knowledge gap has constrained efforts to achieve efficient spinosad biosynthesis through rational metabolic engineering. Therefore, characterizing the inherent regulatory logic of spinosad biosynthesis is crucial not only for elucidating the evolutionary patterns of secondary metabolic networks in actinomycetes but also for providing a theoretical foundation for the rational design and precise regulation of its biosynthetic pathways.

In wild‐type *S. spinosa*, inefficient primary metabolism and suboptimal carbon and nitrogen flux distribution restrict the availability of critical biosynthetic precursors [5, 6], including acetyl‐Coenzyme A (CoA), malonyl‐CoA, methylmalonyl‐CoA, and S‐adenosylmethionine [7, 8, 9]. Metabolic engineering strategies aimed at redistributing the carbon flux have proven to be effective approaches to alleviate precursor shortages in spinosad biosynthesis. Zhang et al. demonstrated that metabolic rewiring in the engineered hyperproducer strain *S. spinosa* S3‐3, through the coordinated upregulation of glycolytic enzymes, significantly enhanced glycolytic flux and acetyl‐CoA levels, thereby alleviating precursor bottlenecks [10]. Similar strategies have been validated in other engineered strains such as *S. spinosa::acuC* [11] and *S. spinosa‐∆pepM* [12], wherein targeted manipulation of glycolytic enzymes consistently improved spinosad titers. Moreover, optimizing triacylglycerol (TAG) metabolism [13, 14] and enhancing their β‐oxidation [15, 16] have been shown to improve fatty acid metabolism and increase the efficiency of spinosad synthesis. Furthermore, downregulation of citrate synthase expression to reroute carbon flux from the tricarboxylic Acid (TCA) cycle has been shown to increase pyruvate accumulation, thereby providing more precursors for acetyl‐CoA and spinosad synthesis [17]. In this regard, while carbon flux has received extensive research attention, nitrogen metabolism remains underexplored even though it represents a critical target for further optimization.

In addition to its essential role in supporting cellular growth and proliferation, nitrogen metabolism directly governs the availability of amino acids and other nitrogenous precursors crucial for natural product biosynthesis [18]. Notably, branched‐chain amino acids are key precursors for the synthesis of acyl‐CoA, which is a critical building block in spinosad biosynthesis [19]. This mechanistic connection explains why supplementation with exogenous amino acids consistently enhances spinosad biosynthesis [20]. Another study showed that the use of media enriched with complex organic nitrogen significantly boosted spinosad titers [7], thereby identifying nitrogen availability as a major limiting factor for biosynthetic efficiency. However, efficient utilization of organic nitrogen sources depends on both extracellular proteases for the initial hydrolysis of macromolecular proteins in the environment as well as the coordinated action of membrane‐bound and intracellular proteases for peptide transport, amino acid release, and subsequent metabolism [21, 22]. Although the precursor‐supplying role of amino acid metabolism has been established [23], a system‐level understanding of its regulatory mechanisms, particularly how protease‐mediated nitrogen metabolism is integrated with spinosad biosynthesis, remains a critical gap in the field. Emerging evidence [24, 25, 26] has implicated specific proteases as key modulators of polyketide biosynthesis. For example, overexpression of the subtilisin‐like serine peptidase encoded by locus APASM_4178 in *S. albus* BK 3–25 has been shown to increase the salinomycin titer by 33.8 % [27], whereas heterologous expression of the *S. griseus* chymotrypsin‐like serine protease SprD enhanced the titer of actinorhodin in *S. lividans* [28]. These findings suggest that proteases are promising targets for enhancing polyketide biosynthesis, complementing traditional strategies such as carbon flux optimization and polyketide synthase (PKS) engineering. However, the existing research has primarily focused on individual proteases, leaving the broader regulatory mechanisms, particularly their interplay with amino acid metabolism and secondary metabolic networks, poorly understood. Moreover, a systematic investigation of the synergistic effects of protease families within the metabolic network and their influence on spinosad biosynthesis remains an unmet need.

This study established a proteolytic regulatory network that optimizes spinosad biosynthesis in *S. spinosa* through combinatorial integration of atmospheric and room temperature plasma (ARTP) mutagenesis, systematic protease genetic manipulation, multi‐omics analysis, and rational design. Through functional screening of 21 protease‐associated genes and subsequent overexpression of key strains, we identified a closed‐loop flux system that enhances metabolic flux, precursor availability, and spinosad biosynthesis. Targeted manipulation of nitrogen metabolism pathways revealed synergistic interactions essential for enhanced biosynthesis. These synergistic interactions coordinated three interdependent modules: membrane proteostasis, adenosine triphosphate (ATP)‐dependent proteolysis, and amino acid supply. To the best of our knowledge, this study reports the first protease‐synergy optimization workflow in *S. spinosa* that systematically coordinates proteolysis, precursor flux, and pathway regulation, which inform flux reprogramming efforts to enhance spinosad biosynthesis.

## 2 Materials and Methods

### 2.1 Strains and Growth Conditions

All strains used in this study are listed in Table S1. Additional information is provided in Table S1.

### 2.2 ARTP Mutagenesis and Strain Screening

Scrape the white spores of *S. spinosa* from the TSB solid medium and wash them three times in physiological saline at 4°C for 30 s at 9000 rpm. A 15 µL aliquot of the spore suspension was evenly spread onto the metal slide of an atmospheric and room‐temperature plasma (ARTP) mutagenesis system. Perform mutagenesis with the instrument set to the following settings: 10.0 SLM pure helium flow rate, 100 W power, and a treatment distance of 2.0 mm. The mutagenesis durations were 0 s (control), 25, 50, 75, 90, 100, 120, and 140 s. After treatment, the spore suspension was recovered in a shaking incubator at 30°C and 850 rpm for 24 h. Spore suspensions from each irradiation time point underwent serial dilutions and were spread onto TSB solid medium. For each exposure time, serial‐dilution plating was performed in triplicate, and each dilution was plated in three technical replicates. The lethality rate was calculated as Lethality rate (%) = (A—B) / A × 100 %, where A represents the colony count in the control group, and B represents the colony count in the ARTP‐treated group. Spore suspensions exhibiting a mutagenic lethality rate of approximately 90 % were selected for screening. These suspensions underwent gradient dilution and TSB plate plating to observe mycelial development. Well‐developed mutant strains were selected for fermentation. Finally, the spinosad titer was determined via Ultra‐high performance liquid chromatography (UHPLC; Agilent 1290) analysis of the fermentation broth collected on day 12 after four rounds of ARTP mutagenesis and high‐throughput screening of over 2000 transformants. A mutant strain with the highest spinosad titer was selected and designated as D184.

High‐throughput screening was performed in the single‐cell microliter‐droplet culture omics system (Wuxi Yuanqing Tianmu Biological Technology Co., Ltd., Wuxi, China). Following ARTP mutagenesis at an approximate lethality of 90 %, the *S. spinosa* culture was diluted to 50–100 CFU/mL and dispensed into sampling vials. Droplets were generated and incubated at 28°C for 96 h, and then sorted by optical density at 600 nm (OD_600_); the instrument threshold was set to OD_600_ >5, and droplets with OD_600_ >10 were selected for streak purification and subsequent scale‐up cultivation. These colonies were then expanded, fermented, and subjected to UHPLC analysis of the fermentation broths to screen and identify high‐titer mutant strains.

### 2.3 Construction of Recombinant Plasmids and Engineered Strains

Construction of Recombinant Plasmids. The genome of *S. spinosa* was used as the template for constructing single‐gene overexpression vectors. PCR products and the expression vector pOJ260–P*KasO* ^*^ (pOJ260 bearing the constitutive P*KasO* ^*^ promoter) were double‐digested with *Xba* I and *Bam*H I, and ligated using T4 DNA ligase to generate single‐gene overexpression plasmids. For dual‐gene co‐expression, each coding sequence was cloned downstream of an independent P*kasO* ^*^ promoter on the same pOJ260 vector: the first insert was introduced via   *Xba* I and *Bam*H I and the second via *Eco*R I/*Eco*R V, yielding constructs of the form pOJ260–P*kasO* ^*^–*X1*–P*kasO* ^*^‐*X2*. For the three‐gene overexpression system, the *X1* and *X2* genes were first amplified via fusion PCR to generate the *X1*‐*X2* fusion fragment. This fusion fragment was then digested with the restriction endonucleases *Xba* I and *Bam*H I, and subsequently inserted into the *Xba* I/*BamH* I restriction sites of the pOJ260‐P*kasO* ^*^‐P*kasO* ^*^‐*X3* vector. The recombinant expression vector pOJ260‐*X1*‐*X2*‐*X3* was successfully constructed. All recombinant plasmids were validated via restriction enzyme digestion or PCR of the corresponding target genes. The amplified sgRNA cassettes and the dCas9 carrier plasmid pSET‐dCas9 were double‐digested (*Xba* I/*Eco*R I) and ligated with T4 DNA ligase. Ligation mixtures were transformed into *E. coli* TOP10 by heat shock, and transformants were screened by colony PCR. CRISPR interference (CRISPRi) knockdown vectors were constructed using sgRNA primers designed at https://portals.broadinstitute.org/gppx/crispick/public. These primers incorporated *Xba* I and *EcoR* I restriction sites with protective bases at the respective upstream and downstream primer ends. The amplified sgRNA cassettes and the dCas9 carrier plasmid pSET‐dCas9 29 were double‐digested (*Xba* I/*EcoR* I) and ligated with T4 DNA ligase. Ligation mixtures were transformed into *E. coli* TOP10 by heat shock, and transformants were screened by colony PCR. All CRISPRi constructs were confirmed by Sanger sequencing before use. Restriction endonucleases and T4 DNA ligase were from Takara Bio (Shiga, Japan), and high‐fidelity DNA polymerase was used for all cloning PCRs. Detailed primer sequences are provided in Table S2.

Construction of engineered strains. Recombinant plasmids were introduced into *S. spinosa* by intergeneric conjugation from the donor strain *E. coli* ET12567/pUZ8002. Following conjugation, exconjugants were selected on apramycin‐containing plates, and the recombinant vectors were integrated into the *S. spinosa* chromosome. Integration and expression were confirmed by PCR targeting the apramycin‐resistance marker (*apr*) and by reverse transcription‐quantitative polymerase chain reaction (RT‐qPCR) of the engineered target genes.

### 2.4 Quantification of Spinosad and Bioassay of Insecticidal Activity

Spinosad titer determination by UHPLC. Fermentation broths (day 12) were extracted three fold with methanol. Combined extracts were clarified by centrifugation and passed through a 0.45 µm organic‐phase syringe filter. Spinosad was quantified on an ultra‐high‐performance liquid chromatograph (UHPLC; Agilent 1290) equipped with a C18 column (aq12s05‐1546, YMC, Japan). The mobile phase consisted of methanol (42 %, v/v), acetonitrile (42 %, v/v), and 16 % (v/v) ammonium acetate solution (20 g/L); isocratic elution was applied at 1.0 mL/min for 25 min. Detection was at 250 nm. The injection volume was 20 µL.

#### 2.4.1 Insecticidal Activity Against *Helicoverpa Armigera*

*H. armigera* larvae were reared on an artificial diet at 28°C and used at the second‐3rd instar. Fermentation broths harvested on day 12 were subjected to repeated freeze‐thaw cycles to disrupt the cells, then diluted 5 fold with sterile saline and thoroughly mixed into the artificial diet. For each test, 0.25 cm^3^ diet portions (1 cm × 1 cm × 0.25 cm) were dispensed into 24‐well cell‐culture plates, one larva per well. Sterile saline served as the blank control. Each condition included three biologically independent replicates. Larval survival was recorded every 12 h.

### 2.5 Morphological and Physiological Phenotyping

The morphological and physiological characteristics of the cultures were analyzed using phase‐contrast microscopy and scanning electron microscopy (SEM), while biomass was determined by dry cell weight (DCW). For glucose, total extracellular proteases activity, and extracellular protein quantification, the DNS assay, Total Protease Activity Assay Kit (Jiangsu Aidisheng Biological Technology Co., Ltd.), and Micro BCA Protein Assay Kit (Sangon Biotech, China) were used, respectively, with all measurements performed in triplicate. Detailed methods can be found in the Supporting Information.

### 2.6 Determination of Intracellular Short‐Chain Acyl‐Coenzyme As (acyl‐CoAs), Adenosine Triphosphate (ATP), and Nicotinamide Adenine Dinucleotide Phosphate (NADPH)

Biomass samples were collected on fermentation days 4 and 8, rapidly quenched, and cryomilled in liquid nitrogen to release intracellular contents. Intracellular short‐chain acyl‐CoAs (acetyl‐CoA, malonyl‐CoA, and methylmalonyl‐CoA), ATP, and NADPH were quantified using microorganism ELISA assay kits (Jiangsu JINMEI Biotechnology Co., Ltd., Jiangsu, China) following the manufacturer's instructions. Absorbance was recorded on a microplate reader, and concentrations were calculated from kit‐provided standard curves. Each measurement was performed with three biologically independent replicates.

### 2.7 Total RNA Extraction and RT‐qPCR Analysis

Total RNA was isolated from biomass samples of the indicated strains using TotalRNA Extractor reagent (following the manufacturer's protocol). RNA quantity and purity were assessed with a NanoDrop 2000 spectrophotometer (Thermo Scientific). Genomic DNA was removed, and first‐strand cDNA was synthesized from total RNA using a fast reverse‐transcription kit with gDNA Eraser (Sangon Biotech, Shanghai, China) according to the supplier's instructions. Transcript abundances of target genes were quantified by RT‐qPCR as described previously [30]. The 16S rRNA gene served as the internal reference for normalization. Relative expression levels were calculated by the 2^−ΔΔCt^ method based on Ct values. Log_2_ fold changes were reported as −ΔΔCt. Three biologically independent samples were analyzed for each condition. Primer sequences are listed in Table S2.

### 2.8 Genome Resequencing, DIA‐Based Quantitative Proteomics and Metabolomics Analysis

Differential analysis of DIA (Data‐Independent Acquisition)‐based quantitative proteomics and metabolomics data was performed using a two‐tailed Student's *t*‐test. Differential proteins were identified with thresholds of FC ≥ 1.5 or FC ≤ 0.6667 and *p*< 0.05, while metabolites were considered significantly different with a threshold of *p*< 0.05. FC ≥ 1.5 were considered upregulated, FC ≤ 0.6667 were considered downregulated. Genome resequencing, DIA‐based quantitative proteomics, and metabolomics methods are described in detail in the Supporting Information.

### 2.9 Synergy Analysis

To quantify whether pairwise co‐expression of protease genes produced synergistic gains in spinosad titer, we applied an additivity‐based interaction metric commonly used in drug‐combination research [31, 32]. For each pair (A, B), a synergy score *s* was calculated as:

$$s=\frac{YAB\;-\;Y0}{\left(YA\;-\;Y0\right)+\left(YB\;-\;Y0\right)}$$

Whereas *Y0* is the mean titer of the parental strain, *YA* and *YB* are the mean titers of the single‐gene overexpression strains, and *YAB* is the mean titer of the corresponding co‐expression strain. Fermentation titer is a continuous quantitative outcome; therefore, A ± 0.15 tolerance band was applied to define the additivity region [33]. So scores were interpreted as follows: *s*< 0.85, antagonism; 0.85 ≤ *s*< 1.15, additivity; *s* ≥ 1.15, synergy.

### 2.10 Statistical Analysis

All data in this study were stated as means ± standard deviation (SD)and analyzed by one‐way ANOVA (>2 groups) followed by Dunnett's/Tukey's post‐hoc test, or two‐tailed *t*‐test, with (^*^) *p*< 0.05, (^**^) *p*< 0.01, (^***^) *p*< 0.001, and ns indicating no significant difference. n = 3 independent biological replicates per panel unless otherwise indicated in the figure legends. Statistical analyses were carried out using GraphPad Prism and SPSS.

Graphical Abstract Overview of the protease genetic manipulation, multi‐omics analysis, and rational design strategy. The process begins with ARTP mutagenesis, followed by protease screening and the construction of engineered strains, represented by the blue background. The yellow background indicates systematic assessments of precursor supply, cellular energy, and redox homeostasis, and transcription of the spinosad biosynthetic gene cluster, thereby substantiating metabolic support for spinosad biosynthesis. Multi‐omics profiling of D184 and proteomic analyses of D184‐*dap* and D184‐*pepP*‐*clpP*‐*htpX* are depicted with a purple background. The synergy strategy is highlighted with a red background, illustrating how these components work together to enhance spinosad biosynthesis. Single‐gene overexpression identified high‐titer protease targets; pairwise co‐overexpression titers were analyzed using an additivity‐based interaction metric to construct a synergy map and identify synergistic triangular combinations; three‐gene co‐expression then validated the predicted synergy and titer improvement.

## 3 Results

### 3.1 ARTP‐Derived Strain D184 Boosts Nitrogen Utilization and Spinosad Titers

**FIGURE 1:**
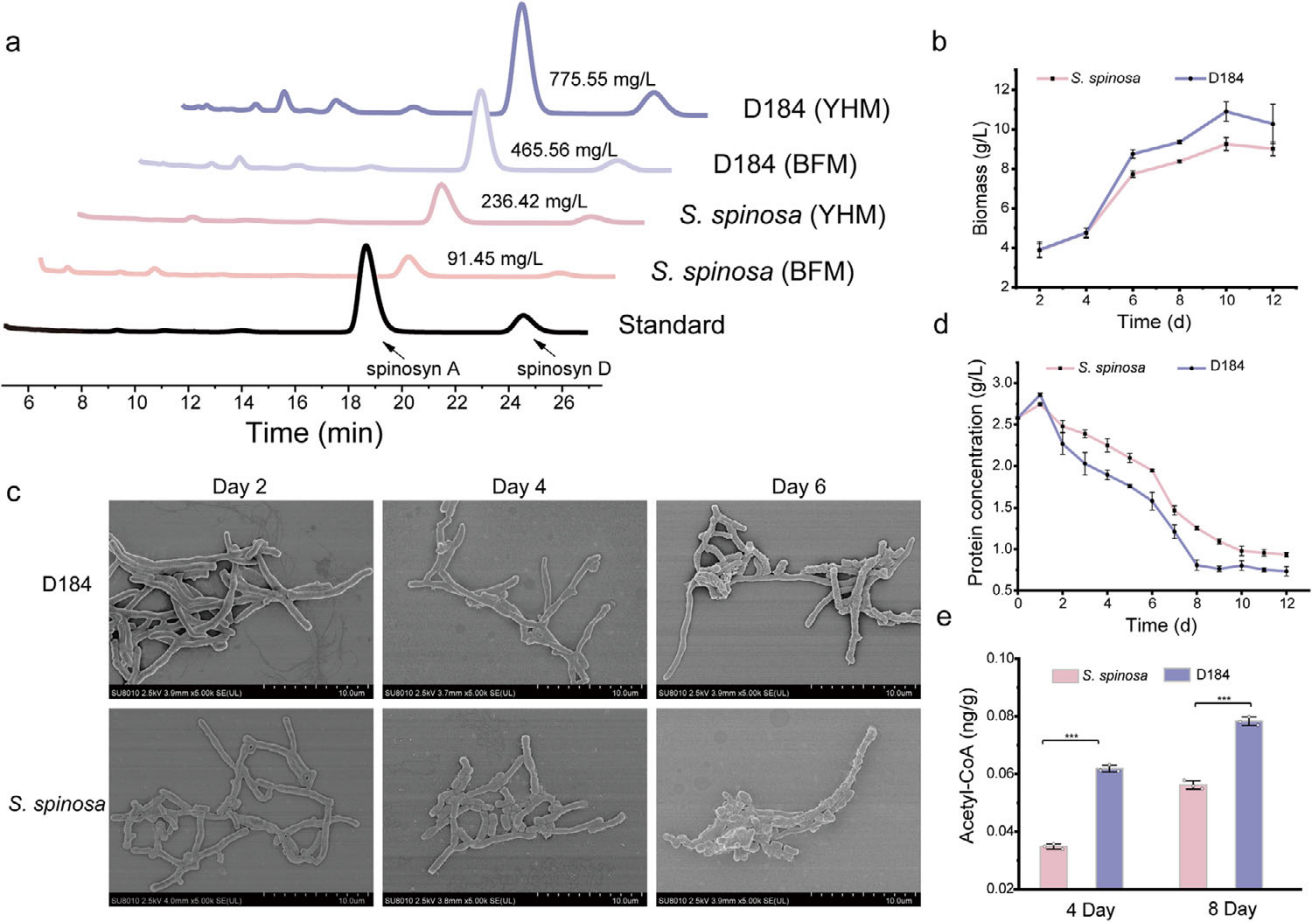
Generation of ARTP‐derived mutant D184 and characterization of enhanced spinosad titer. (a) UHPLC analysis of fermentation broths from the ARTP‐derived mutant D184 and wild‐type *S. spinosa* after 12 days of fermentation in BFM and improved YHM media. (b) Scanning electron microscopy images of *S. spinosa* and D184 on days 2, 4, and 6 of fermentation, with a magnification of 5.00K. (c) Biomass determination of *S. spinosa* and D184. (d) Measurement of extracellular protein content in *S. spinosa* and D184. (e) Acetyl‐CoA precursor determination in *S. spinosa* and D184 on days 4 and 8. Statistical significance determined using the *t*‐test (n = 3). ^*^ *p*< 0.05, ^**^ *p*< 0.01, ^***^ *p*< 0.001.

To systematically elucidate the genetic basis of enhanced spinosad biosynthesis, we employed a comprehensive mutagenesis and screening strategy using the wild‐type *S. spinosa* (CCTCC M206084). Through ARTP mutagenesis and high‐throughput screening, we identified the mutant D184, which showed significant improvements in spinosad synthesis (Figure S1). Comparative batch fermentations revealed that D184 achieved a spinosad titer of 467.56 ± 25.79 mg/L in basic fermentation medium (BFM), representing a 5.11 fold increase over the wild‐type strain (91.45 ± 28.87 mg/L). This titer advantage was maintained in the protein‐rich nitrogen source nutrient‐rich medium (YHM), where the spinosad titer achieved by D184 (775.55 ± 21.58 mg/L) was 3.28 fold higher than that of wild‐type *S. spinosa* (236.42 ± 20.05 mg/L) (Figure 1a). These results clearly illustrate that D184 exhibits robust and superior biosynthetic capacity in both YHM and BFM, indicating that nitrogen availability plays a critical role in regulating spinosad biosynthesis.

To elucidate the molecular and physiological mechanisms underlying the enhanced spinosad titer in D184 in comparison with wild‐type *S. spinosa*, we conducted a systematic comparative analysis of key metabolic and morphological parameters in YHM, including biomass accumulation, mycelial morphology, intracellular acetyl‐CoA levels, and extracellular protein consumption. Our results demonstrated that D184 exhibited a significantly higher growth rate, reaching a maximum biomass (10.90 g/L), surpassing that of wild‐type *S. spinosa* (9.25 g/L), and its hyphae were longer and more robust at 2 and 4 days (Figure 1b,c). Additionally, D184 exhibited higher extracellular protease activity and a faster rate of extracellular protein consumption, which correlated with its growth rate (Figure 1d; Figure S2), and acetyl‐CoA abundance in D184 at days 4 and 8 was higher than that in wild‐type *S. spinosa* (Figure 1e). Collectively, these findings suggested that D184 undergoes metabolic rewiring, optimizing both protein utilization and acetyl‐CoA synthesis under nitrogen‐rich conditions. The improved hyphal morphology further supports enhanced nutrient absorption, whereas elevated acetyl‐CoA levels and increased biomass accumulation directly contribute to a higher spinosad titer.

**FIGURE 2:**
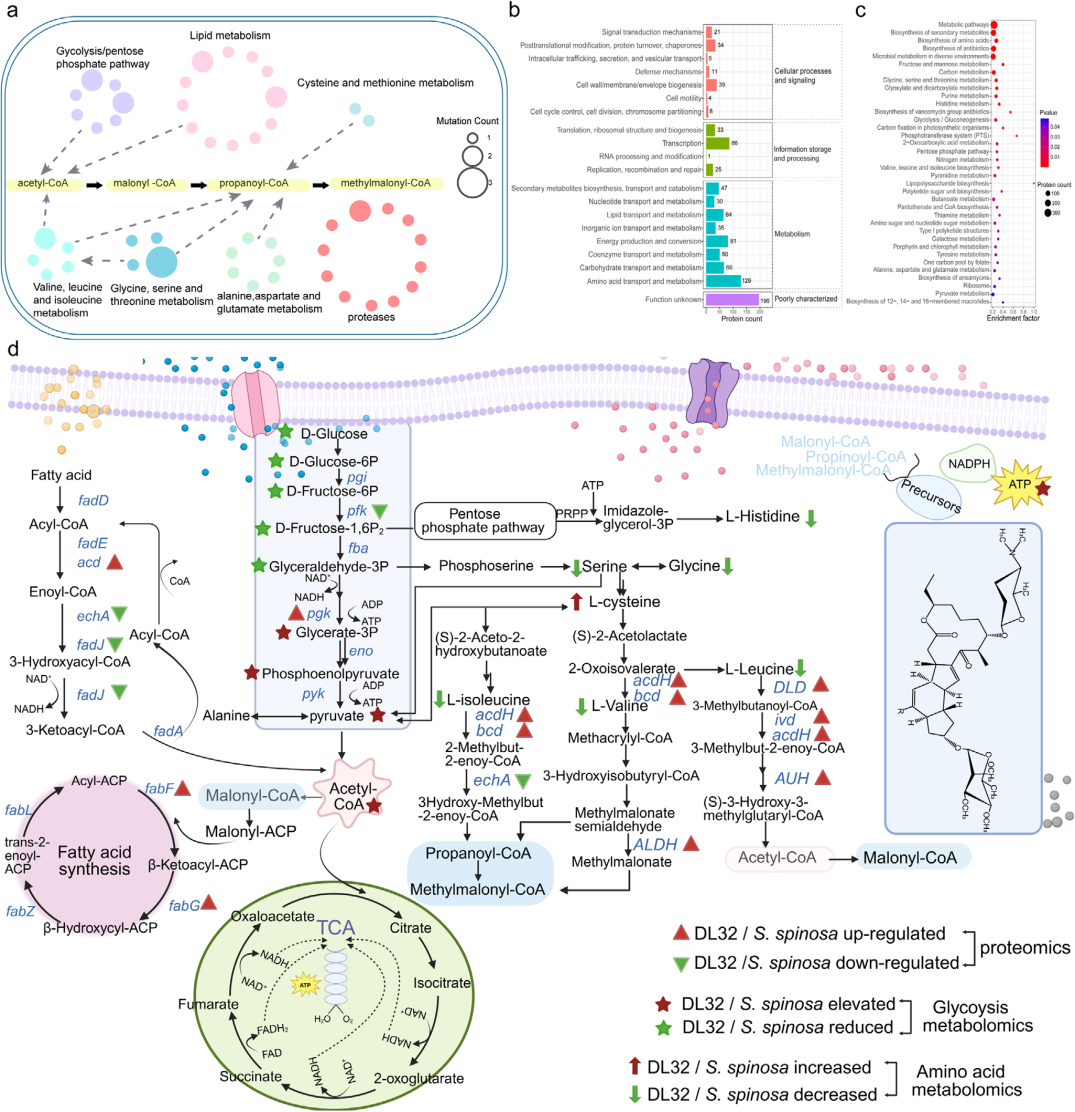
Comprehensive analysis of the genomic, proteomic, and metabolic differences between the mutant strain D184 and wild‐type *S. spinosa*. (a) Genomic comparison of D184 and wild‐type *S. spinosa*, highlighting mutation sites related to spinosad biosynthesis pathways based on SNPs analysis. Circles represent individual genes, with circle size proportional to the number of mutations identified in each gene. (b) EggNOG statistical analysis of D184 and wild‐type *S. spinosa* in YHM after 4 days of fermentation. (c) KEGG functional enrichment analysis of D184 and wild‐type *S. spinosa* in YHM after 4 days of fermentation. (d) Metabolic network diagram of spinosad biosynthesis based on quantitative proteomics and metabolomics data for D184 and *S. spinosa*. In the proteomics analysis, red triangles indicate upregulated proteins in D184 compared to *S. spinosa*, while green triangles represent downregulated proteins. In the glucose metabolism analysis, red pentagons indicate elevated levels of intermediate metabolites in D184 compared to *S. spinosa*, and green pentagons indicate reduced levels. In the amino acid metabolism analysis, red upward arrows represent increased amino acid levels in D184, and green downward arrows indicate decreased amino acid levels in D184. Differential proteins and metabolites were defined as upregulated (FC ≥ 1.5, *p*< 0.05) or downregulated (FC ≤ 0.67, *p*< 0.05).

We compared the genome of D184 with that of the wild‐type strain. Detailed alignment of the coding sequences (CDSs) revealed 903 single‐nucleotide polymorphisms (SNPs) (Supporting Information; Figure S3). Kyoto Encyclopedia of Genes and Genomes (KEGG) pathway analysis linked these mutations primarily to amino acid metabolism, protease activity, lipid metabolism, and glycolysis/pentose phosphate pathways (Figure 2a). Notably, these mutations converged in enzymes that governed three key nodes: proteolytic efficiency (enhancing extracellular protein hydrolysis and nitrogen utilization), glycolytic flux, and acyl‐CoA metabolism (optimizing malonyl/methylmalonyl‐CoA pools for polyketide assembly). To functionally validate these genomic insights, we conducted comparative proteomics and targeted metabolomics analyses using day 4 samples. In total, 3436 proteins were identified on D184, of which 1082 were differentially abundant proteins (Supporting Information). The eggNOG annotation indicated that “amino acid transport and metabolism” accounted for the largest number of differentially abundant proteins (n = 129) (Figure 2b). Pathway analyses revealed coordinated upregulation across KEGG categories associated with proteolysis turnover and amino acid metabolism and transport (Figure 2c,d). In the metabolism of branched‐chain amino acids (BCAAs), the abundance of most key proteins was upregulated, such as isovaleryl‐CoA dehydrogenase (IVD), branched‐chain α‐keto acid dehydrogenase (Bcd), and aldehyde dehydrogenase (ALDH). Moreover, the amino acid metabolomics data showed a significant decrease in the abundance of most amino acids, including glycine, cysteine, leucine, isoleucine, valine, and serine, suggesting that these amino acids were likely consumed in substantial amounts or redirected into acyl‐CoA or other precursor molecules to support spinosad biosynthesis (Figure 2d; Table S3). In contrast, the abundance of fatty acid degradation proteins was not significantly upregulated, and the key glycolytic enzyme phosphofructokinase (PFK) was notably downregulated. (Figure 2d; Figure S4). Glucose metabolomics further revealed a substantial reduction in upstream glycolytic metabolites (D‐glucose, D‐glucose‐6P, D‐fructose‐6P, D‐fructose‐1,6P, and glyceraldehyde‐3P) in D184 in comparison with the wild‐type strain, whereas downstream metabolites (glycerate‐3P and pyruvate) were elevated (Table S4; Figure 2d). These patterns suggest that carbon metabolic flux was still present in D184, but its distribution may have shifted to the high‐titer state. Additionally, elevated pyruvate likely reflects convergent inputs from central carbon metabolism and amino acid turnover, supported by the depletion of serine and glycine in metabolomics data (Figure 2d). Taken together, these results indicate coordinated carbon‐nitrogen metabolic reprogramming in D184, with the most consistent and pronounced changes converging in protease‐driven nitrogen utilization and amino acid turnover.

### 3.2 Targeted Protease Upregulation Boosts Spinosad Production

Given the dual role of proteases in hydrolyzing cellular and extracellular proteins into bioavailable amino acids while maintaining proteome homeostasis, we hypothesized that precisely engineering D184's protease network could synergistically enhance free amino acid pools, channeling precursors toward polyketide biosynthesis and metabolic resource allocation and thereby more effectively supplying the polyketide synthase (PKS) machinery. To test this hypothesis, we used quantitative proteomics data to identify 21 significantly up‐regulated proteases (Supporting Information), and RT‐qPCR confirmed transcriptional upregulation of the corresponding genes (Figure S5). On the basis of their annotated cellular roles, these candidate proteases spanned three functional modules: subcellular localization, substrate scope, and ATP dependence. The membrane proteostasis module (HtpX and YbbJ) maintains membrane protein homeostasis, supports stable substrate uptake, and mitigates envelope stress. The ATP‐dependent proteolysis module (ClpP, ClpX, ClpC, ClpA/B, and Lon) represents the ATP‐driven protease machinery responsible for regulating protein turnover and proteome allocation through ATP‐coupled substrate processing, thereby potentially shaping the pathway flux. The amino acid supply module encompasses enzymes primarily involved in nitrogen recycling and precursor release, including exopeptidases (MetAP, PepP, PepN, Dpp, VanY, Mbp, and Dap) and processing endoproteases (Alp, Ctr, SerA, Ppl, ZpeA, YtcJ, and Abh), which facilitate efficient protein degradation and precursor release (Table S5). The MEROPS family assignments, catalytic classes, and subcellular localizations of each target are summarized in Table S6.

**FIGURE 3:**
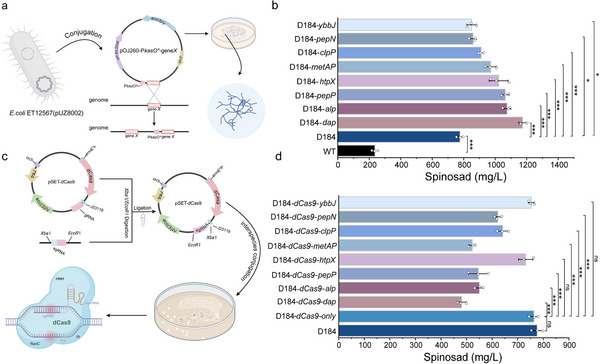
Construction and Titer Analysis of Protease‐Overexpressing and CRISPRi‐Knockdown Engineered Strains for Enhanced Spinosad Biosynthesis. (a) Schematic representation of the construction principle of protease‐overexpressing engineered strains. (b) Spinosad titer of eight protease‐overexpressing engineered strains showing enhanced biosynthesis. (c) Schematic representation of the construction of engineered strains for gene knockdown using CRISPR interference (CRISPRi). (d) Titer of spinosad from the CRISPRi‐knockdown strains, illustrating the impact of gene suppression on the synthesis levels. Mean concentrations with error bars showing SD are plotted (three biologically independent samples). Multiple comparison significance was tested to ^*^ *p*< 0.05, ^**^ *p*< 0.01, ^***^ *p*< 0.001 by one‐way ANOVA followed by Dunnett's post‐hoc test.

We placed each of the 21 candidate genes under the control of the strong constitutive promoter P*kasO* ^*^ and introduced the constructs into the parental strain D184 by intergeneric conjugation. RT‐qPCR verification of target upregulation was performed to establish an isogenic overexpression (OE) strain library (Figures S6 and S7). As shown in Figure 3a,b and Figure S8, spinosad titers did not increase in any of the engineered strains, and the initial shake‐flask screening yielded final titers ranging from a 44.21 % decrease to a 51.27 % increase relative to D184. Overexpression of *dap*, *pepP*, *pepN*, *alp*, *metAP*, *clpP*, *htpX*, and *ybbJ* reproducibly increased spinosad titers; the spinosad titer in D184‐*dap* reached 1173.15 mg/L, a 1.51 fold improvement over the parental D184. Bioactivity assessment against *H. armigera* larvae demonstrated accelerated mortality kinetics for all high‐performing strains (Figure S9). Notably, D184‐*dap* exhibited the most potent insecticidal activity, showing significantly reduced median lethal time (LT_50_) that correlated with its superior titer (Table S7). To further validate these findings, CRISPR interference (CRISPRi) was employed to knockdown eight beneficial protease genes in wild‐type D184 (Figure 3c; Figures S10 and S11). RT‐qPCR confirmed effective repression of the target genes throughout fermentation (days 2, 4, and 8; Figure S11b). All eight CRISPRi strains exhibited overall reductions in final spinosad titers (Figure 3d). Knockdown of the amino acid supply module (*dap*, *pepP*, *pepN*, *metAP*, and *alp*) produced pronounced reductions (*p*< 0.05), whereas repression of the ATP‐dependent proteolysis module (*clpP*) and membrane proteostasis module (*htpX* and *ybbJ*) resulted in decreased titers or no increase (*p*< 0.05, ns). These results demonstrated that protease genetic manipulation is an effective strategy for enhancing spinosad biosynthesis, particularly through amino acid‐supplying proteases.

### 3.3 Protease‐Mediated Metabolic Remodeling Enhances Spinosad Biosynthesis

**FIGURE 4:**
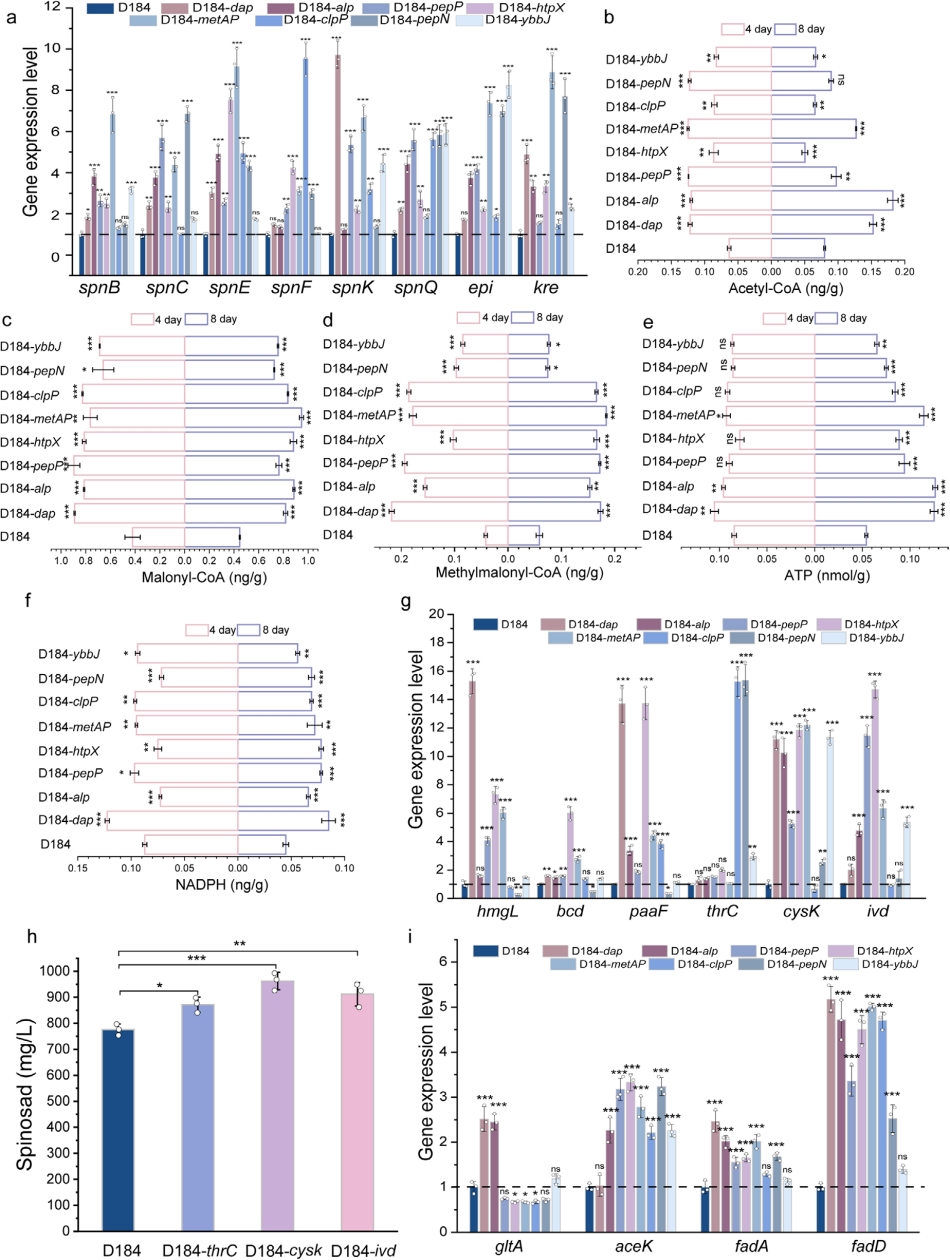
Protease overexpression enhances spinosad biosynthesis via *spn* gene cluster activation and precursor supply augmentation. (a) Transcriptional analysis of the *spn* gene cluster. (b–f) Levels of acetyl‐CoA, malonyl‐CoA, methylmalonyl‐CoA, ATP, and NADPH were measured on days 4 and 8 of culture. (g) RT‐qPCR analysis of six genes involved in amino acid metabolism, including *hgml*, *bcd*, *paaF*, *thrc*, *cysK*, and *ivd*, in eight protease‐overexpressing engineered strains. (h) Titer of spinosad in engineered strains overexpressing *thrC*, *cysK*, *ivd*. Specific genes related to the metabolism of branched‐chain amino acids, cysteine, and threonine were chosen for overexpression in D184. (i) Transcriptional regulation of the tricarboxylic acid cycle (TCA) and fatty acid metabolism pathways. Multiple comparison significance was tested to ^*^ *p*< 0.05, ^**^ *p*< 0.01, ^***^ *p*< 0.001 by one‐way ANOVA followed by Dunnett's post‐hoc test.

We conducted comprehensive phenotypic and physiological analyses of eight high‐performing protease‐overexpressing strains to elucidate the mechanistic basis of spinosad accumulation (Supporting Information and Figures S12–S16), with a particular focus on the expression of the *spn* biosynthetic gene cluster and its associated metabolic characteristics. We first quantified the transcripts of polyketide backbone genes (*spnB*, *spnC*, and *spnE*), terminal modification genes (*spnF* and *spnK*), and glycosylation‐related genes (*spnQ*, *epi*, and *kre*) to systematically evaluate the changes in spinosad biosynthetic capacity across the high‐titer engineered strains. As shown in Figure 4a, although the expression of a few genes did not reach statistical significance in certain strains, the overall transcriptional landscape showed consistent upregulation of the most critical *spn* pathway genes across the eight engineered strains, reflecting pathway‐wide, coordinated activation under the engineering interventions. Based on these findings, we hypothesized that metabolic reinforcement of cofactor supply (ATP, NADPH) and precursor availability (acyl‐CoAs) contributes to enhanced spinosad biosynthesis. Time‐course analysis revealed dynamic metabolic changes during fermentation. Malonyl‐CoA and methylmalonyl‐CoA levels were significantly elevated (*p*< 0.05) in all high‐producing strains on days 4 and 8, indicating an enhanced supply of polyketide chain‐elongation units (Figure 4b–d). While the ATP differences were not significant on day 4, most strains showed marked increments in both ATP and NADPH levels by day 8 (*p*< 0.05), suggesting that energy metabolism became increasingly important during the biosynthesis phase (Figure 4e,f). These coordinated metabolic changes collectively supported the high‐titer phenotype observed in the engineered strains.

Quantitative analysis revealed that all high‐production strains exhibited enhanced extracellular total protease activity and protein hydrolysis in comparison with D184 (Figures S17 and S18). Elevated extracellular protease activity was observed across eight high‐titer protease‐overexpression strains, consistent with the extracellular protein concentration, suggesting broader remodeling of protease‐associated functions and envelope‐secretion capacity. Notably, strains D184‐*dap*, D184‐*htpX*, D184‐*metAP*, and D184‐*ybbJ* demonstrated the most pronounced proteolytic activity. This enhanced proteolysis was correlated with distinct transcriptional reprogramming of amino acid metabolic pathways. The strains exhibiting the strongest hydrolytic activity during the early stages (D184‐*dap* and D184‐*metAP*) showed coordinated upregulation of both BCAA catabolic genes (*hmgL*, *bcdH*, *ivd*, and *paaF*) and biosynthetic genes (*cysK*), indicating comprehensive metabolic activation (Figure 4g). Notably, D184‐*dap* showed exceptional induction of BCAA degradation genes (15.3–1.6 fold), while D184‐*htpX* and D184‐*ybbJ* preferentially upregulated *cysK* (11.8–11.3 fold). Additionally, D184‐*clpP* and D184‐*pepN* preferentially upregulated *thrC* (15.26–15.35 fold), reflecting strain‐specific optimization of precursor supply. Validation through single‐gene overexpression (*cysK*, *ivd*, and *thrC*) confirmed this metabolic strategy, with all engineered strains achieving significantly higher titers than D184 (Figure 4h; Figure S19), demonstrating the effectiveness of targeted pathway reinforcement. In addition, increased protease expression not only enhanced amino acid turnover but also systematically regulated central carbon metabolism, including the TCA cycle and fatty acid metabolism pathways (Figure 4i), thereby optimizing the metabolic flux toward spinosad biosynthesis.

### 3.4 Proteomic Analysis Reveals Protease‐Centric Metabolic Rewiring

**FIGURE 5:**
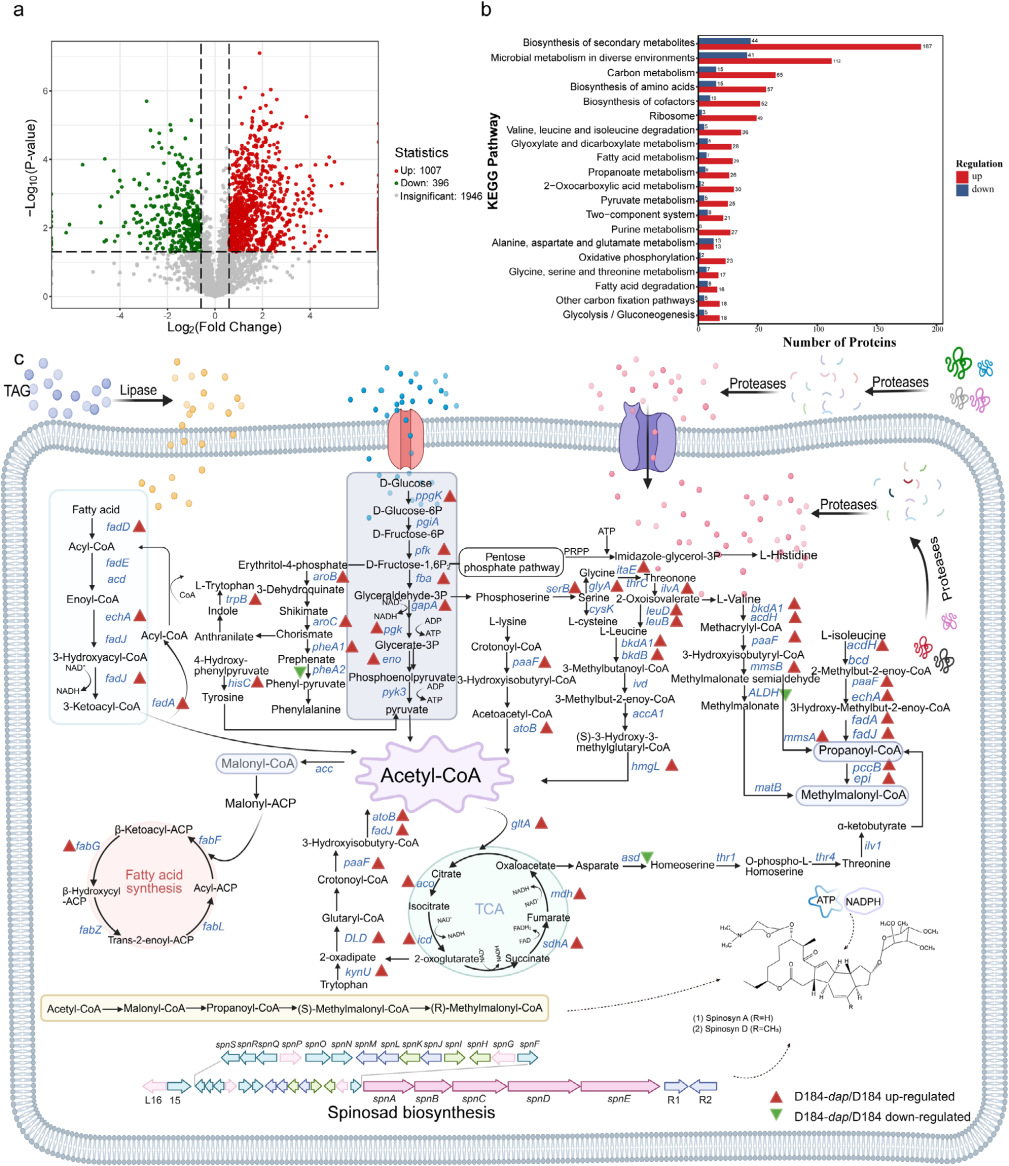
Proteomic analysis of high‐producing strain D184‐*dap* reveals protease‐centric metabolic rewiring for enhanced spinosad biosynthesis. (a) Volcano plot of differentially expressed proteins. The *x*‐axis represents the fold change of differential proteins, log‐transformed to base 2, while the *y*‐axis represents the *p*‐value, log‐transformed to base 10. Red and green dots indicate upregulated and downregulated proteins, respectively, with statistical significance based on the fold change and *p*‐value. (b) KEGG pathway classification comparison of upregulated and downregulated differentially expressed proteins. The *x*‐axis represents the number of differentially expressed proteins annotated to each KEGG functional category, while the *y*‐axis lists the names of the KEGG functional categories. Red bars indicate upregulated differentially expressed proteins, and blue bars represent downregulated differentially expressed proteins. (c) Metabolic network diagram for the biosynthesis of spinosad and related acyl‐CoA metabolites based on the KEGG pathway. The pathway map was constructed using quantitative proteomics data from day 4 of D184‐*dap* and D184 strains. Blue nodes represent genes encoding the corresponding proteins, while red arrows indicate proteins with upregulated abundance in D184‐*dap*, and green arrows represent proteins with downregulated abundance. Differential proteins were defined as upregulated (FC ≥ 1.5, *p*< 0.05) or downregulated (FC ≤ 0.67, *p*< 0.05). The pathway map emphasizes key metabolic pathways, including amino acid metabolism, carbon metabolism, and fatty acid metabolism, highlighting their role in the biosynthesis of spinosad.

As the strain with the highest spinosad‐titer, D184‐*dap* exhibited three key metabolic advantages: enhanced biomass accumulation, accelerated extracellular protein degradation, and elevated availability of key precursors and energy carriers. This unique combination of phenotypic traits and metabolic reprogramming makes D184‐*dap* an exemplary model for elucidating the mechanistic links among protease overexpression, metabolic network optimization, and enhanced spinosad biosynthesis. Quantitative proteomics analysis on day 4 identified 3349 proteins in D184 and D184‐*dap*; these included 1403 differentially expressed proteins (DEPs), of which 1007 were upregulated, and 396 were downregulated (Supporting Information and Figure 5a). Gene ontology (GO) enrichment analysis highlighted extracellular processes, glycolysis, and amino acid biosynthesis (Figure S20). KEGG analysis revealed DEP enrichment in secondary metabolite biosynthesis, carbon metabolism, and amino acid/fatty acid pathways (Figure 5b). Notably, the upregulated pathways included BCAA degradation and the metabolism of glycine/serine/threonine. These findings indicate the construction of a differential metabolic network that integrates central carbon, amino acid, and fatty acid metabolism (Figure 5c).

Proteomics analysis revealed comprehensive metabolic rewiring in D184‐*dap*, which was characterized by coordinated upregulation across amino acid, central carbon, and fatty acid pathways to support spinosad biosynthesis. Both the biosynthetic and catabolic pathways were reinforced in amino acid metabolism. Aromatic amino acid pathways showed elevated expression of biosynthesis enzymes (AroB, AroC, PheA1, TrpB, and HisC) and upregulated expression of degradation enzymes (KynU, DLD, PaaF, Hpd, Hgd, and PaaK), whereas branched‐chain amino acid metabolism exhibited parallel increases in biosynthesis (IlvC, IlvD, IlvE, and LeuD) and catabolism (BkdA1, BkdB, AcdH, PaaF, HmgL, and FadA). This dual activation was also observed in the glycogenic amino acid pathways (SerB, GlyA, and ItaE). Central carbon metabolism showed synchronized enhancement of glycolysis (Pgi, Pfk, GapA, and Pgk; validated by RT‐qPCR), the pentose phosphate pathway (Zwf and Gnd for NADPH), and the TCA cycle (GltA, Icd, FumA, and Mdh; validated by RT‐qPCR) to optimize energy and redox balance (Figure S21). Concurrently, fatty acid metabolism demonstrated coupled upregulation of β‐oxidation (FadJ, FadA) and fatty‐acid synthesis II (FabG) pathways, expanding the acetyl‐CoA pool (Table S8). This network‐wide metabolic remodeling, which linked extracellular protein degradation to intracellular precursor supply and energy cofactor generation, systematically optimized metabolic pathways, enabling D184‐*dap* to efficiently support spinosad biosynthesis.

### 3.5 Protease‐Mediated Cross‐Module Synergies Promote Spinosad Accumulation

**FIGURE 6:**
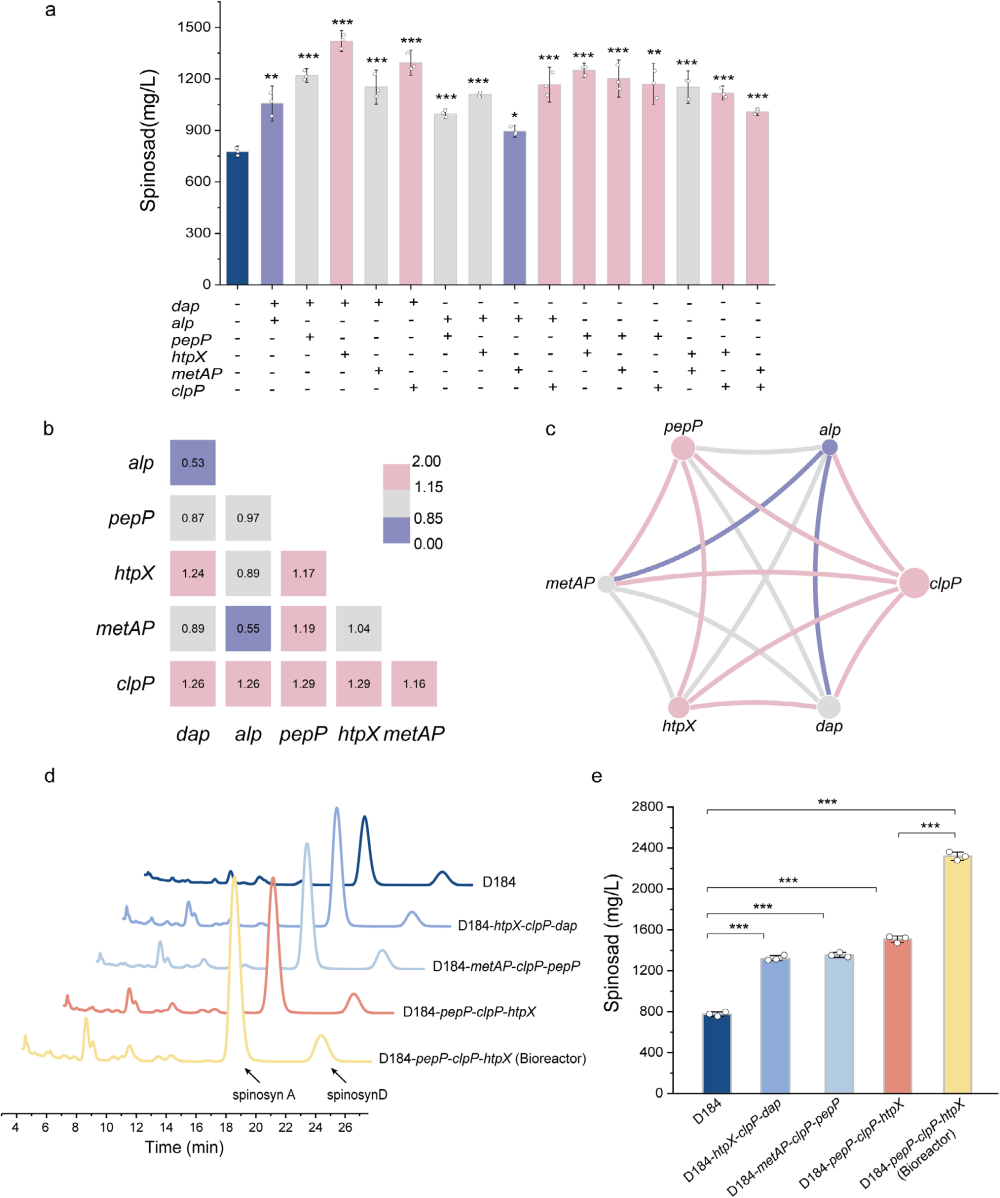
Protease‐mediated cross‐module synergies promote spinosad biosynthesis. (a) Titer of spinosad from 15 engineered strains generated by pairwise combinations of six genes (*dap*, *alp*, *pepP*, *htpX*, *metAP*, and *clpP*), quantified using UHPLC. (b) Gene interaction network based on synergy coefficients. The synergy coefficient *s* value represents the ratio of the sum of the individual gene effects to the combined effect value (the effects of the two genes on titer are independent events). When *s*<0.85, indicates antagonism; 0.85≤ *s*< 1.15, it indicates additivity; and *s* ≥ 1.15, it indicates synergism. (c) Heatmap of the pairwise gene interactions, where red indicates synergistic effects, gray denotes additive effects, and blue represents antagonistic interactions. Data are presented as the mean ± SD (n = 3 biological replicates). (d) Representative UHPLC chromatograms of spinosad produced by D184‐*metAP*‐*clpP*‐*pepP*, D184‐*htpX*‐*clpP*‐*dap*, and D184‐*pepP*‐*clpP*‐*htpX* in shake flask and D184‐*pepP*‐*clpP*‐*htpX* in 50L bioreactor cultures. (e) Titer of D184‐*metAP*‐*clpP*‐*pepP*, D184‐*htpX*‐*clpP*‐*dap*, and D184‐*pepP*‐*clpP*‐*htpX* in shake flask and D184‐*pepP*‐*clpP*‐*htpX* in 50L bioreactor cultures. Multiple comparison significance was tested to ^*^ *p*< 0.05, ^**^ *p*< 0.01, ^***^ *p*< 0.001 by one‐way ANOVA followed by Dunnett's/Tukey's post‐hoc test.

**FIGURE 7:**
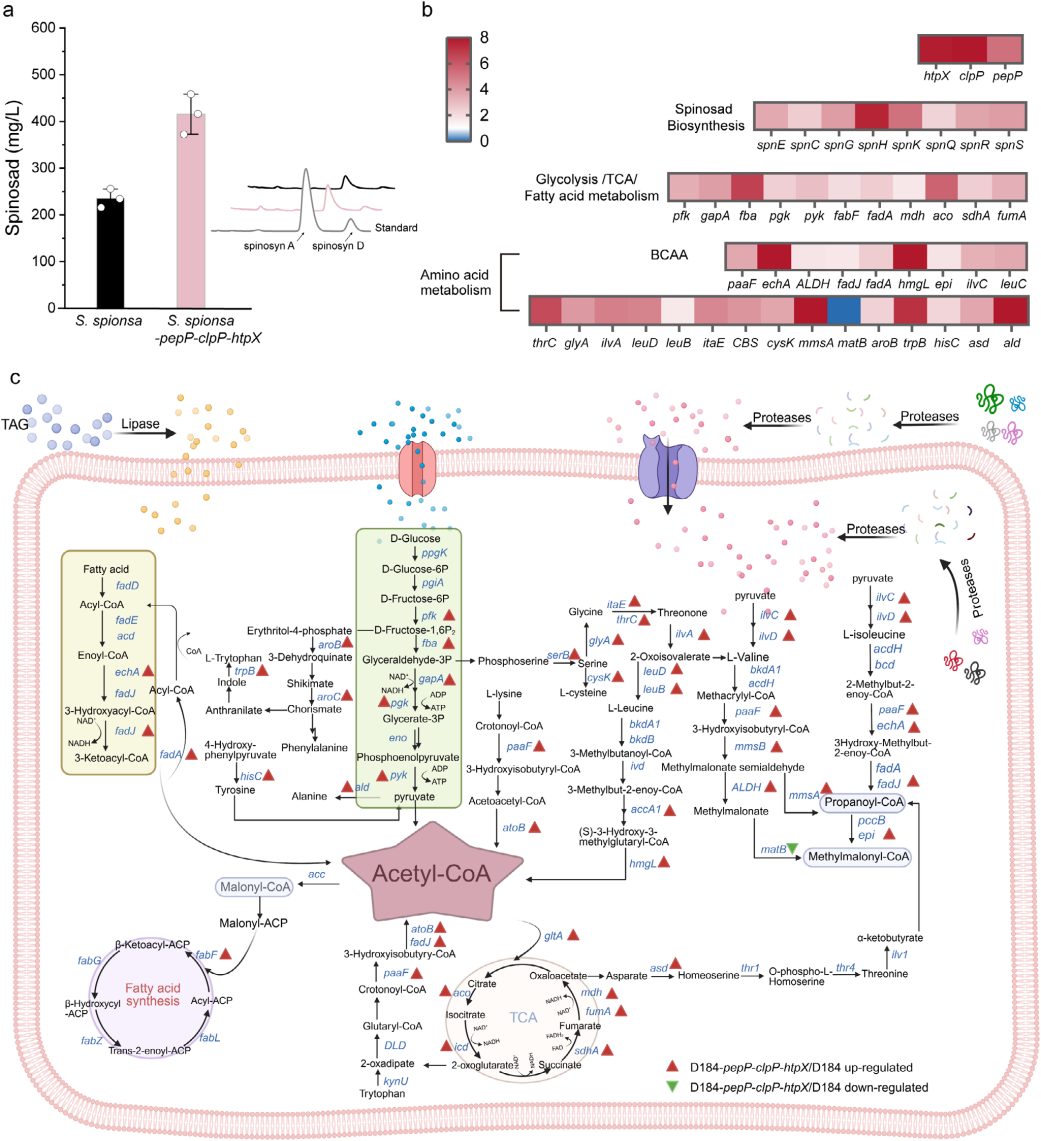
Combinatorial overexpression of *pepP*‐*clpP*‐*htpX* enhances spinosad biosynthesis and remodels the proteome and metabolic network in *S. spinosa*. (a) Spinosad titer and UHPLC chromatograms of *S. spinosa* overexpressing the *pepP*‐*clpP*‐*htpX* combination. Statistical significance determined using the *t*‐test (n = 3). ^*^ *p*< 0.05, ^**^ *p*< 0.01, ^***^ *p*< 0.001. (b) Protein abundance profiles of *spn* biosynthetic genes and selected key enzymes from representative metabolic pathways in the 96 h fermentation broth of D184‐*pepP*‐*clpP*‐*htpX* relative to the parental strain D184 (*p*< 0.05). (c) Metabolic network diagram for the biosynthesis of spinosad and related acyl‐CoA metabolites based on the KEGG pathway. The pathway map was constructed using quantitative proteomics data from day 4 of D184‐*pepP‐clpP‐htpX* and D184 strains. Blue nodes represent genes encoding the corresponding proteins, while red arrows indicate proteins with upregulated abundance in D184‐*pepP‐clpP‐htpX*, and green arrows represent proteins with downregulated abundance. Proteins were considered upregulated (FC ≥ 1.5, *p*< 0.05) or downregulated (FC ≤ 0.67, *p*< 0.05).

To quantify pairwise co‐expression effects, we selected the top six genes that most significantly increased the titer, spanning distinct functional modules: amino acid supply (*dap, pepP, metAP, alp*), membrane proteostasis (*htpX*), and ATP‐dependent proteolysis (*clpP*). We then constructed all the pairwise combinations (Figures S22 and S23), which yielded 15 engineered strains. Synergy was evaluated using an additivity‐based interaction metric, and summarized as a synergy index *s*. These 15 pairs are segregated into three classes. Two pairs exhibited antagonism (*dap‐alp* and *alp‐metAP*); five pairs displayed additive effects (*dap‐pepP*, *dap‐metAP*, *alp‐pepP*, *alp‐htpX*, and *htpX‐metAP*); and all remaining pairs demonstrated synergism. Among them, D184‐*dap*‐*htpX* achieved the highest titer, reaching 1420.82 ± 40.49 mg/L (Figure 6a). Notably, *clpP* paired with any other gene consistently produced synergy, whereas within‐module combinations in the amino acid supply set tended to be additive. Cross‐module pairings, such as *clpP* or *htpX* with amino acid supply genes, were more likely to yield synergy (Figure 6b,c). Additionally, as observed in the interaction network (Figure 6b), the gene combinations *pepP‐clpP‐htpX*, *metAP‐clpP‐pepP*, and *htpX‐clpP‐dap* exhibited synergistic triangular structures. The D184‐*metAP*‐*clpP*‐*pepP* strain reached a titer of 1352.01 ± 25.09 mg/L, which was higher than D184‐*metAP*‐*clpP* (1009.00 ± 13.86 mg/L), D184‐*clpP*‐*pepP* (1169.83 ± 79.93 mg/L), and D184‐*metAP*‐*pepP* (1202.04 ± 71.97 mg/L) (*p*< 0.05). D184‐*htpX*‐*clpP*‐*dap* increased the titer to 1318.67 ± 25.94 mg/L, but did not exceed the best‐performing pairwise construct D184‐*htpX*‐*dap* (1420.82 ± 40.49 mg/L) (Figure 6d,e; Figures S24 and S25). Notably, the most representative triangular combination, *pepP*‐*clpP*‐*htpX*, significantly increased the titer to 1507.60 ± 32.13 mg/L (*p*< 0.05), with the titer reaching 2318.87 ± 40.87 mg/L upon scale‐up fermentation in a 50L bioreactor (Figure 6d,e; Figure S26). In addition, we overexpressed the *pepP*‐*clpP*‐*htpX* combination in the wild‐type strain. The wild‐type strain produced 236.42 ± 20.05 mg/L, whereas the *S. spinosa*‐*pepP*‐*clpP*‐*htpX* strain reached a titer of 415.45 ± 43.02 mg/L (Figure 7a). Therefore, the synergistic effects of these combinations suggest that they collectively contribute to the titer enhancement.

To elucidate the molecular basis underlying the increased spinosad titer achieved by the *pepP*‐*clpP*‐*htpX* combination, we performed a quantitative proteomics analysis comparing D184‐*pepP*‐*clpP*‐*htpX* with D184 at 96 h, which identified 1886 differentially abundant proteins (1331 increased and 555 decreased) (Figure S27a). KEGG and COG enrichment analyses indicated broad metabolic reprogramming, with dominant enrichment in secondary metabolite biosynthesis, amino acid biosynthesis and degradation (notably BCAA metabolism), carbon metabolism, and lipid transport and metabolism (Figure S27b,c). At the pathway level, enzymes involved in glycolysis (Fba, Pfk, GapA, Pgk, and Pyk) and the TCA cycle (GltA, Aco, FumA, and Mdh) were coordinately elevated, which was accompanied by increased abundance of proteins involved in BCAA metabolism (IlvC, LeuC, IlvE, LeuB, LeuD, PaaF, HmgL, ALDH, and FadA) and aromatic and other amino acid pathways (AroB, TrpB, HisC, SerB, GlyA, ItaE, CysK, ThrC, and Ald), whereas the abundance of MatB was reduced. Fatty acid metabolism also shifted, with increased abundance of β‐oxidation enzymes (FadJ and FadA) and elevated FabF. Collectively, these results link the enhanced titer to coordinated remodeling of the primary metabolism and strengthened spinosad pathway capacity in the engineered strain (Figure 7b,c; Figure S28).

Only D184‐*clpP* exhibited an increased titer in the ATP‐dependent proteolysis module. ClpP constitutes the serine protease core [34], whereas ClpXP is among the most prevalent ATP‐dependent proteolytic machinery in bacteria [35]. Accordingly, we coexpressed *clpP* and *clpX* in D184 (Figure S29). As shown in Figure S30, the coexpression strain achieved a final spinosad titer (1033.74 ± 27.64 mg/L) significantly higher than both the parental D184 and the *clpP* single‐gene overexpression strain.

## 4 Discussion

*Streptomyces* species produce bioactive natural products, including spinosad, a polyketide insecticide with broad‐spectrum pest‐control potential for agricultural and industrial applications [36]. Despite the general understanding of spinosad biosynthesis, optimization of its synthesis remains challenging because of the complex regulatory mechanisms involved. Practical strain improvement often benefits from approaches combining enhanced biosynthetic capacity with the systematic interrogation of regulatory and metabolic constraints at the system level. Random mutagenesis technologies are efficient and practical entry points for obtaining strains with improved production [37, 38, 39]. In particular, ARTP mutagenesis has been successfully applied to enhance the productivity of diverse bacterial species, such as *Streptomyces* [40]*, Bacillus* [41], and *Lactobacillus* [42]. In this study, ARTP mutagenesis and high‐throughput screening led to the isolation of a highly performing mutant strain D184, which exhibited enhanced an spinosad titer (3.28 fold). Comprehensive characteristic analysis indicated that D184 possesses stronger metabolic and physiological properties than the wild type, including increased biomass accumulation, elevated intracellular acetyl‐CoA levels, and improved extracellular nitrogen utilization efficiency; these strengths were particularly evident in media rich in nitrogen sources. These superior phenotypic characteristics demonstrated the effectiveness of our combinatorial mutagenesis‐screening workflow for *S. spinosa* strain development.

Mutations in D184 relative to the wild‐type strain were enriched in amino acid metabolism and protease‐related functions, including SNPs in loci encoding Xaa‐Pro dipeptidyl‐peptidase and enoyl‐CoA hydratase, which may be linked to protein turnover, amino acid utilization, and acyl‐CoA metabolism. Since quantitative proteomics revealed the coordinated upregulation of 21 proteases and only *metAP*, *dpp*, and *ab*h harbored detectable mutations, the global protease increase was more consistent with regulatory rewiring than with structural changes in individual protease genes. In addition, D184 harbors mutations in 20 genes annotated as transcriptional regulators. Integration with the proteomics profiles highlighted an IclR‐family transcriptional regulator with increased abundance, suggesting that metabolic regulatory networks may be remodeled [43, 44] and could indirectly contribute to protease induction and nitrogen metabolism reconfiguration. Integrated proteomics and metabolomics analyses revealed that enhanced spinosad biosynthesis in D184 was primarily attributable to significant metabolic rewiring of amino acid pathways, particularly through rapid depletion of the intracellular valine, leucine, and isoleucine pools along with coordinated upregulation of BCAA degradation pathways, suggesting their preferential utilization as precursors for spinosad biosynthesis. Although upstream glycolytic intermediates decreased, the accumulation of downstream metabolites, particularly pyruvate, likely reflects convergent inputs from central carbon metabolism and amino acid‐derived routes. In contrast to previous studies, which mainly attributed the improvement of spinosad to glycolysis or fatty acid degradation [10, 16], our multi‐omics data delineated a distinct metabolic profile in D184, characterized by pronounced protease‐driven nitrogen utilization and amino acid recycling, which occurred in parallel with ongoing central carbon metabolism. Collectively, these findings support tighter coupling between protease‐driven amino acid turnover and spinosad biosynthesis in D184. On the basis of these findings, we propose that engineering the protease network in D184 could enhance the amino acid pool and precursor pathways, thereby enhancing the underlying metabolic mechanisms of spinosad biosynthesis.

Functional annotation indicated that the 21 upregulated proteases were predominantly associated with nitrogen acquisition and recycling, spanning extracellular degradation, substrate transport, and intracellular assimilation, supporting a strengthened nitrogen recycling capacity in the high‐titer strain. Genetic validation through overexpression studies revealed that eight of these engineered strains exhibited enhanced spinosad titers (D184‐*dap*, D184‐*alp*, D184‐*pepP*, D184‐*htpX*, D184‐*metAP*, D184‐*clpP*, D184‐*pepN*, D184‐*ybbJ*), while CRISPRi‐mediated knockdown of the corresponding genes consistently reduced titers (with a few exceptions), confirming that these proteases are legitimate metabolic engineering targets. Notably, the high‐titer strains exhibited three key metabolic features: enhanced extracellular protease levels and protein hydrolysis, elevated levels of critical cofactors (ATP and NADPH), and expanded acyl‐CoA precursor pools. Transcriptional analysis revealed coordinated upregulation of key *spn* pathway genes (*spnB*, *spnC*, *spnE*) and critical amino acid metabolic nodes (*thrC, ivd, and cysK*). Targeted overexpression of *thrC* [45], *ivd* [46], and *cysK* [47] further confirmed their roles in enhancing precursor availability and cofactor regeneration. These changes suggest that protease overexpression not only facilitates the degradation of extracellular proteins but also triggers amino acid metabolism pathways and a broader spinosad biosynthetic network. In the high‐titer strain D184‐*dap*, DIA‐based proteomic profiling [48] revealed systematic metabolic reprogramming characterized by coordinated upregulation of glycolytic and pentose phosphate pathway enzymes, selective activation of specific TCA cycle components, and significant enhancement of amino acid metabolic flux. This systematic reconfiguration established an optimized metabolic network that simultaneously provided essential biosynthetic precursors (acetyl‐CoA and malonyl‐CoA) and maintained adequate NADPH supply, thereby creating favorable conditions for sustained spinosad biosynthesis. In contrast, the other strains exhibited no significant change or even a decrease in titer, a phenomenon potentially attributable to substrate specificity constraints or metabolic burden effects. These observations suggest that indiscriminate pathway upregulation, particularly targeting non‐limiting or redundant enzymatic steps, may not increase the spinosad titer and could paradoxically limit spinosad synthesis efficiency owing to energy competition and resource allocation [49].

Although these findings highlight the potential of metabolic engineering for individual targets, selecting synergistic combinations remains a challenge in combinatorial engineering [50]. Therefore, investigating the potential functional relationships between individual beneficial targets and constructing interaction maps will undoubtedly provide rational guidance for multi‐target combinations, thereby facilitating efficient and continuous improvement of strains [31, 51, 52]. Using a framework of three resource and flux modules, namely membrane proteostasis, ATP‐dependent proteolysis, and amino acid supply, we constructed a synergy map that delineates their orthogonality and complementarity. Cross‐module pairs, especially *clpP* with amino acid metabolism genes or with membrane proteostasis genes, were predominantly synergistic. This finding suggests that the cross‐module co‐expression of these genes may facilitate spinosad biosynthesis through coordinated optimization of protein homeostasis, enhanced cellular stability, and increased precursor availability. The *dap‐htpX* combination delivered the greatest gain (1420.82 mg/L), indicating that expanding precursor supply while strengthening proteostasis and inlet flux jointly relieves synthetic bottlenecks. Within‐module combinations (for example, *dap*, *pepP*, and *metAP* within the amino acid supply module) were mostly additive, consistent with a saturating, diminishing‐return response. Combinations including *alp* occasionally showed antagonism, suggesting that the front‐end endoproteolysis step on the amino acid metabolism module requires an upper bound on expression to avoid substrate mismatch or stress overload. Based on these observations, we established quantitative design rules, namely synergy, additivity, and antagonism, to guide engineering strategies. We recommend prioritizing cross‐module pairings, limiting *alp* expression to avoid conflicts, and adopting multigene parallel designs with parameterized bounds. These rules link candidate correlations to causally supported cross‐module interactions and guide rational combinatorial design and validation under the D184 strain background and process conditions. In comparison with the *metAP*‐*clpP*‐*pepP* and *htpX*‐*clpP*‐*dap* combinations, the *pepP*‐*clpP*‐*htpX* combination, which represents the most typical triangular structure, achieved the highest titer, increasing the spinosad titer 6.38 fold and delivering a stable 9.81 fold improvement upon scaling‐up to a 50L bioreactor. Proteomics profiling of the 96 h fermentation samples showed that D184‐*pepP*‐*clpP*‐*htpX* revealed broad enrichment of the amino acid metabolism and BCAA degradation pathways relative to D184, together with an elevated abundance of glycolytic and TCA cycle enzymes, indicating that enhanced nitrogen‐associated turnover operates alongside maintained central carbon metabolism. Importantly, multiple proteins encoded by the spinosad biosynthetic gene cluster were also increased, indicating that protease‐driven remodeling is coupled with strengthened biosynthetic capacity at the pathway level [16]. Additionally, overexpression of the *pepP*‐*clpP*‐*htpX* protease combination increased the titer in the wild‐type strain, suggesting that this synergistic triangular structure likely operates through general metabolic nodes rather than D184‐specific effects. Notably, however, the higher titer in D184 indicated that the magnitude of the response was strongly strain dependent, consistent with the benefit amplification attributable to D184's pre‐optimized metabolic fluxes and precursor supply capacity. These findings suggest that rational design and optimization of synergistic gene combinations can offer more effective strategies for enhancing titers in complex metabolic networks, and that cross‐module interactions are not necessarily additive but are often combination‐specific and host‐dependent.

Within the ATP‐dependent proteolysis module, ClpP is the core degradative subunit of bacterial proteostasis [53, 54]. Overexpression of the core protease subunit ClpP was shown to significantly increase spinosad titers in *S. spinosa*. In contrast, excessive expression of AAA+ unfoldases such as ClpX, ClpC, or ClpA/B consumes ATP and may accelerate the turnover of positive regulators, thereby reducing product formation. Unlike previous reports wherein ClpX overexpression stimulates actinorhodin in *S. lividans* or *S. coelicolor* [55], the expression of *clpX* in D184 from a strong constitutive promoter may have exceeded the optimal ClpP to ClpX ratio, leading to ATP‐intensive proteolysis and a decrease in the spinosad titer [56]. Moreover, given prior evidence that ClpXP mediates the degradation of AtrA to enhance daptomycin production in *S. roseosporus* [35], we coexpressed *clpP* and *clpX*, which increased the spinosad titer, highlighting the importance of balancing the protease and its unfoldase.

We propose a flux‐reprogramming workflow wherein specific proteases enhance spinosad biosynthesis by increasing precursor supply, modulating transcriptional networks, and optimizing key metabolic steps, thereby exerting multilayer control. However, the network‐level interactions between proteases and their metabolic pathways remain unclear. Future studies should focus on validating the role of transcriptional regulators in mediating the dual regulatory effects of proteases, particularly by characterizing how they influence key metabolic pathways and precursor supply and ultimately affect product synthesis. Notably, the IclR‐family of regulators identified here should be prioritized for focused genetic and molecular validation. Furthermore, comprehensive protein‐protein interaction analyses, such as coimmunoprecipitation (Co‐IP) and mass spectrometry, can help elucidate the interactions between proteases and other key proteins within metabolic pathways. Since protease mechanisms may vary across microbial strains, the generalizability of the observed synergistic effects should be validated in additional strains, and future studies should consider developing protease regulation strategies tailored to each strain to optimize the biosynthesis of target metabolites.

## 5 Conclusion

Based on the development of a high‐performing strain with enhanced extracellular nitrogen utilization via ARTP mutagenesis, this study proposes and validates a secondary metabolism optimization workflow that integrates protease genetic manipulation, multi‐omics analysis, and rational design to elucidate the multilayered roles of proteases in metabolic regulation. Through overexpression and pairwise synergy analysis of protease‐related genes, we separated and quantified the three flux modules, namely membrane proteostasis, ATP‐dependent proteolysis, and amino acid supply, thereby establishing an evidentiary link from correlation to causality. The proposed and experimentally validated synergistic triangular combinations improved spinosad biosynthesis by providing causal evidence that dynamic inter‐module crosstalk governs nitrogen‐dependent metabolic flux. Future work should resolve fine‐scale interactions and species‐specific features of protease and metabolism networks to guide tailored control strategies and further increase target product titer.

## Author Contributions

Liqiu Xia and Jie Rang supervised the project, managed the administration, and secured funding. Liqiu Xia and Duo Jin generated the concepts and designed the research. Duo Jin and Wangqiong Chen performed mutant construction. Duo Jin, Zirui Dai, and Baolong Bai performed UHPLC analysis. Duo Jin, Qing Liu, Xirong Liu, and Jie Rang contributed to omics analysis, data collection, and formal analysis. Duo Jin, Jie Rang, and Zirong Zhu wrote the manuscript. All authors read and approved the manuscript.

## Ethical Statement

This article does not involve any studies with human participants or vertebrate animals performed by any of the authors. The study involves experiments with *H. armigera* larvae.

## Conflicts of Interest

The authors declare that they have no conflict of interest.

## Supporting information




**Supporting File 1**: advs74210‐sup‐0001‐SuppMat.docx.

**Supporting File 2**: advs74210‐sup‐0002‐SuppMat.xlsx.

## Data Availability

The data that support the findings of this study are available in the supplementary material of this article.
