# Supplementary material for: Systematic Engineering of Proteases in Saccharopolyspora Spinosa Reveals Synergistic Enhancement of Spinosad Biosynthesis via Substrate Flux Optimization
Source: Adv Sci (Weinh). 2026 Jan 31;13(20):e22638. doi: 10.1002/advs.202522638 (PMC13067860; doi:10.1002/advs.202522638)
Supplement: Supplementary file 1 — Supporting File 1: advs74210‐sup‐0001‐SuppMat.docx. [file ADVS-13-e22638-s002.docx]

**Supplementary Material**

**Materials and Methods**

**1. Strains and growth conditions**

All strains used in this study are listed in Supplementary **Table S1**. The activation medium for the wild-type strain of *S. spinosa* (CCTCC M206084) and its derivatives was CSM containing 10 g/L glucose, 45 g/L trypticase soy broth, 9 g/L yeast extract, and 2.2 g/L MgSO₄·7H₂O. The fermentation medium comprised the base fermentation medium (BFM) (20 g/L glucose, 4 g/L tryptone, 4 g/L yeast extract, 1 g KNO₃, 0.5 g/L MgSO₄·7H₂O, 0.5 g/L K₂HPO₄·3H₂O, 0.01 g/L FeSO₄·7H₂O), as well as the modified YHM (20 g/L glucose, 22.5 g/L cottonseed meal, 5 g/L soybean powder, 2 g/L yeast extract, 10 g/L soluble starch, 7 g/L corn syrup and 5 g/L CaCO₃, pH 7.5). Culture conditions were maintained at 28 °C and 230 rpm. *Escherichia coli* was cultured in LB medium at 37 °C and 150 rpm, with the addition of the antibiotic apramycin (50 μg/ml) to the medium as required.

The seed culture was transferred to a 50L fermenter with 10% inoculum, using optimized YHM medium. The fermentation was performed at 28°C, with a tank pressure of 0.03-0.05 MPa and an airflow rate of not more than 1.5 vvm. The pH was maintained around 7.5, with dissolved oxygen above 60%. Antifoam was added as needed. The fermentation lasted 192-240 hours, with agitation at 150-220 rpm. A feed of corn steep liquor (7 g/L) was added at 96 hours, comprising 5% of the total fermentation volume.

**2. Morphological and physiological phenotyping**

**2.1 Phase-contrast and SEM imaging.** Mycelial morphology was examined by phase-contrast microscopy (AXIO Scope A1, Zeiss, Germany). Briefly, cultures grown for 48 h on CSM agar were inspected for purity, and 50 µL of cell suspension was streaked onto TSB agar to confirm the absence of contamination and to assess sporulation after 3-6 d at 28 °C. For scanning electron microscopy (SEM), sterile coverslips were inserted into freshly inoculated TSB agar at a 45° and incubated for 4 d. For the bacterial suspension, the bacterial cell precipitates were resuspended in 2.5% glutaraldehyde fixative solution. Coverslips were then retrieved, air-dried, sputter-coated with gold, and imaged on an electron microscope (SEM, Hitachi, Shinagawa-ku SU8010, Japan)

**2.2 Biomass determination.** Fermentations were carried out in the modified YHM medium as described above. Every 2 days, aliquots were withdrawn and cells were collected by centrifugation, and the pellets were lyophilized to a constant mass. Dry cell weight (DCW) was recorded; three biologically independent cultures were analyzed for each strain.

**2.3 Glucose quantification (DNS assay).** Supernatants were collected every 24 h during fermentation. For the 3,5-dinitrosalicylic acid (DNS) assay, 1.0 mL of appropriately diluted supernatant was mixed with 2.0 mL DNS reagent, boiled for 3 min, rapidly cooled, and brought to volume with 12 mL ultrapure water. Absorbance at 540 nm was measured, and glucose concentrations were determined from a calibration curve, correcting for dilution.

**2.4 Total extracellular protease activity.** Total extracellular protease activity was measured using a Total Protease Activity Assay Kit (Jiangsu Aidisheng Biological Technology Co., Ltd.) according to the manufacturer’s instructions. Total extracellular protease activity was determined by measuring the absorbance at 366 nm of chromogenic products generated from azo-casein hydrolysis catalyzed by cell-free fermentation supernatants collected on days 2, 4, 6, and 8.

**2.5 Extracellular protein quantification.** Total protein in culture supernatants was measured daily using the Micro BCA Protein Assay Kit (Sangon Biotech, China) according to the manufacturer’s instructions. All measurements were performed with three biological replicates.

**3. Genome resequencing and variant analysis**

The raw image data generated by Next Generation Sequencing (NGS) were processed through base calling to obtain the original sequencing reads, referred to as Raw Data or Raw Reads. The raw sequencing data were then filtered using fastp to yield high-quality Clean Reads, with duplicate reads removed for subsequent analysis. All downstream analyses were performed based on the Clean Reads. The sequencing data were aligned to the reference genome of *Saccharopolyspora spinosa* (NZ_CP171362.1) (<https://www.ncbi.nlm.nih.gov/nuccore/NZ_CP171362.1/>). The alignment rate provides insights into the similarity between the sample and the reference genome, while the sequencing depth and coverage reflect the uniformity of the sequencing data and its homology to the reference sequence. SNPs were detected using Snippy, while SnpEff was employed for annotation of SNPs and indels. Structural variants (SVs) were identified using Delly, and SVs were annotated with SnpEff.

**4.** **DIA-based quantitative proteomics: acquisition and analysis**

**4.1 Sample preparation: cell lysis, protein extraction, and digestion.** Cell pellets stored at −80 °C were resuspended in lysis buffer (8 M urea, 1 mM PMSF, 2 mM EDTA) and sonicated on ice for 5 min. Lysates were clarified by centrifugation (15,000 g, 4 °C, 10 min), and protein concentration in the supernatant was determined by BCA assay. For reduction-alkylation and digestion, 100 μg of protein was brought to 200 μL with 8 M urea, reduced with 5 mM DTT for 45 min, and alkylated with 11 mM iodoacetamide for 15 min in the dark. Samples were diluted with 800 μL of 25 mM ammonium bicarbonate, supplemented with 2 μL sequencing-grade trypsin (Promega, V5280), and incubated overnight at 37 °C. Digests were acidified to pH 2–3 with 20% (v/v) TFA, desalted on C18 sorbent, and peptide concentration was measured using a Pierce™ peptide quantitation kit.

**4.2 LC–MS/MS data-independent acquisition.** Peptides were separated on a Vanquish Neo UHPLC nano-LC system using mobile phase A (0.1% formic acid in water) and B (0.1% formic acid in acetonitrile). A trap-and-analytical two-column configuration was employed (trap: PepMap Neo Trap Cartridge, 300 μm × 5 mm, 5 μm; analytical: Easy-Spray PepMap Neo UHPLC, 150 μm × 15 cm, 2 μm; column temperature 55 °C). The flow rate was 2.5 μL min⁻¹ with an effective gradient of 6.9 min and a total run time of 8 min. MS data were acquired on an Orbitrap Astral mass spectrometer operating in positive-ion mode. Full MS1 scans covered m/z 380–980 at a resolution of 240,000 (at m/z 200), with an AGC target set to 500% and a maximum injection time (IT) of 5 ms. MS2 spectra were collected in DIA mode using 299 isolation windows (2 Th each), with HCD collision energy of 25%, normalized AGC target 500%, and maximum IT of 3 ms.

**4.3 DIA quantification and downstream analysis.** Protein quantification was based on peptide-level DIA intensities. For pairwise comparisons, protein abundance values between groups were evaluated using a two-sided Student’s t-test, and P-values were reported. Identified proteins and differentially abundant proteins were annotated across multiple dimensions, including Gene Ontology (GO), COG functional categories, KEGG pathways, protein domains, subcellular localization, and signal peptides (SignalP). Enrichment analyses of GO, COG, KEGG, eggNOG and protein domain categories were performed using a hypergeometric test to assess statistical significance of overrepresentation. Differential proteins were defined by a two-sided t-test with thresholds of FC ≥ 1.5 or FC ≤ 0.6667 and *P* < 0.05

**5.** **Metabolomics acquisition and analysis**

**5.1 Sample Preparation.** Samples were retrieved from the -80°C freezer and thawed on ice. A 50 mg aliquot was weighed, mixed with 100 μL of physiological saline, and vortexed. A 50 μL aliquot was used for protein quantification, while another 50 μL was mixed with 200 μL methanol-based internal standard solution and vortexed for 2 minutes. The samples were rapidly frozen in liquid nitrogen for 5 minutes, thawed on ice, and vortexed; this process was repeated three times. After centrifugation at 12,000 rpm for 10 minutes at 4°C, the supernatant was filtered through a protein precipitation filter and stored at -20°C until analysis.

**5.2 Sample processing.** The data acquisition system consisted of an Ultra Performance Liquid Chromatography (UPLC) system (ExionLC™ AD) coupled with Tandem Mass Spectrometry (MS/MS) (QTRAP® 6500+). The liquid chromatography was performed using an ACQUITY BEH Amide column (1.7 µm, 100 mm × 2.1 mm i.d.) with a mobile phase consisting of Phase A (ultra-pure water containing 2 mM ammonium acetate and 0.04% formic acid) and Phase B (acetonitrile containing 2 mM ammonium acetate and 0.04% formic acid). The gradient elution program was as follows: 0–1.2 min with A/B 10:90 (V/V), 1.2–9 min with A/B 40:60 (V/V), 9–10 min with A/B 60:40 (V/V), and 10.01–15 min with A/B 10:90 (V/V). The flow rate was set at 0.4 mL/min, the column temperature was maintained at 40°C, and the injection volume was 2 μL. For mass spectrometry, electrospray ionization (ESI) was used at a temperature of 550°C, with the positive ion mode set at 5500 V and the negative ion mode at -4500 V. The curtain gas (CUR) was set to 35 psi. Each ion pair was scanned according to optimized declustering potential (DP) and collision energy (CE) settings on the Q-Trap 6500+ mass spectrometer.

**5.3 Data Processing.** Mass spectrometry data were processed using MultiQuant 3.0.3 software. A Metware Database (MWDB) was constructed based on standards for qualitative analysis. Quantification was performed using the Multiple Reaction Monitoring (MRM) mode of the triple quadrupole mass spectrometer. Multivariate data analysis was carried out using the R package metabolomics for principal component analysis (PCA) and orthogonal partial least squares discriminant analysis (OPLS-DA) to explore differences between samples.

**5.4 Statistical Analysis.** Statistical significance between groups was determined using a two-tailed Student’s *t*-test, with a significance threshold of *P* < 0.05. fold change (FC) values FC ≥ 1.5 were considered upregulated, while FC ≤ 0.6667 were considered downregulated. All data are presented as the mean ± standard deviation (SD) from at least three biological replicates.

**Results**

**3.1 Ultrastructural analysis**

To compare morphological architecture between the parental strain D184 and its eight engineered strains, aerial structures were examined by scanning electron microscopy (SEM). All strains displayed the genus-typical Saccharopolyspora phenotype, with extensively branched aerial hyphae and multiseptate, helical spore chains. No significant differences were observed in hyphal bundling, spore-chain length, or helical pitch between engineered strains and D184. By contrast, the engineered strains consistently exhibited a lower density of spine-like surface ornamentation on spores, yielding a smoother spore surface relative to D184. These features point to subtle remodeling of surface morphogenesis or envelope maturation associated with the genetic interventions (**Fig. S12**).

**3.2 Spore morphology and developmental program**

To quantify changes in sporulation capacity and their regulatory basis, we combined plate-based phenotyping with RT-qPCR of six developmental regulators (*ssgA*, *whiA*, *whiB*, *bldD*, *wblE*, *sigF*). On TSB agar, most engineered strains exhibited altered sporulation by day 4 relative to the parental strain D184. Notably, D184-*dap*, D184-*pepP*, D184-*metAP*, and D184-*clpP* showed earlier formation of aerial mycelium and spore layers with more pronounced whitening, consistent with an accelerated developmental trajectory, whereas several other derivatives displayed delayed or sparse sporulation (**Fig. S13**).

Functionally, *ssgA* promotes septation and spore maturation, *whiA* and *whiB* are indispensable for initiation and morphogenesis of spores, with *whiB* additionally implicated in the onset of antibiotic production, *wblE* and *sigF* contribute to oxidative-stress responses and late-stage sporulation, processes that often coincide with enhanced secondary-metabolic activity and spore robustness. By contrast, *bldD* encodes a global developmental repressor; reduced *bldD* expression typically precedes earlier activation of differentiation and antibiotic biosynthesis ^[1]^. Transcript levels paralleled plate phenotypes (**Fig. S14**). Strains with enhanced sporulation showed broad upregulation of *ssgA*, *whiA*, and *whiB*, accompanied by no significant change or downregulation in *bldD* expression; in a subset, *wblE* and *sigF* were also upregulated, consistent with strengthened stress tolerance and late maturation. Conversely, strains with reduced sporulation (D184-*alp*, D184-*htpX*, D184-*pepN*, D184-*ybbJ*) displayed coordinated downregulation of key pro-developmental regulators, particularly *ssgA* and *whiB*, along with an increase in *bldD* expression; *wblE* and *sigF* did not show consistent upregulation in these strains. Taken together, this transcriptional signature aligns with the phenotypic grouping observed on plates and mirrors the direction of titer changes across the strains.

**3.3 Biomass Accumulation Kinetics**

Throughout the 12-day fermentation, all eight protease overexpression strains exhibited growth patterns similar to the parental strain D184, indicating that the high-level expression of these genes has a neutral metabolic burden on cell growth. During the first 6 days, accumulation in the D184-*dap* and D184-*alp* strains was significantly higher than in D184, suggesting that the overexpression of most genes had not yet fully exerted its beneficial effect on cell proliferation during the early growth phase. By day 10, except for D184-*clpP*, which exhibited significantly lower biomass than D184, all other strains associated with increased spinosad titer maintained or further expanded the biomass gap compared to D184. This suggests that the impact of overexpressing different protease-related genes on cell proliferation is strain-specific and time-dependent (**Fig. S15**).

We further investigated the remaining 13 protease overexpression engineered strains that did not exhibit an increase in spinosad biosynthesis. Biomass accumulation measurements were performed, and it was found that 4 of these strains showed no significant difference in growth compared to the wild-type strain. With this consistency, spinosad biosynthesis in these strains also showed no significant improvement compared to the wild-type. In contrast, the growth of the remaining 9 strains was lower than that of the wild-type strain (**Fig. S16**). This decline in growth and titer may be attributed to the metabolic burden associated with protease overexpression, which can increase energy demand and intensify competition for limiting resources such as ATP, NADPH, and acyl-CoA, thereby altering resource allocation away from biomass formation and other essential metabolic processes and ultimately constraining spinosad biosynthesis in these strains. These results suggest that optimizing the balance between protease overexpression and cell growth is crucial for improving spinosad titer.

**3.4 Carbon source utilisation**

To systematically evaluate glucose utilization during fermentation, the residual glucose concentration in the fermentation supernatant was measured daily over 12 days using the DNS (3,5-dinitrosalicylic acid) method (**Fig. S15**). The results revealed significant temporal heterogeneity in the engineered strains. The glucose consumption rate of D184-*dap* was higher than that of D184 during the exponential growth phase. In contrast, D184-*metAP* and D184-*clpP* displayed a distinct “fast early, slow late” pattern: glucose consumption was rapid from days 4 to 6, but declined significantly after day 7, even falling below the level observed in the parental strain. No significant differences in glucose consumption rate were observed between other engineered strains and D184.

Mechanistically, we structured our analysis around four processes, including selective proteolysis, precursor generation and reassimilation, energy and reducing power coordination, and *spn* biosynthetic pathway activation, and used this framework to guide systematic characterization of the engineered strains and the integrative analysis of the resulting multidimensional datasets

**3.5 Transcriptional regulation of the TCA cycle and fatty acid metabolic pathways**

Day 4 RT-qPCR for *gltA*, *aceK*, *fadA*, and *fadD* revealed three reproducible response patterns across the engineered strains. The gene *gltA* encodes citrate synthase and sets entry and flux control for the tricarboxylic acid cycle, abbreviated TCA. The gene *aceK* encodes the isocitrate dehydrogenase kinase–phosphatase that partitions carbon between the TCA cycle and the glyoxylate shunt. The genes *fadA* and *fadD* encode, respectively, the terminal thiolase of beta oxidation and the long-chain acyl-CoA synthetase that initiates beta oxidation. Relative to D184, in D184-*alp* and D184-*dap*, *gltA*, f*adA* and *fadD* were coordinately upregulated, indicating simultaneous reinforcement of carbon mobilization and central-carbon throughput (**Fig.4i**). D184-*ybbJ* primarily showed an upregulation of *aceK* and no significant changes in *fadA* and *fadD*, consistent with carbon reallocation via mid-cycle restriction of TCA flux rather than activation of fatty-acid catabolism. Across D184-*pepP*, D184-*htpX*, D184-*clpP*, D184-*pepN*, and D184-*metAP*, *aceK* transcripts were upregulated, whereas *gltA* was downregulated or unchanged; in parallel, *fadA* and *fadD* were upregulated relative to D184. This asymmetric pattern indicates tempered TCA flux coupled to enhanced β-oxidation, biasing carbon skeletons toward biosynthetic branches with lower ATP cost and improved carbon-use efficiency. The transcriptional features tracked dry-cell-weight kinetics, with D184-*dap* and D184-*alp* showing higher growth rates initially (**Fig. S15**).

**Figure**


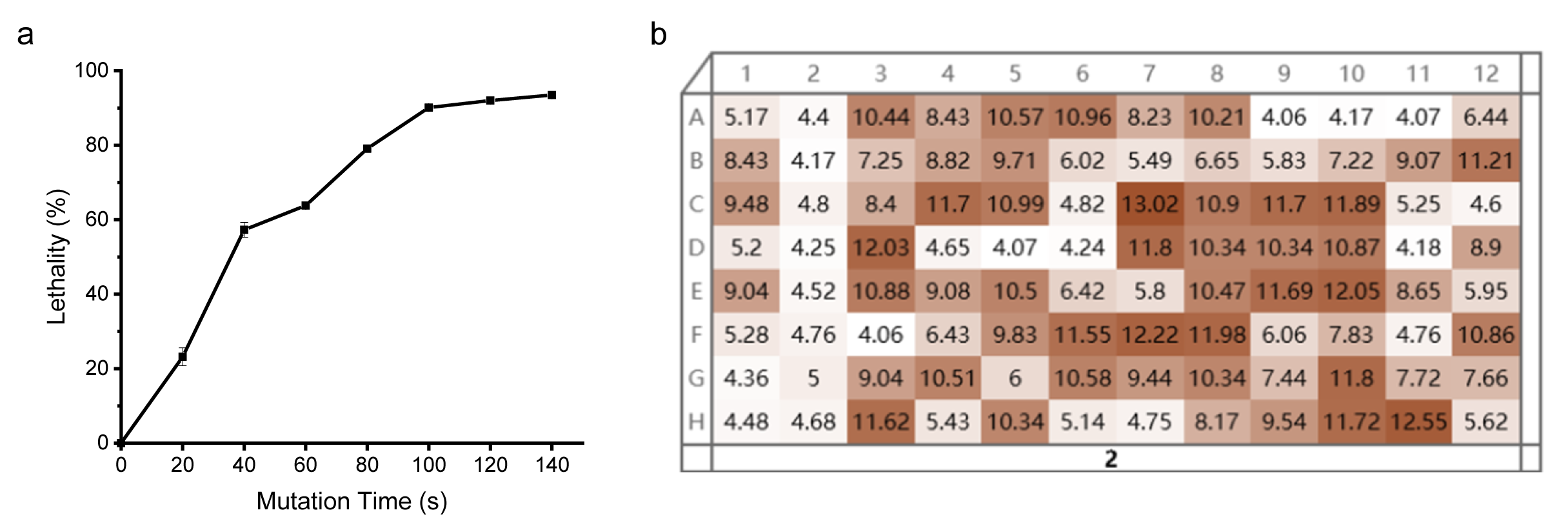


**Fig. S1 Lethality curve of *S. spinosa* wild-type strain induced by ARTP mutagenesis and representative results from the OD_600_-based screening.**

**
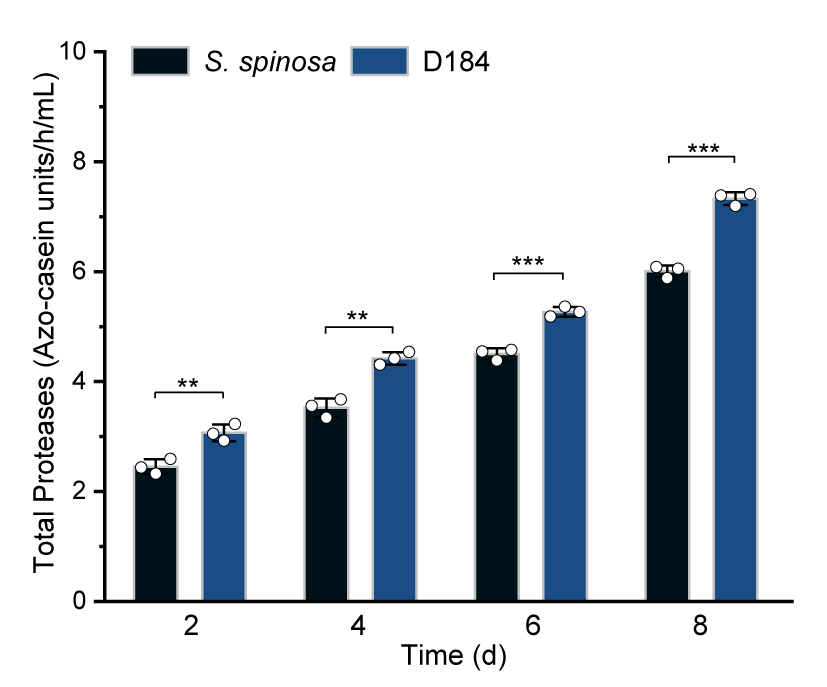
**

**Fig. S2 Total extracellular protease activity in the wild-type and D184 strains during fermentation on days 2, 4, 6, and 8. Statistical significance determined using the *t*-test (n = 3). **P* < 0.05, ***P* < 0.01, ****P* < 0.001.**


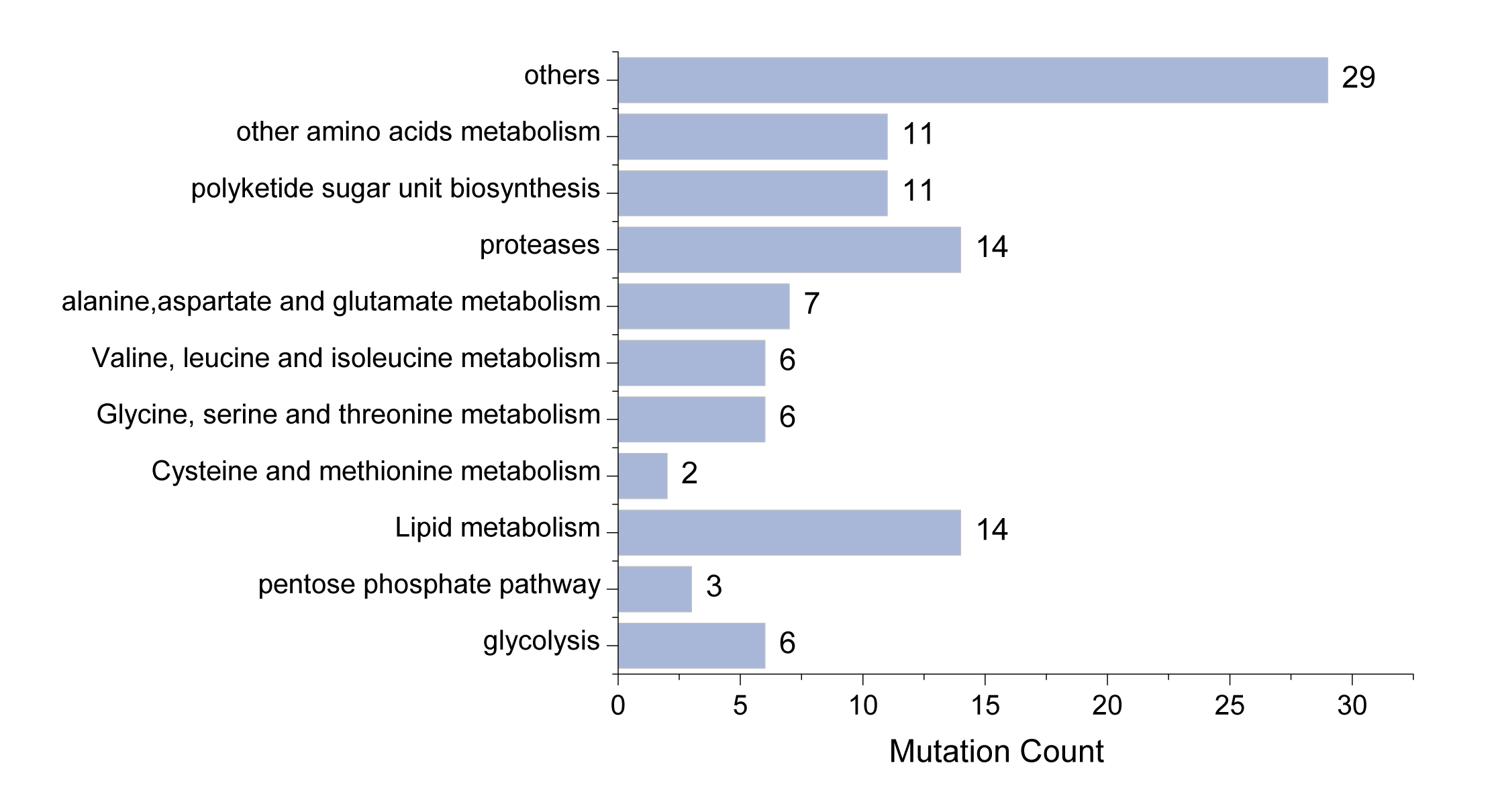


**Fig. S3 SNP-based analysis of key metabolic pathways in the mutant strain D184 and wild-type *S. spinosa*.** The figure focuses solely on genes participating in the metabolic pathways and excludes hypothetical proteins.





**Fig. S4 Intracellular fatty acid content in *S. spinosa* and D184 on day 4.** Fatty acid levels were quantified in the intracellular fractions of *S. spinosa* and the ARTP-mutant strain D184 on the fourth day of culture. Data represent the mean ± standard deviation of triplicate measurements. Statistical significance determined using the *t*-test (n = 3). **P* < 0.05, ***P* < 0.01, ****P* < 0.001.


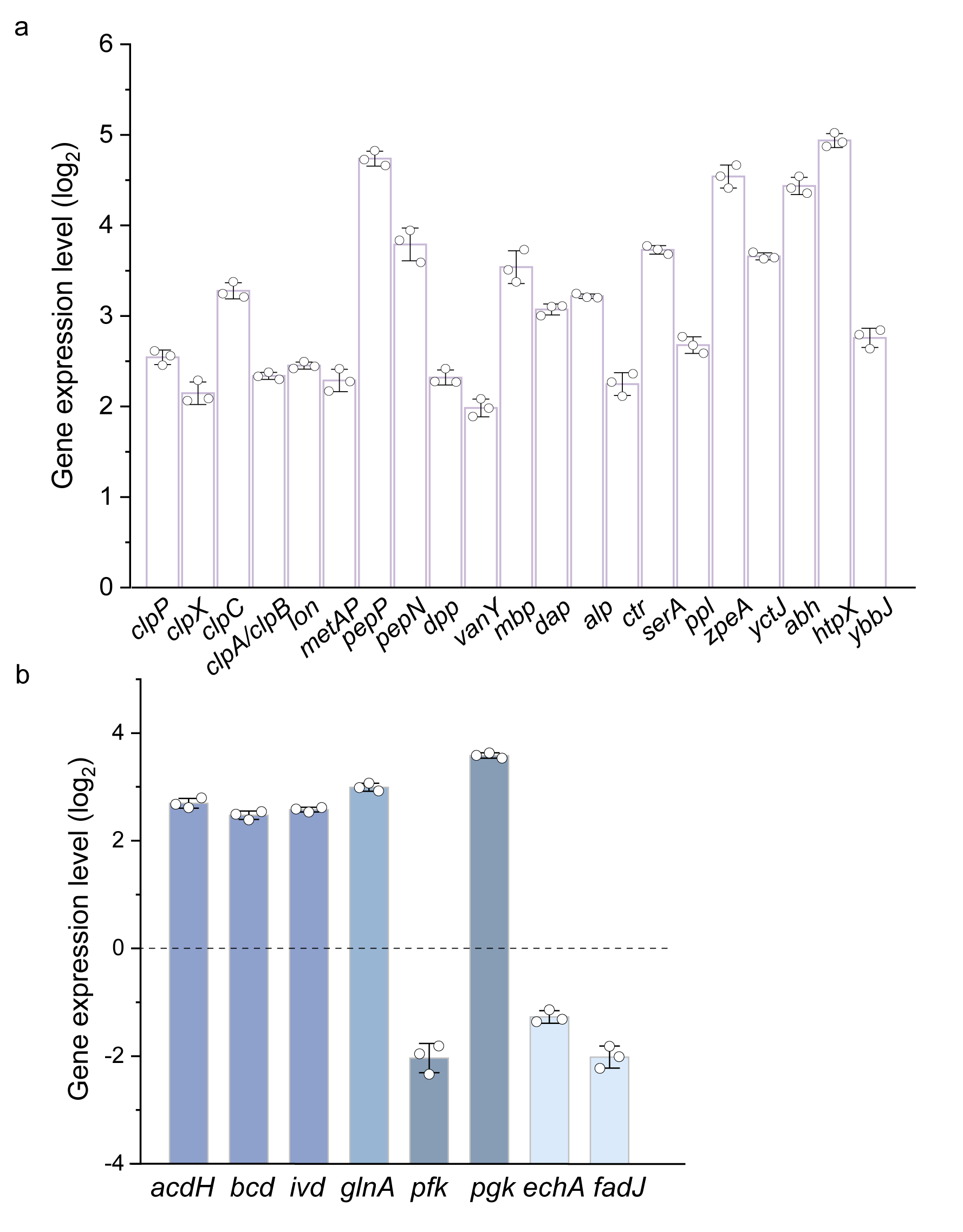


**Fig. S5** **RT–qPCR analysis of transcriptional changes in D184 relative to the wild-type *S. spinosa*.** Log2 fold changes (D184/WT) are shown for 21 protease genes and key pathway genes.


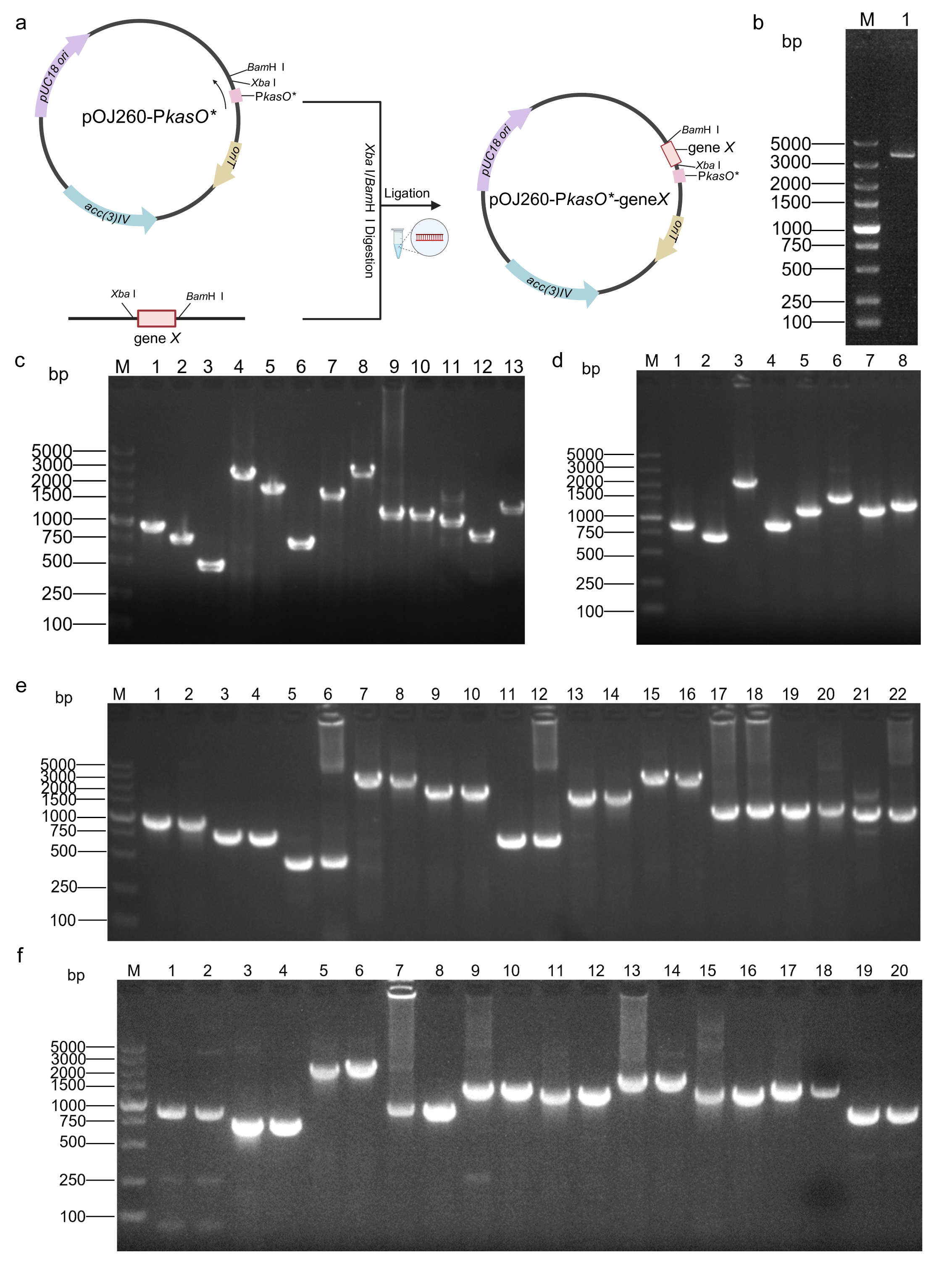


**Fig. S6 Construction and validation of overexpression vectors for 21 protease-related genes. a** Schematic representation of the overexpression vector construction. **b** Double digestion of the pOJ260-P*kasO** vector（*Bam*H Ⅰ */Xba* Ⅰ）, M: 5000DL DNA marker. **c** PCR amplification of 13 protease-related genes, M: 5000DL; 1-13: *htpX*, *clpA/clpB*, *ybbJ*, *clpC*, *dpp*, *lon*, *ytcJ*, *pepN*, *vanY*, *pepP*, *mbp*, *ctr*, *zpeA* fragment. **d** PCR amplification of 8 protease-related genes, M: 5000DL;1-8: *dap*, *clpP*, *ppl*, *metAP*, *clpX*, *alp*, *abh*, *serA* fragment. **e** PCR validation of recombinant plasmids for the overexpressed genes, M: 5000DL; 1-2: pOJ260-P*kasO**-*htpX*;3-4: pOJ260-P*kasO**-*clpA/clpB*; 5-6: pOJ260-P*kasO**-*ybbJ*; 7-8: pOJ260-P*kasO**-*clpC*; 9-10: pOJ260-P*kasO**-*dpp*; 11-12: pOJ260-P*kasO**-*lon*; 13-14: pOJ260-P*kasO**-*ytcJ*; 15-16: pOJ260-P*kasO**-*pepN*; 17-18: pOJ260-P*kasO**-*vanY*; 19-20: pOJ260-P*kasO**-*pepP*; 21-22: pOJ260-P*kasO**-*mbp.* **f** PCR validation of recombinant plasmids for the overexpressed genes, M: 5000DL; 1-2: pOJ260-P*kasO**-*dap*; 3-4: pOJ260-P*kasO**-*clpP*; 5-6: pOJ260-P*kasO**-*ppl*; 7-8: pOJ260-P*kasO**-*metAP*; 9-10: pOJ260-P*kasO**-*clpX*; 11-12: pOJ260-P*kasO**-*alp*; 13-14: pOJ260-P*kasO**-*abh*; 15-16: pOJ260-P*kasO**-*serA*; 17-18: pOJ260-P*kasO**-*zpeA*; 19-20: pOJ260-P*kasO**-*ctr.*


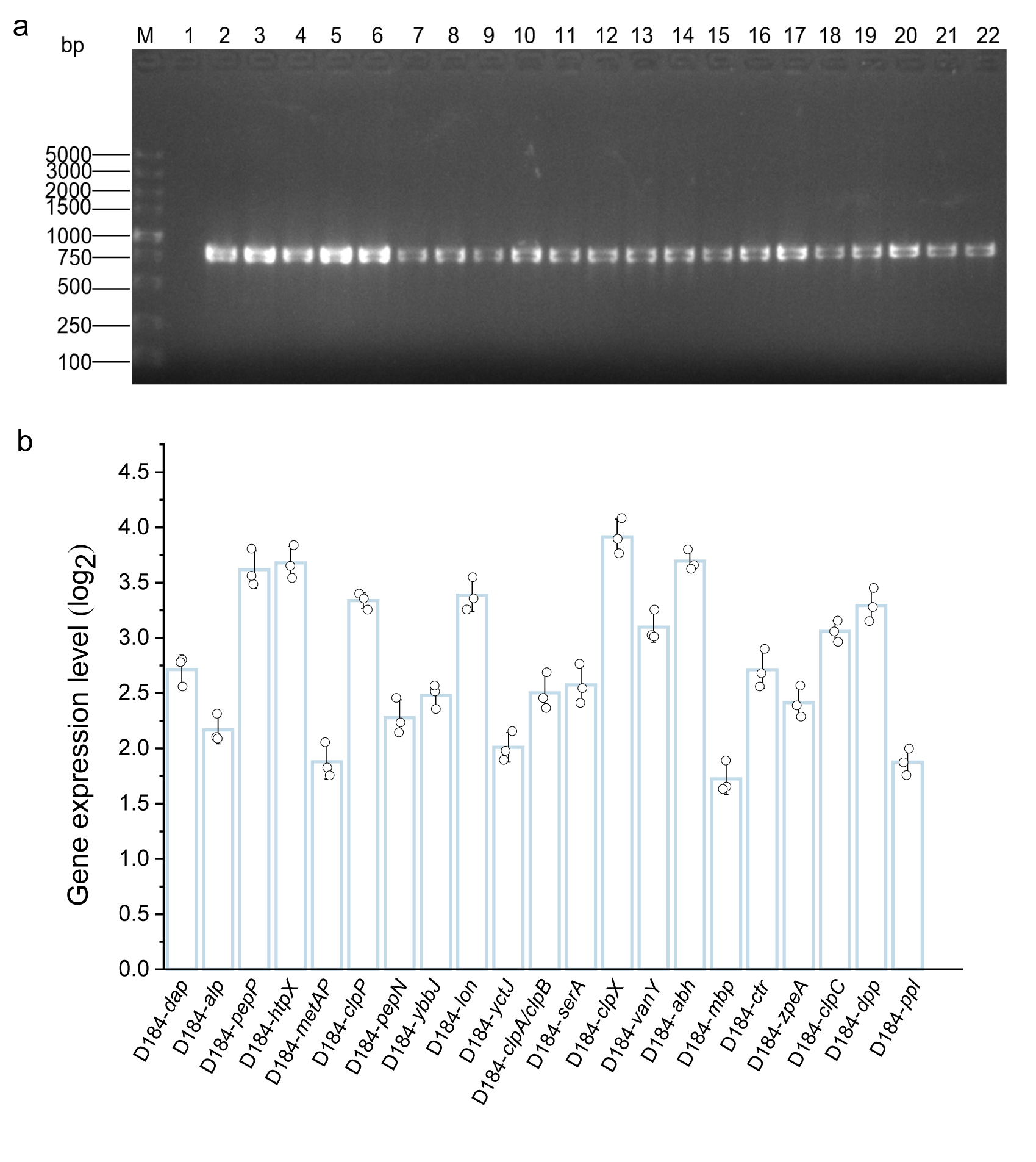


**Fig. S7** **Construction of 21 protease overexpression engineered strains. a** PCR verification of the *apr* gene in 21 overexpression engineered strains, M: 5000DL DNA marker. **b** Expression levels of the relevant genes in the 21 protease overexpression strains on day 4, by RT-qPCR. M: 5000DL; 1: control D184; 2: D184-*htpX*; 3: D184- *clpA/clpB*; 4: D184-*ybbJ*; 5: D184-*clpC*; 6: D184-*dpp*; 7: D184-*lon*; 8: D184-*ytcJ*; 9: D184-*pepN*; 10: D184-*vanY*; 11: D184-*pepP*; 12: D184-*mbp*;13: D184-*dap*; 14: D184-*clpP*; 15: D184-*ppl*; 16: D184-*metAP*; 17: D184-*clpX*; 18: D184-*alp*; 19: D184-*abh*; 20: D184-*serA*; 21: D184-*zpeA*; 22: D184-*ctr.*





**Fig. S8 Spinosad titer of eight protease-overexpressing engineered strains showing decreased production or no significant difference.** Multiple comparison significance was tested to **P* < 0.05, ***P* < 0.01, ****P* < 0.001 by one-way ANOVA followed by Dunnett’s post-hoc test.


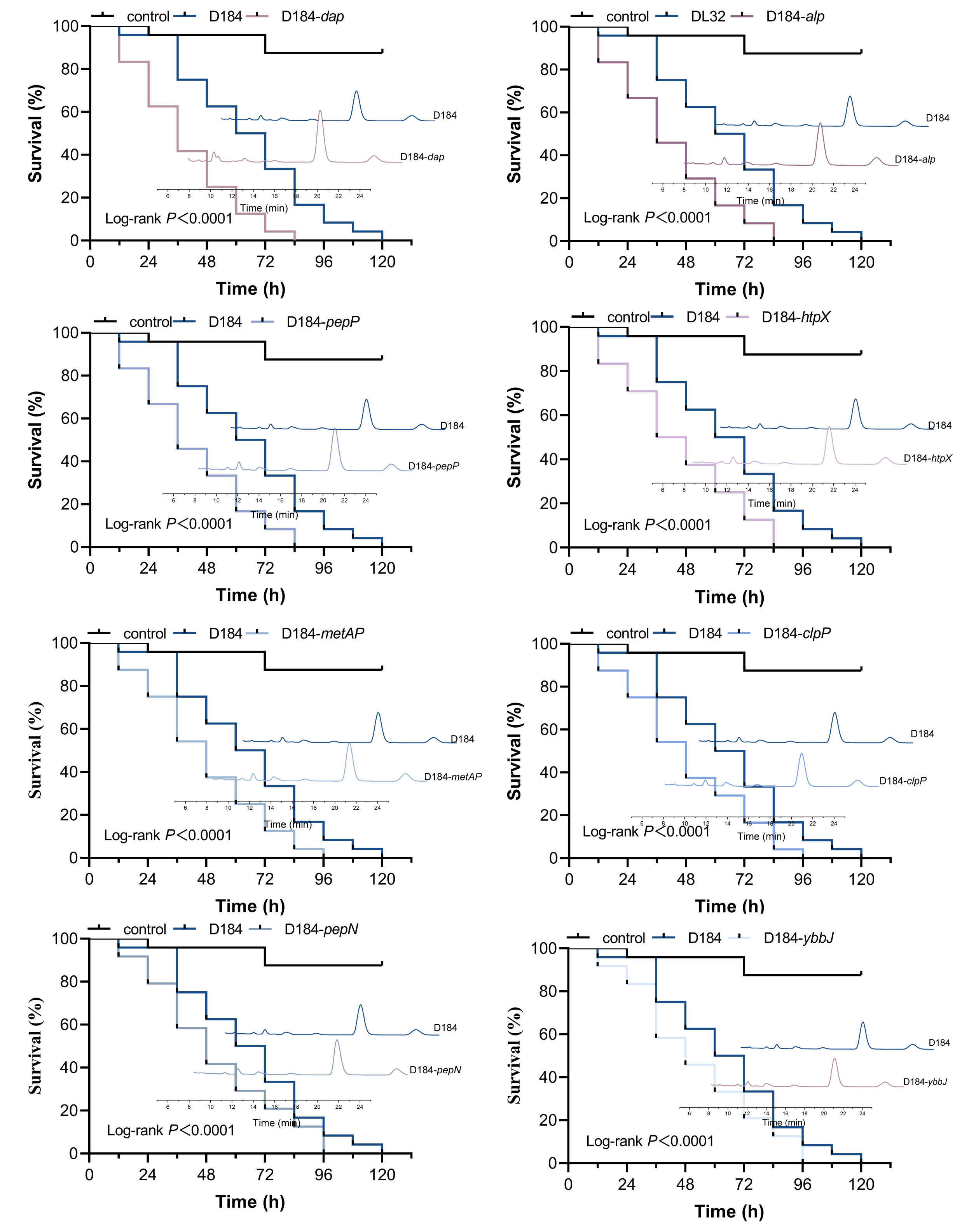


**Fig. S9 Insecticidal activity of eight Protease-overexpressing engineered strains against *Helicoverpa armigera* larvae.** From left to right: D184-*dap*, D184-*alp*, D184-*pepP*, D184-*htpX*, D184-*metAP*, D184-*clpP*, D184- *pepN*, and D184-*ybbJ*. Survival rate (%) was analyzed using the log-rank test (*P* < 0.05).


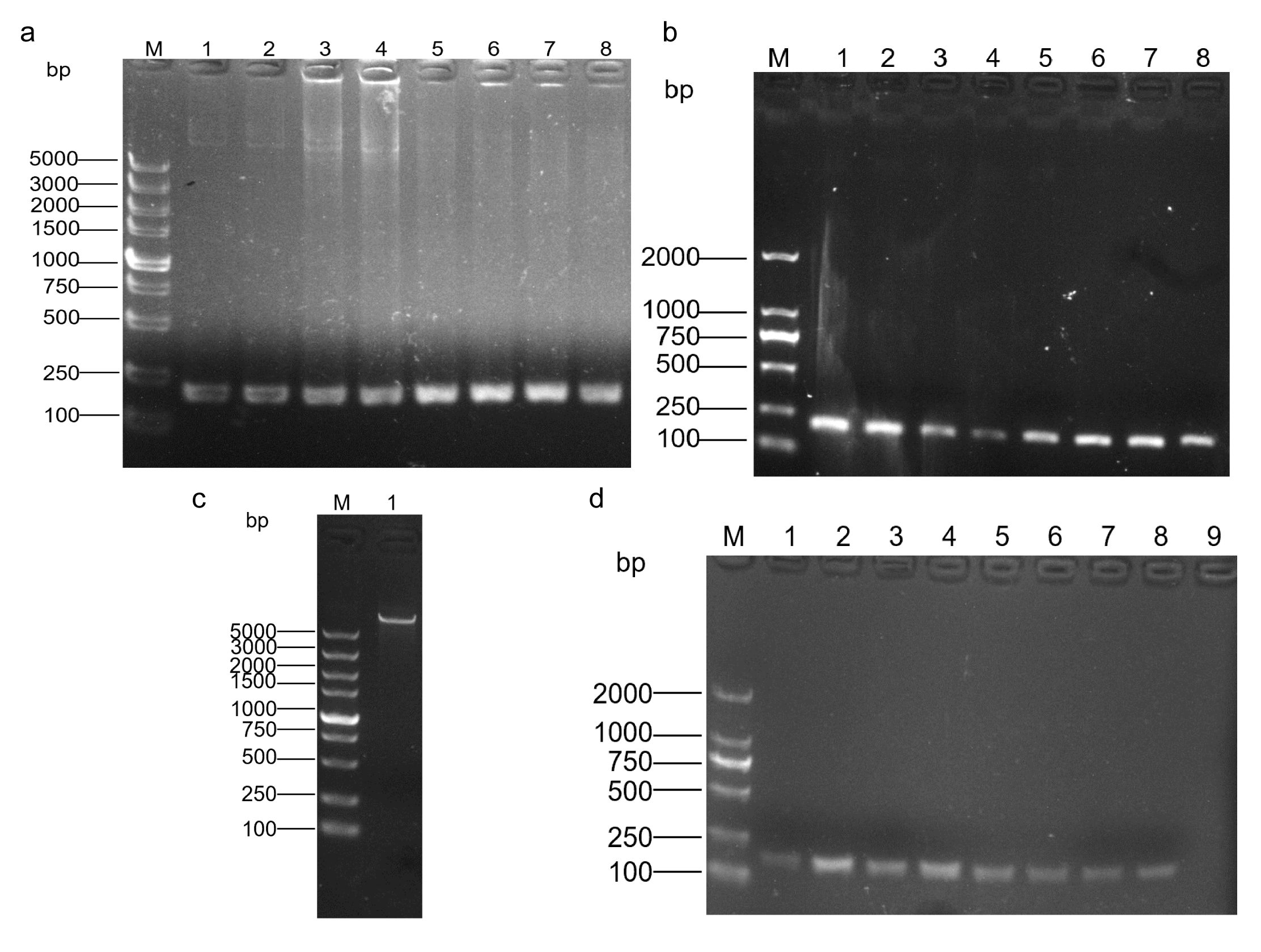


**Fig. S10 Construction of the knockdown expression vectors. a** PCR amplification of sgRNA targeting the *dap*, *alp*, *pepP*, *htpX*, *metAP*, *clpP*, *pepN*, *ybbJ*, M:5000DL; 1: sgRNA*_dap_*; 2: sgRNA*_alp_*; 3: sgRNA*_pepP_*; 4: sgRNA*_htpX_*; 5: sgRNA*_metAP_*; 6: sgRNA*_clpP_*; 7: sgRNA*_pepN_*; 8: sgRNA*_ybbJ_* .**b** Double digestion and purification of sgRNAs using *Xba* I/*Eco*R I, M:5000DL; 1: sgRNA*_dap_*; 2: sgRNA*_alp_*; 3: sgRNA*_pepP_*; 4: sgRNA*_htpX_*; 5: sgRNA*_metAP_*; 6: sgRNA*_clpP_*; 7: sgRNA*_pepN_*; 8: sgRNA*_ybbJ_*. **c** Double digestion of pSET-*dCas9* plasmid（*Xba* I /*EcoR* I）. **d** PCR validation of sgRNA in the repression expression vectors. M:2000DL; 1: pSET-*dCas9-dap*; 2: pSET-*dCas9- alp*; 3: pSET-*dCas9-pepP*; 4: pSET-*dCas9-htpX*; 5: pSET-*dCas9-metAP*; 6: pSET-*dCas9-clpP*; 7: pSET-*dCas9-pepN*; 8: pSET-*dCas9*-*ybbJ*; 9: control, pSET-*dCas9-only.*


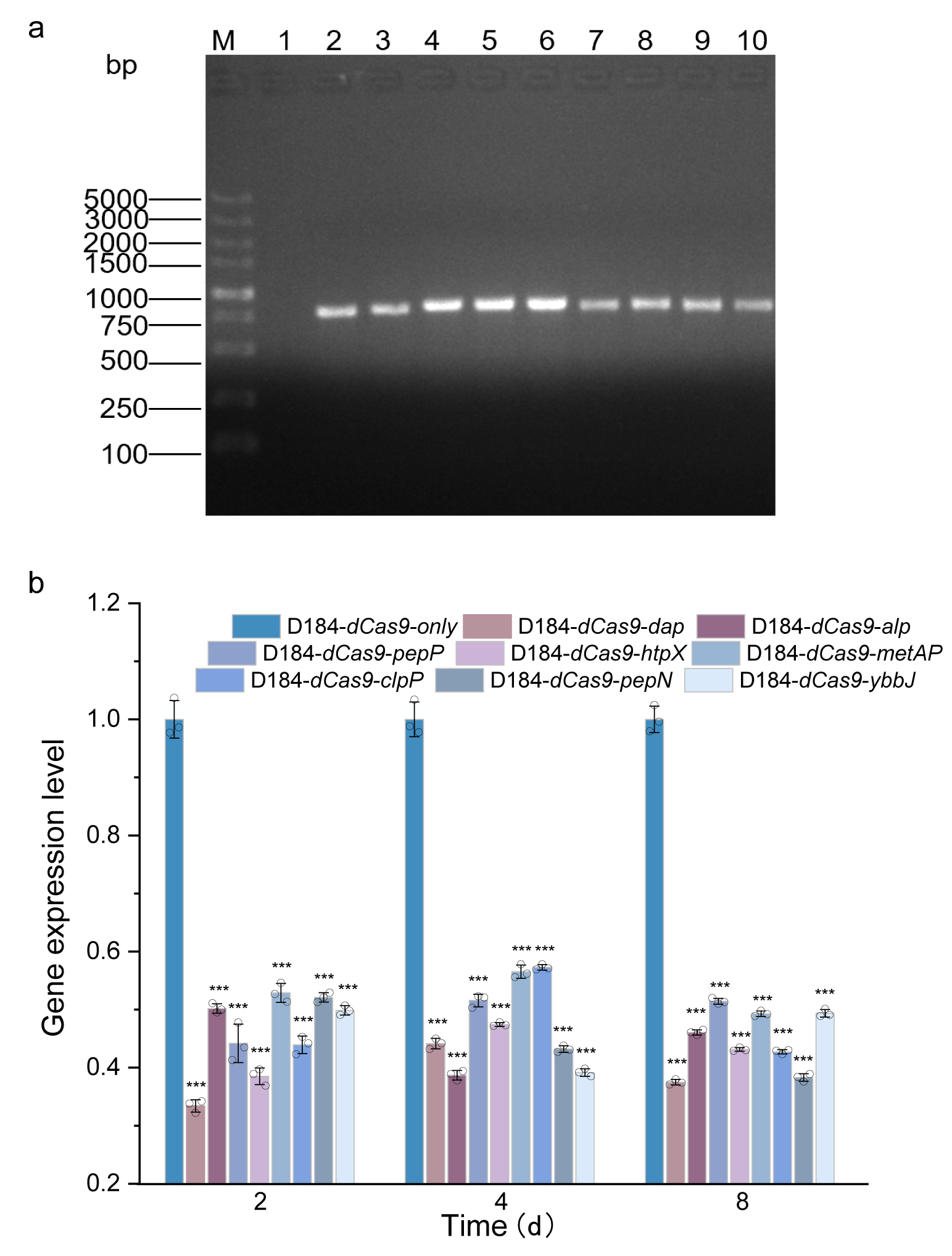


**Fig. S11 Construction of knockdown expression engineered strains. a** PCR validation of the apr gene in knockdown expression engineered strains, M:5000DL;1: D184; 2: D184*-dCas9-only*; 3: D184*-dCas9-dap*; 4: D184*-dCas9-alp*; 5: D184*-dCas9-pepP*; 6: D184*-dCas9-htpX*; 7: D184*-dCas9-metAP*; 8: D184*-dCas9-clpP*; 9: D184*-dCas9-pepN*; 10: D184*-dCas9-ybbJ*. **b** Transcriptional levels of *dap*, *alp*, *pepP*, *htpX*, *metAP*, *clpP*, *pepN*, *ybbJ* at different time points, by RT-qPCR. Multiple comparison significance was tested to **P* < 0.05, ***P* < 0.01, ****P* < 0.001 by one-way ANOVA.


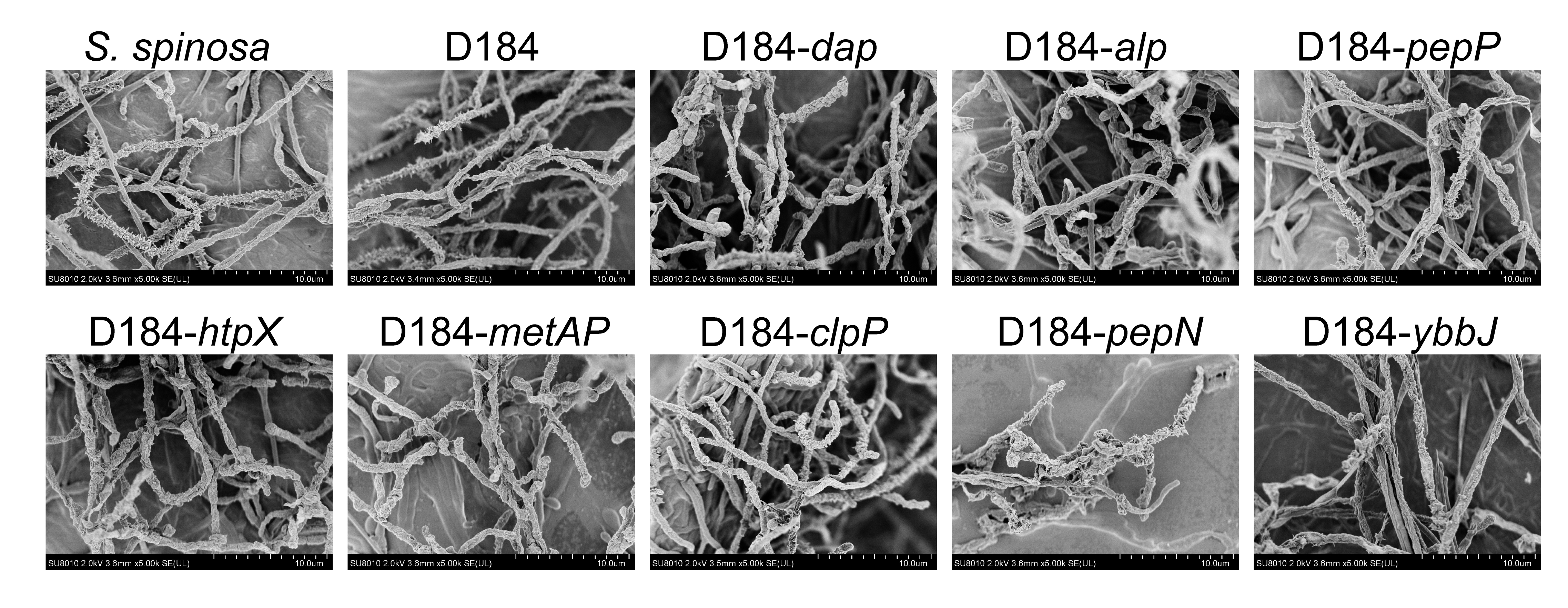


**Fig. S12 Scanning electron micrograph (SEM) of the strain at a magnification of 5.00k.**


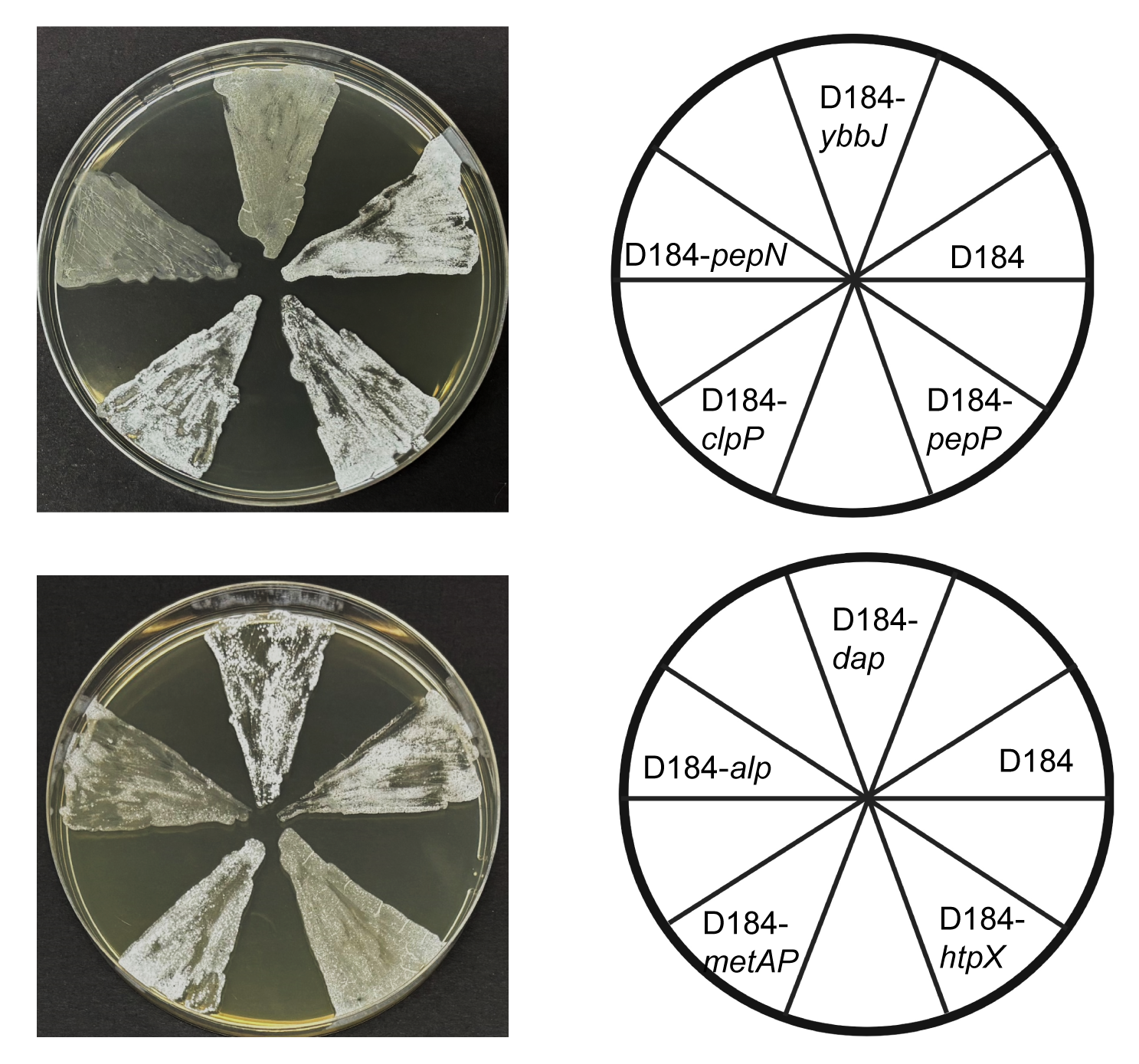


**Fig. S13 Observation of spore morphology.** The spores were cultured on TSB agar medium, and their morphological characteristics were examined at day 4 of cultivation.

**Fig. S14 RT-qPCR transcriptional analysis of six key spore differentiation-related genes, including *ssgA*, *whiA*, *whiB*, *bldD*, *wblE*, and *sigF*.** Statistical significance determined using the one-way ANOVA followed by Dunnett’s post-hoc test (n = 3). **P* < 0.05, ***P* < 0.01, ****P* < 0.001.


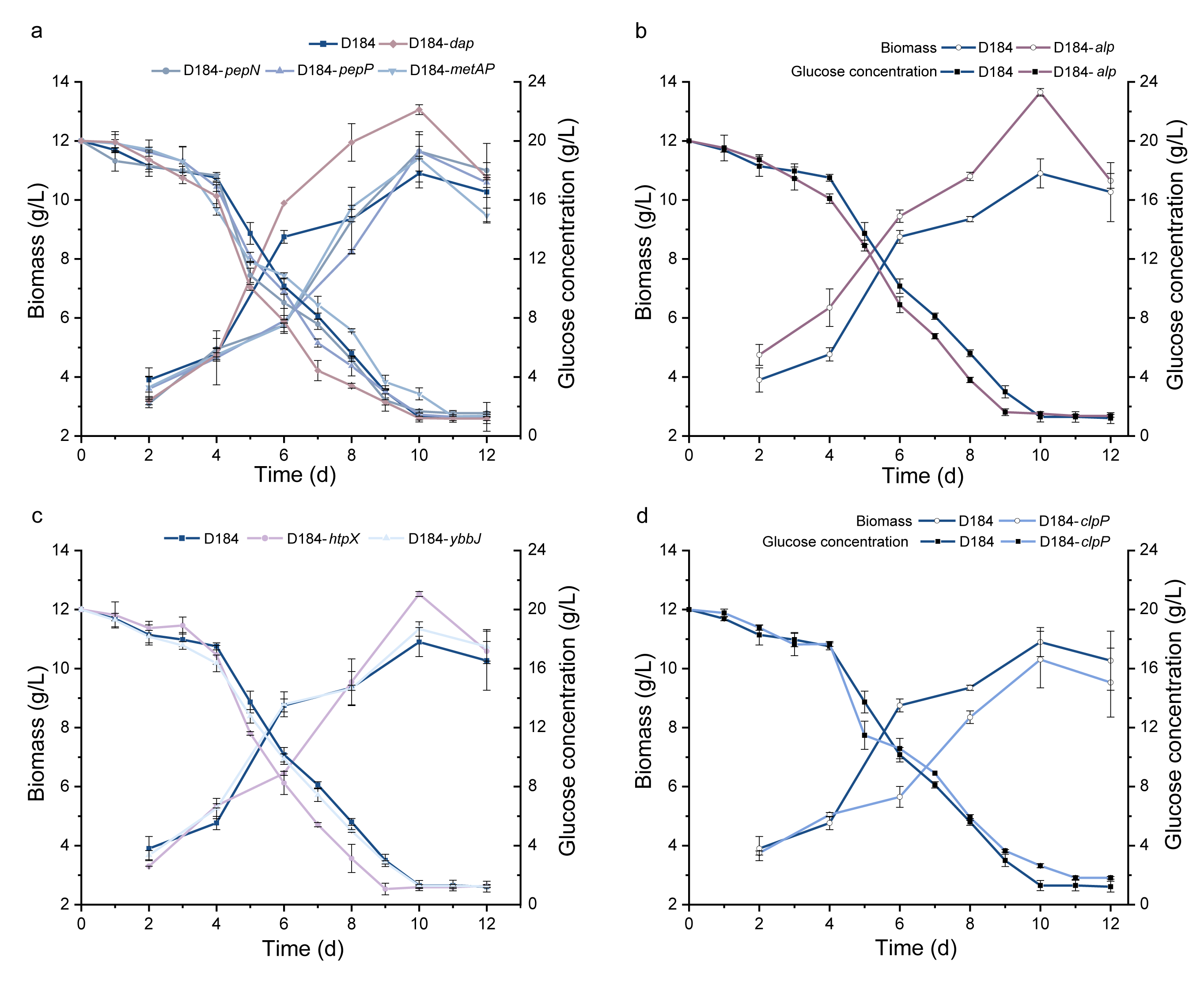


**Fig. S15 Accumulation curves of biomass and glucose consumption** **in 8 protease overexpression engineered strains that exhibit an increase in spinosad biosynthesis.**


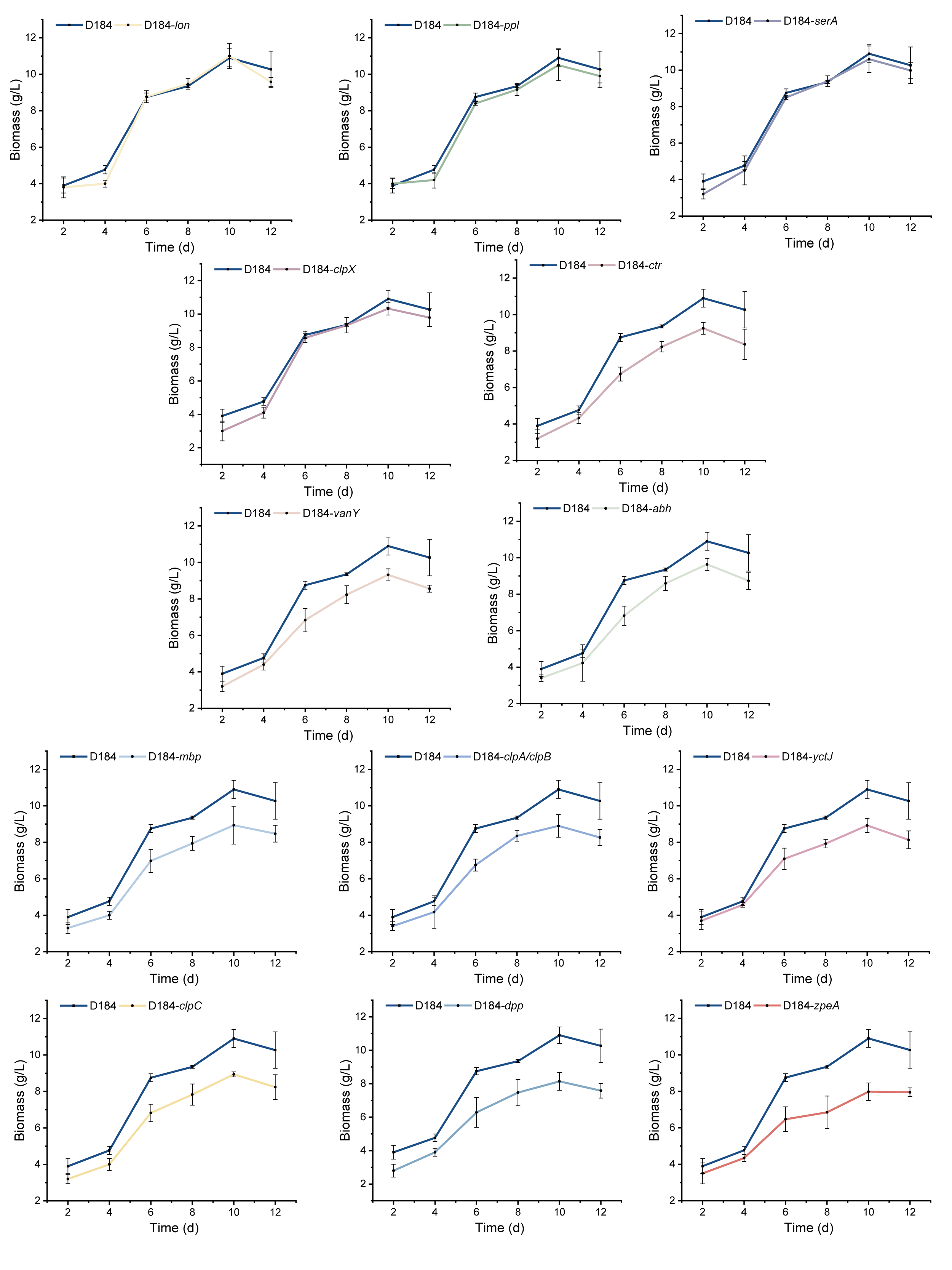


**Fig. S16 Biomass accumulation in 13 protease overexpression engineered strains that did not exhibit an increase in spinosad biosynthesis.**


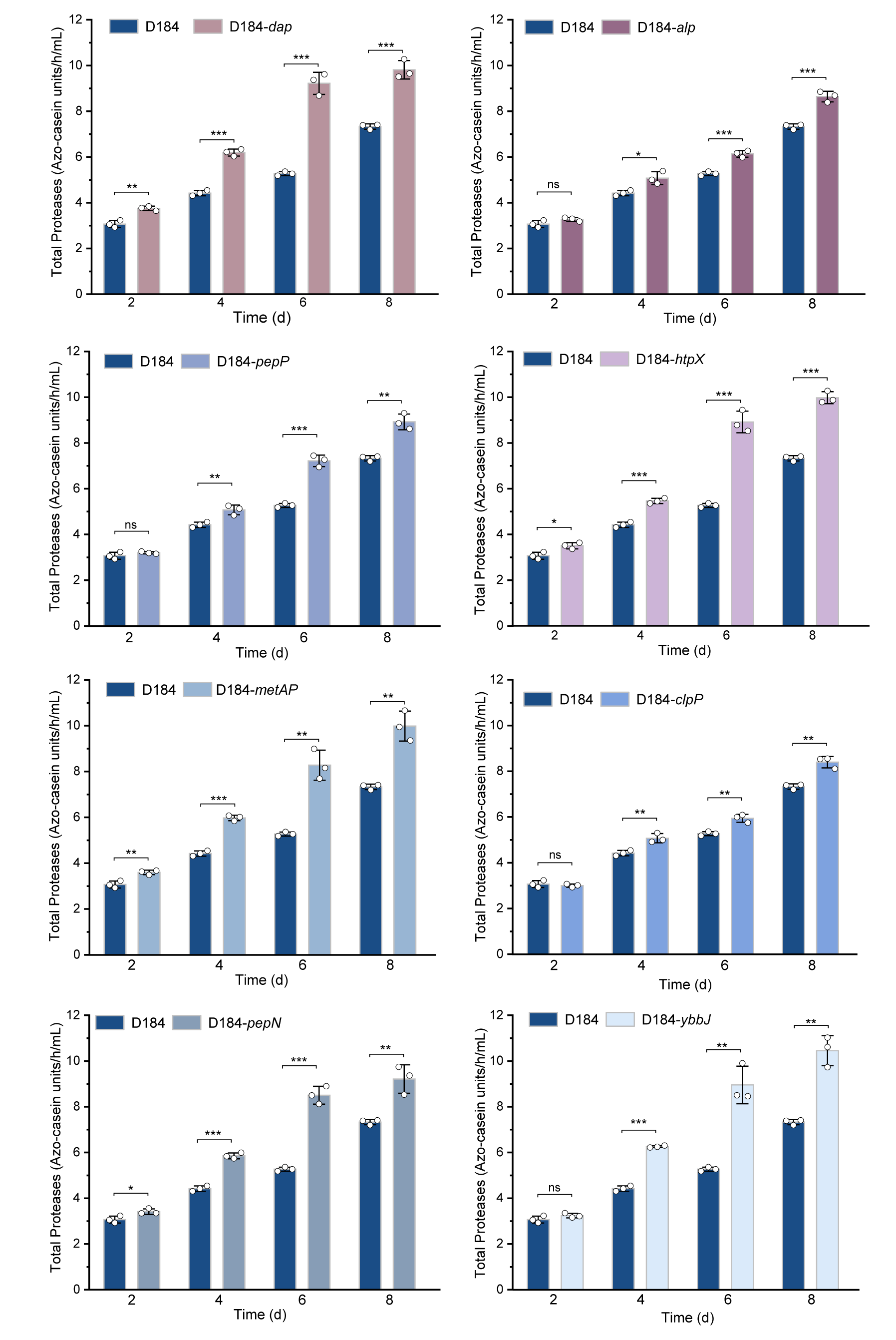


**Fig. S17 Total extracellular protease activity in the D184 and eight overexpressing engineered strains during fermentation on days 2, 4, 6, and 8.** Statistical significance determined using the *t*-test (n = 3). **P* < 0.05, ***P* < 0.01, ****P* < 0.001.


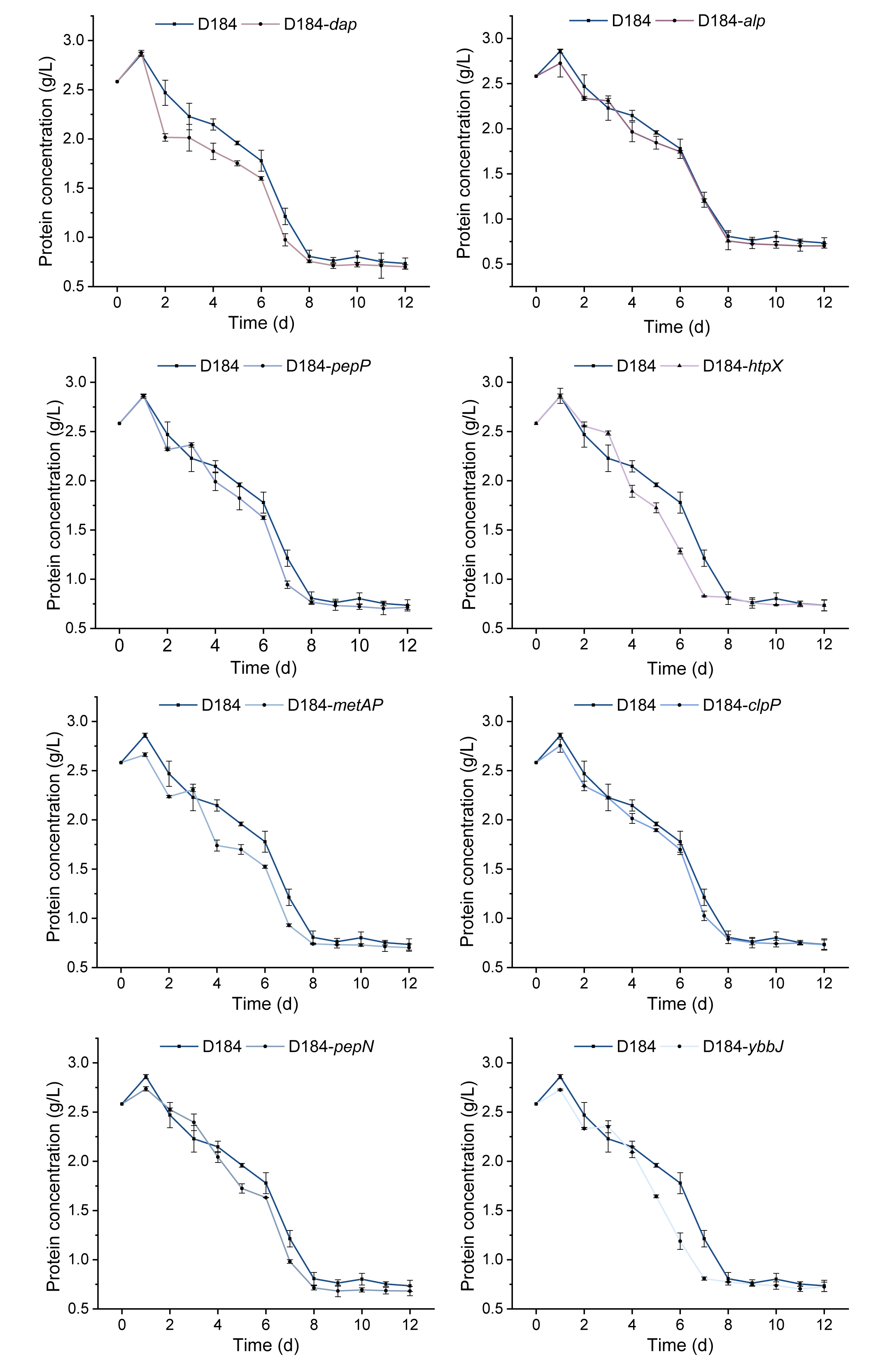


**Fig. S18 Detection of protein content in the fermentation supernatant of D184 and eight overexpressing engineered strains.** The data are presented as mean ± SD (n = 3 biological replicates).


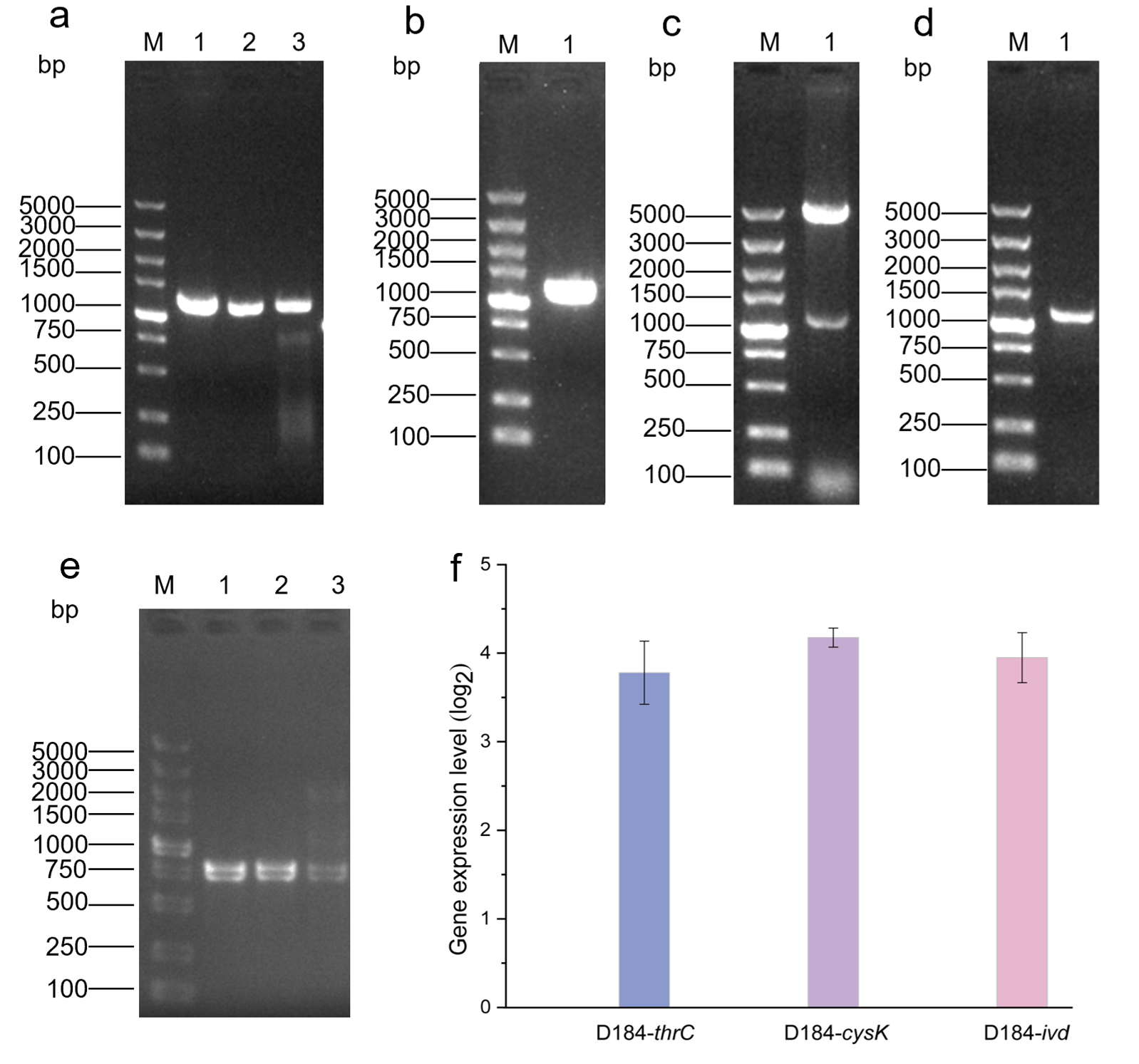


**Fig. S19 Construction of D184-*thrC,* D184-*cysK,* D184-*ivd* engineered strains. a** PCR amplification of the *thrC*, *cysK*, *and ivd* fragments, M: 5000DL; 1: *thrC* fragment; 2: *cysK* fragment; 3: *ivd* fragment. **b** PCR verification of *thrC* in the pOJ260-P*kasO**-*thrC* construct, M: 5000DL. **c** Double digestion verification of the pOJ260-P*kasO**-*cysK* construct（*Bam*H Ⅰ */Xba* Ⅰ）M: 5000DL.

**d** PCR verification of *ivd* in the pOJ260-P*kasO**-*ivd* construct, M: 5000DL. **e** PCR verification of the *apr* gene in D184-*thrC*, D184-*cysK*, and D184-*ivd* engineered strains. **f** Expression levels of the relevant genes in theD184-*thrC*, D184-*cysK*, D184-*ivd* on day 4, by RT-qPCR.


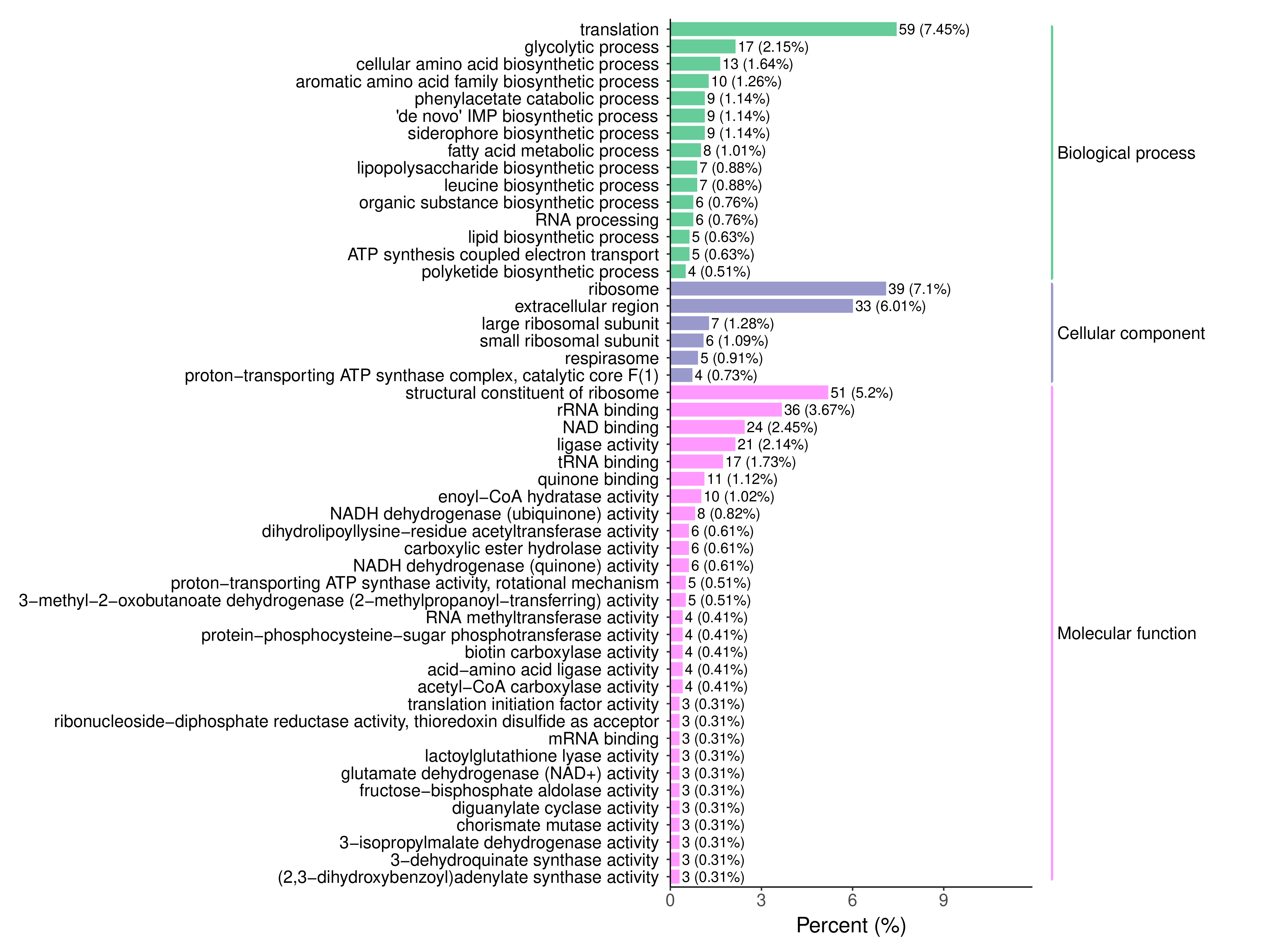


**Fig. S20 GO enrichment analysis of differentially expressed proteins**. The x-axis represents the proportion of differentially expressed proteins annotated to each GO term, while the y-axis lists the names of the GO terms. The numbers within the bars indicate the count of differentially expressed proteins annotated to each GO term, with values in parentheses showing the ratio of annotated differentially expressed proteins to the total number of annotated proteins. The labels on the far right indicate the primary GO classification for each term.





**Fig. S21 Transcriptional response to carbon source utilization.** On day 4, RT-qPCR analysis was performed to assess the expression of four key genes involved in glucose uptake and glycolysis, including *ptsI*, *crr*, *pfk*, and *pyk*, in D184 and D184*-dap* strains. Statistical significance determined using the *t*-test (n = 3). **P* < 0.05, ***P* < 0.01, ****P* < 0.001.


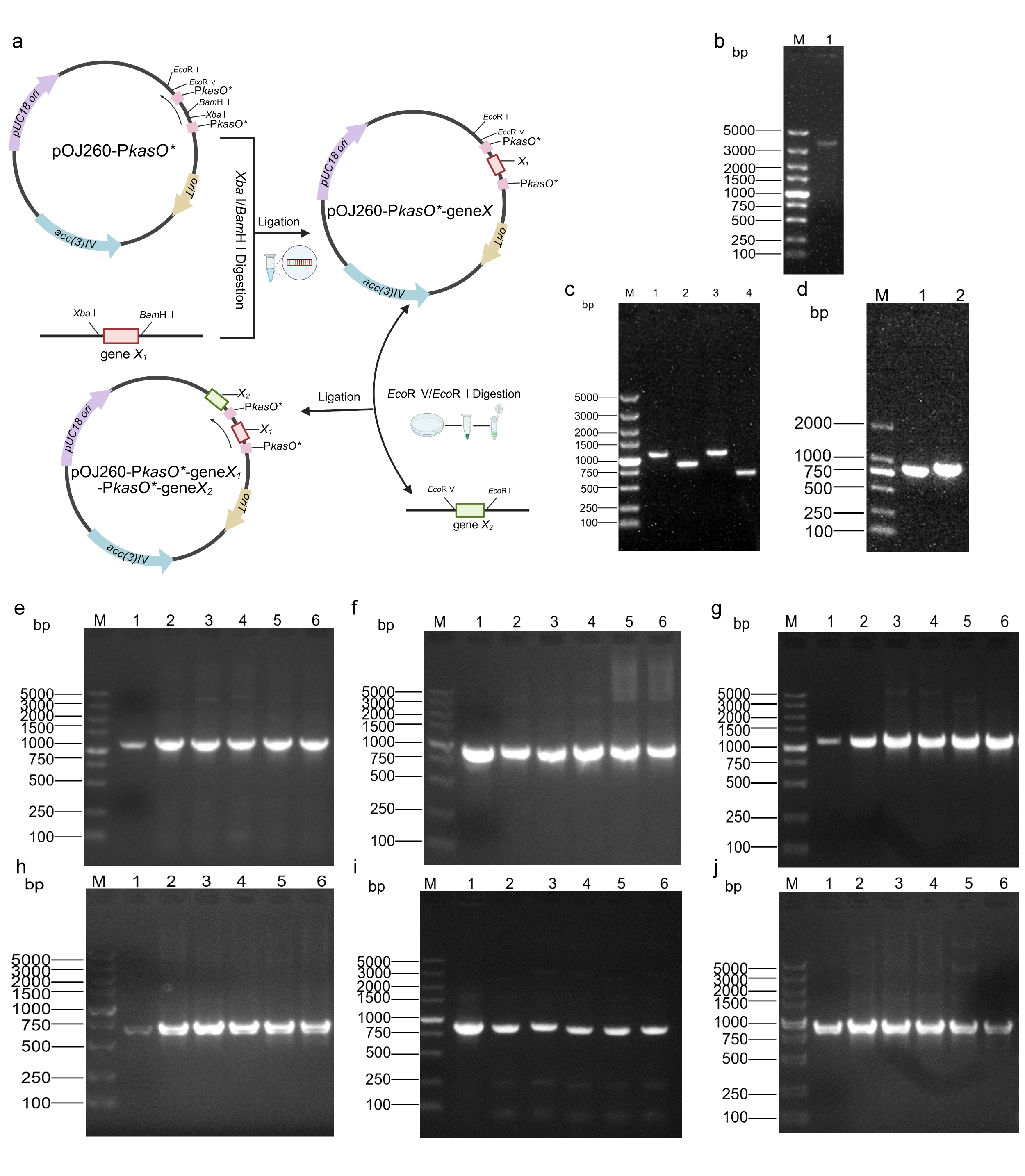


**Fig. S22 Construction of co-expression vectors. a** Schematic diagram illustrating the construction process of the pOJ260-P*kasO**vector.**b** pOJ260-P*kasO**vector **c** PCR amplification of genes， M:5000DL;1-4: *pepP*, *metAP*, *alp*, *clpP*. **d** PCR amplification of genes,1-2: *dap*, *htpX*. **e** PCR verification of *pepP* in the co-expression vector, M:5000DL;1: control; 2: pOJ260-P*kasO**-*dap*-P*kasO**-*pepP*;3: pOJ260-P*kasO**-*alp*-P*kasO**-*pepP*;4: pOJ260-P*kasO**-*pepP*-P*kasO**-*htpX*;5: pOJ260-P*kasO**-*pepP*-P*kasO**-*metAP*; 6: pOJ260-P*kasO**-*pepP*-P*kasO**-*clpP*. **f** PCR verification of *metAP* in the co-expression vector, M:5000DL;1: control; 2: pOJ260-P*kasO**-*dap*-P*kasO**-*metAP*;3: pOJ260-P*kasO**-*alp*-P*kasO**-*metAP*;4: pOJ260-P*kasO**-*pepP*-P*kasO**-*metAP*;5: pOJ260-P*kasO**-*htpX*-P*kasO**-*metAP*; 6: pOJ260-P*kasO**-*clpP*-P*kasO**-*metAP*. **g** PCR verification of *alp* in the co-expression vector, M:5000DL;1: control; 2: pOJ260-P*kasO**-*alp*-P*kasO**-*pepP*;3: pOJ260-P*kasO**-*alp*-P*kasO**-*metAP*;4: pOJ260-P*kasO**-*alp*-P*kasO**-*clpP*;5: pOJ260-P*kasO**-*dap*-P*kasO**-*alp*; 6: pOJ260-P*kasO**-*alp*-P*kasO**-*htpX*. **h** PCR verification of *clpP* in the co-expression vector, M:5000DL;1: control M:5000DL;1: control; 2: pOJ260-P*kasO**-*dap*-P*kasO**-*clpP*;3: pOJ260-P*kasO**-*alp*-P*kasO**-*clpP*;4: pOJ260-P*kasO**-*pepP*-P*kasO**-*clpP*;5: pOJ260-P*kasO**-*htpX*-P*kasO**-*clpP*; 6: pOJ260-P*kasO**-*clpP*-P*kasO**-*metAP*. **i** PCR verification of *dap* in the co-expression vector, M:5000DL; 1: control; 2: pOJ260-P*kasO**-*dap*-P*kasO**-*pepP*;3: pOJ260-P*kasO**-*dap*-P*kasO**-*metAP*; 4: pOJ260-P*kasO**-*dap*-P*kasO**-*alp*;5: pOJ260-P*kasO**-*dap*-P*kasO**-*clpP*; 6: pOJ260-P*kasO**-*dap*-P*kasO**-*htpX*. **j** PCR verification of *htpX* in the co-expression vector, M:5000DL;1: control; 2: pOJ260-P*kasO**-*htpX*-P*kasO**-*clpP*; 3: pOJ260-P*kasO**-*htpX*-P*kasO**-*metAP*; 4: pOJ260-P*kasO**-*alp*-P*kasO**-*htpX*; 5: pOJ260-P*kasO**-*dap*-P*kasO**-*htpX*; 6: pOJ260-P*kasO**-*pepP*-P*kasO**-*htpX*.


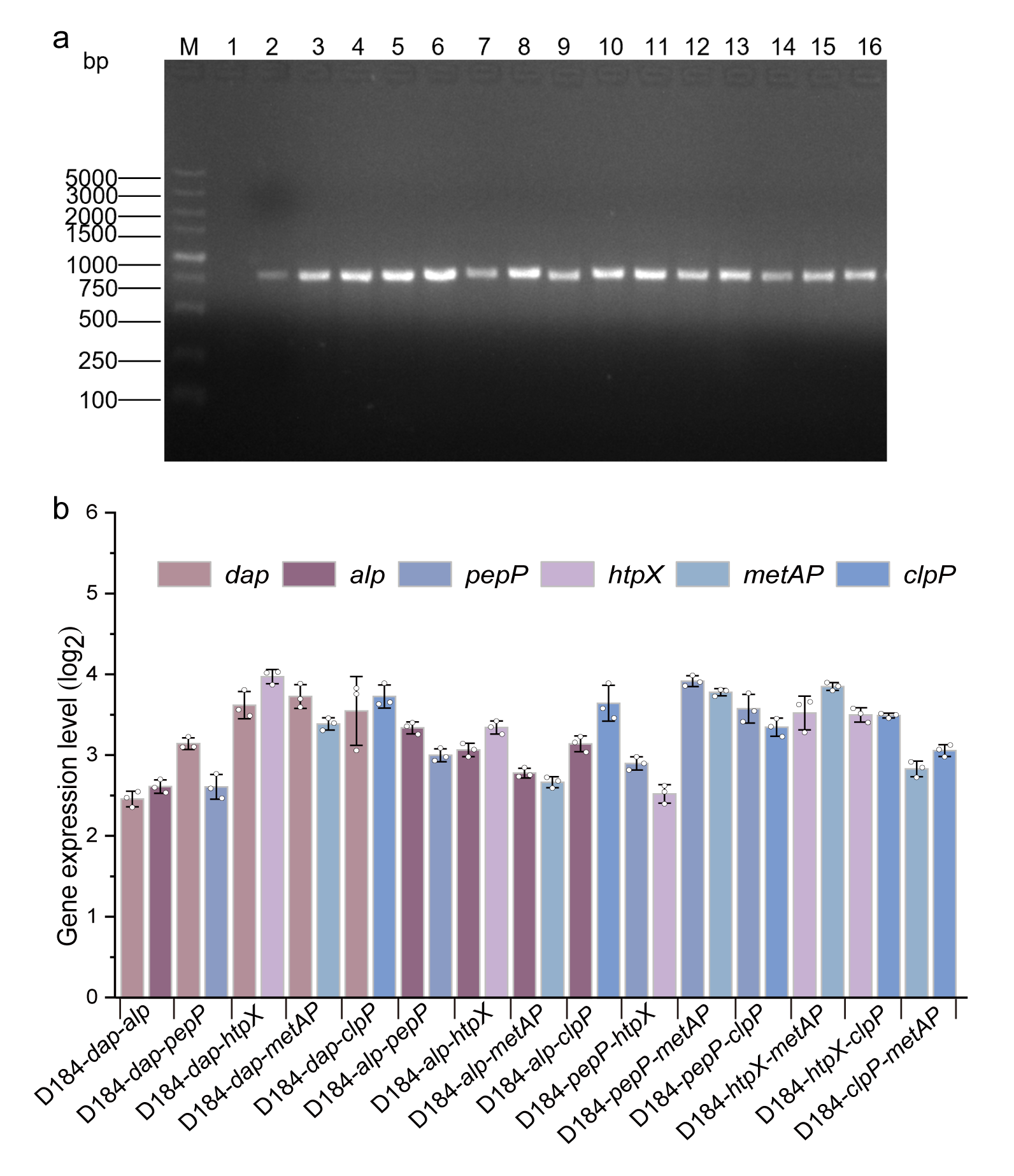


**Fig. S23** **Construction of co-expression engineered strains. a** M:5000DL; 1: D184; 2: D184-*dap*-*alp*; 3: D184-*dap*-*pepP*; 4: D184-*dap*-*htpX*; 5: D184-*dap*-*metAP*; 6: D184-*dap*-*clpP*; 7: D184-*alp*-*pepP*; 8: D184-*alp*-*htpX*; 9: D184-*alp*-*metAP*; 10: D184-*alp*-*clpP*; 11: D184-*pepP-htpX*; 12: D184-*pepP-metAP*; 13: D184-*pepP-clpP*; 14: D184-*htpX-metAP*; 15: D184-*htpX-clpP*; 16: D184-*clpP-metAP*. **b** Expression levels of the relevant genes in the 15 protease co-expression strains on day 4, by RT-qPCR.


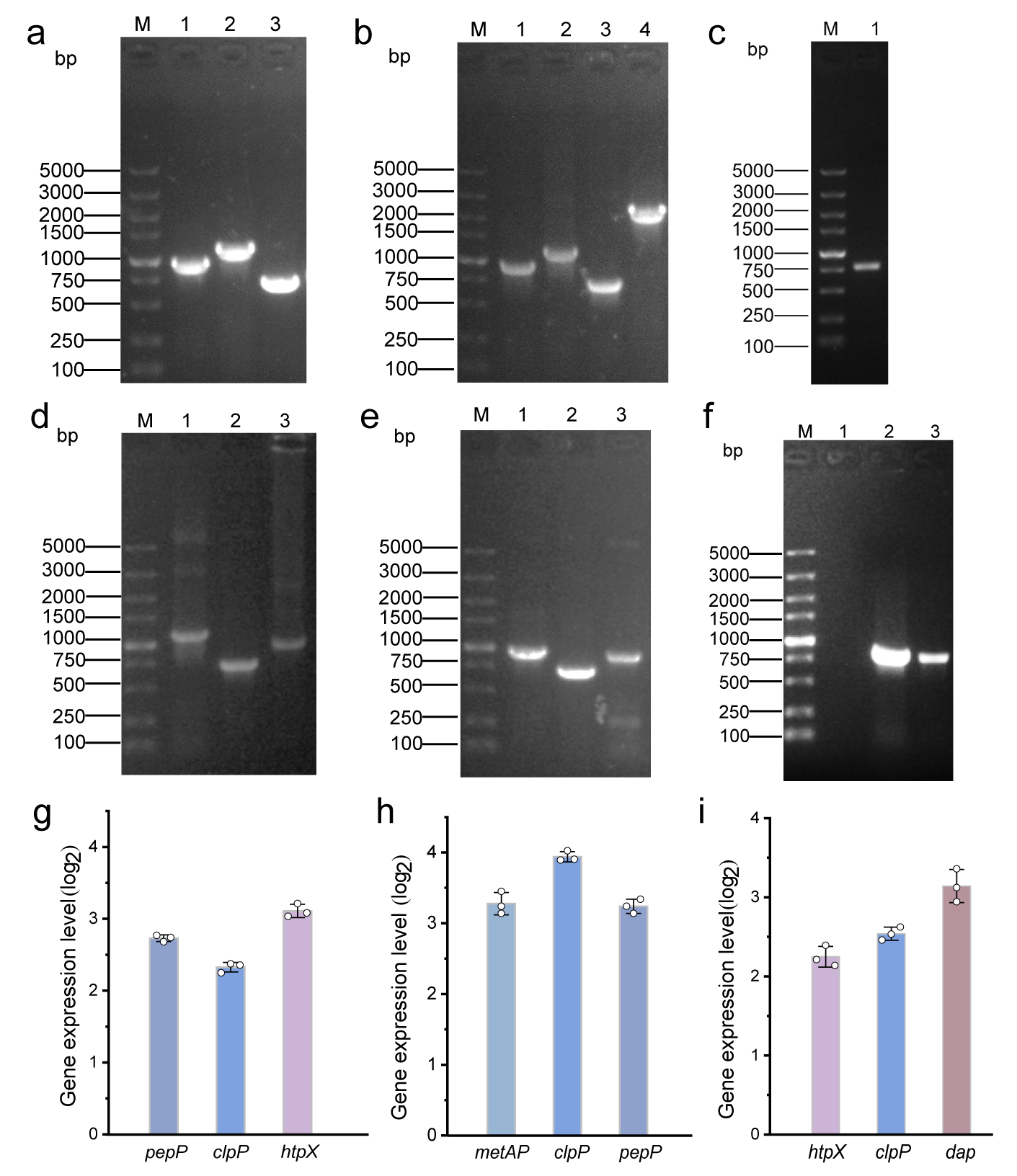


**Fig. S24 Construction of D184-*pepP*-*clpP*-*htpX*, D184-*metAP-clpP-pepP* and D184-*htpX-clpP-dap* engineered strain. a** PCR amplification of genes, M:5000DL; 1-3: *pepP*, *clpP*, *htpX.* **b** PCR verification in the pOJ260-P*kasO**-*pepP-clpP-PkasO*-htpX* construct. M: M:5000DL; 1-4: *pepP*, *clpP*, *htpX, pepP-clpP.* **c** PCR verification of the *apr* gene in the *D184-pepP-clpP-htpX.* **d** PCR verification in the pOJ260-P*kasO**-*pepP-clpP-PkasO*-metAP* construct. M: M:5000DL; 1-3: *pepP*, *clpP*, *metAP.* **e** PCR verification in the pOJ260-P*kasO**-*dap-clpP-PkasO*-htpX* construct. M: 5000DL; 1-3: *htpX, clpP, dap.* **f** PCR verification of the *apr* gene in the D184*-metAP-clpP-pepP* and D184*-htpX-clpP-dap.* **g** Transcriptional levels of *pepP, clpP,* and *htpX* in D184-*pepP*-*clpP*-*htpX.* **h** Transcriptional levels of *metAP, clpP,* and *pepP in* D184*-metAP-clpP-pepP.* **i** Transcriptional levels of *htpX, clpP,* and *dap* in D184*- htpX-clpP-dap.*


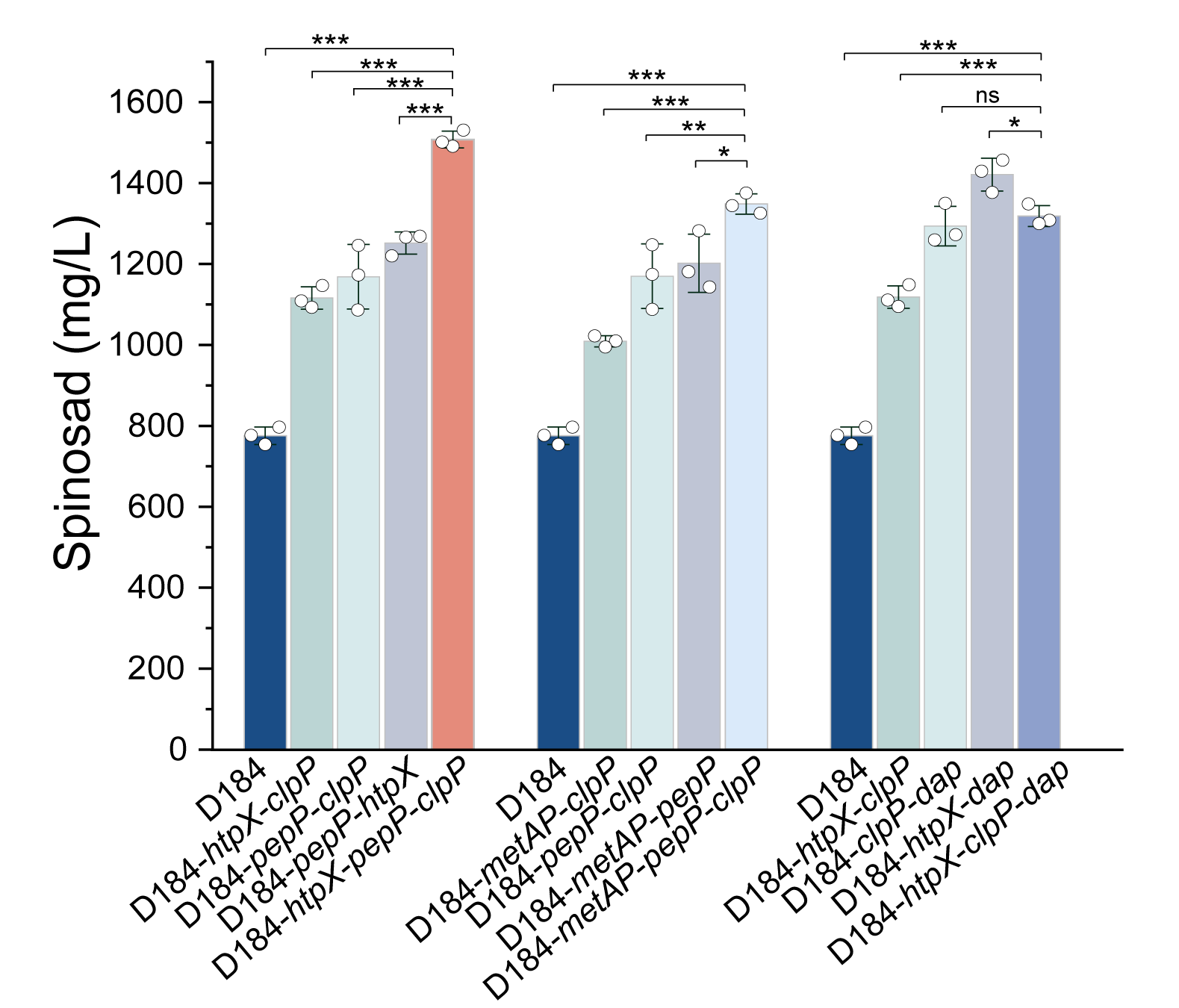


**Fig. S25 Spinosad titers of synergistic triangular gene combinations in D184.** Multiple comparison significance was tested to **P* < 0.05, ***P* < 0.01, ****P* < 0.001 by one-way ANOVA followed by Dunnett’s post-hoc test.


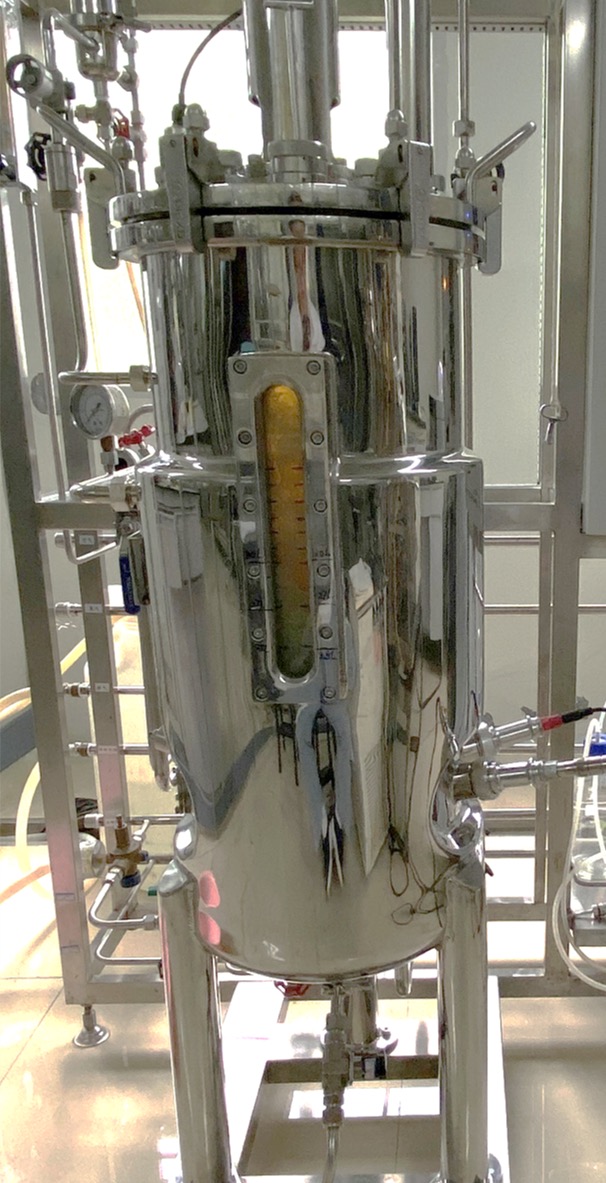


**Fig. S26** **The 50L bioreactor used in the fermentation process of *S. spinosa*.**


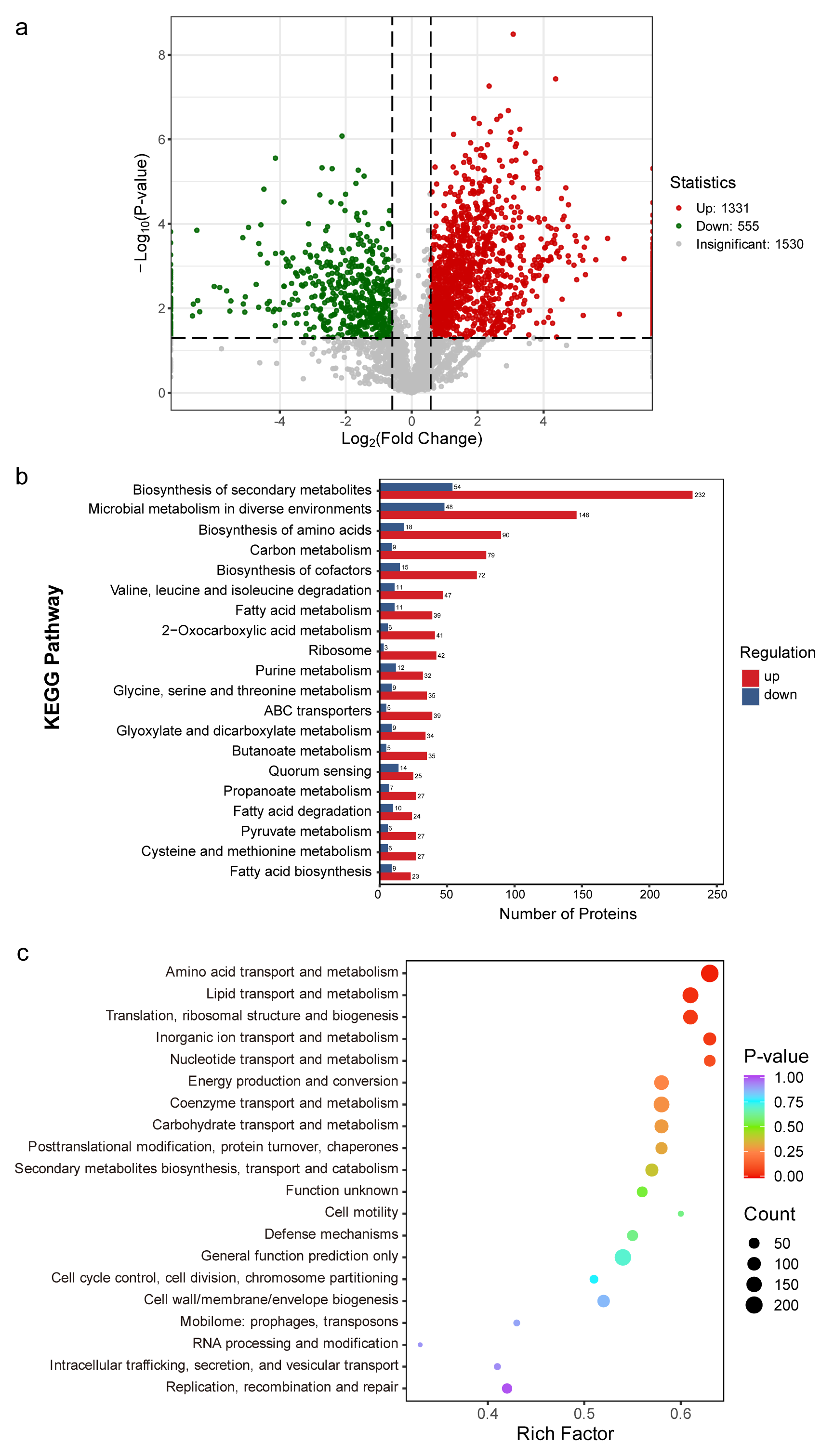


**Fig. S27** **Quantitative proteomic profiling at 96 h of D184-*pepP*-*clpP*-*htpX* relative to the parental strain D184.** **a** Volcano plot of differentially expressed proteins. The x-axis shows the log2 fold change, and the y-axis shows the -log10(P-value). Red and green dots denote significantly upregulated and downregulated differentially expressed proteins, respectively. **b** Bar chart comparing KEGG functional classifications of upregulated and downregulated proteins. The x-axis indicates the number of differentially abundant proteins assigned to each KEGG category, and the y-axis lists the KEGG functional classes. Red bars represent upregulated proteins, whereas blue bars represent downregulated proteins. **c** Bubble plot of COG enrichment analysis for differentially expressed proteins. The x-axis represents the enrichment factor,a higher enrichment factor indicates stronger enrichment. The y-axis shows COG functional descriptions. Bubble color transitions from blue to red as P values decrease, and bubble size reflects the number of differentially abundant proteins annotated to each term.


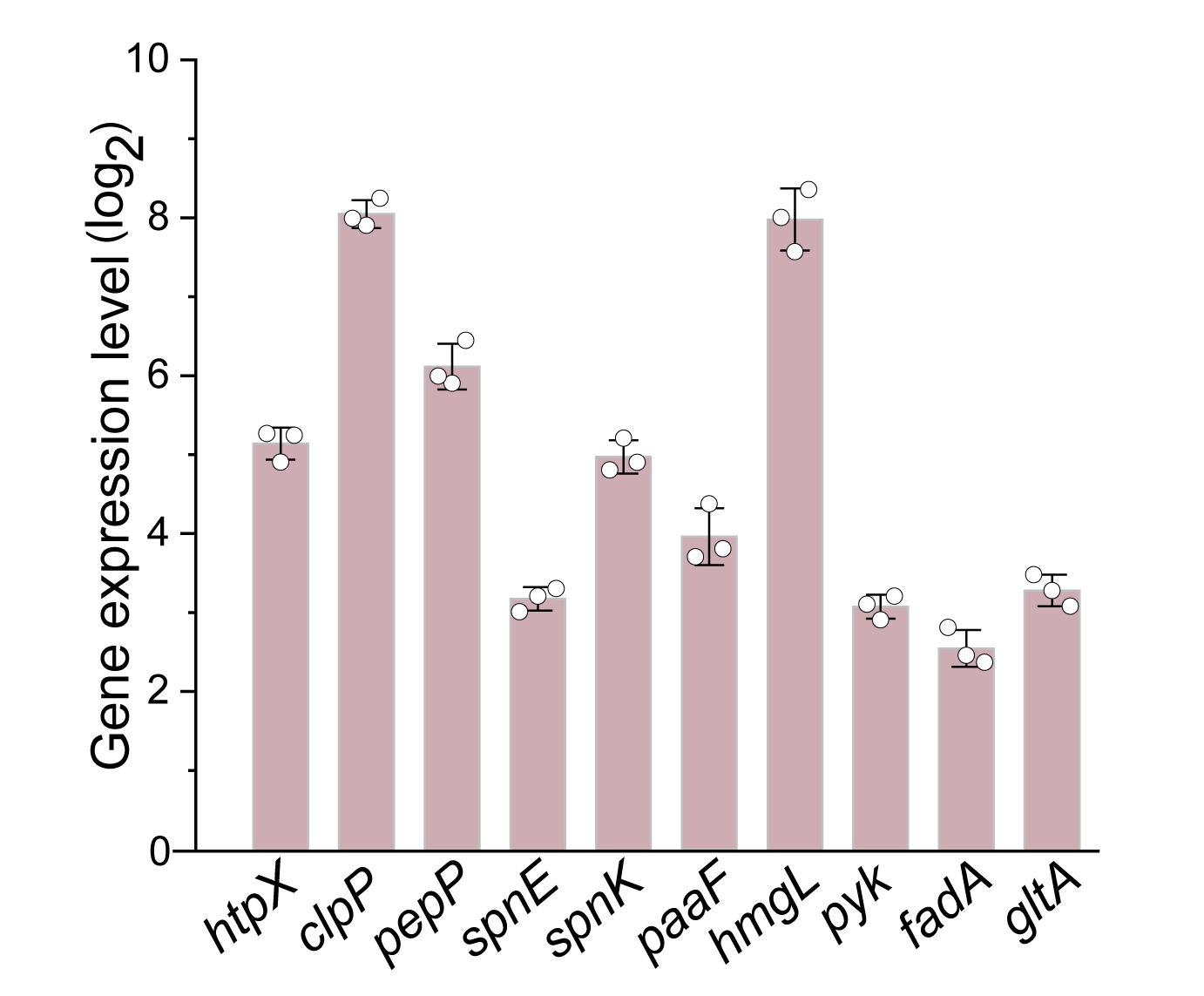


**Fig. S28 RT–qPCR validation of the transcriptional levels of selected key genes in D184-*pepP*-*clpP*-*htpX* compared with the D184.**


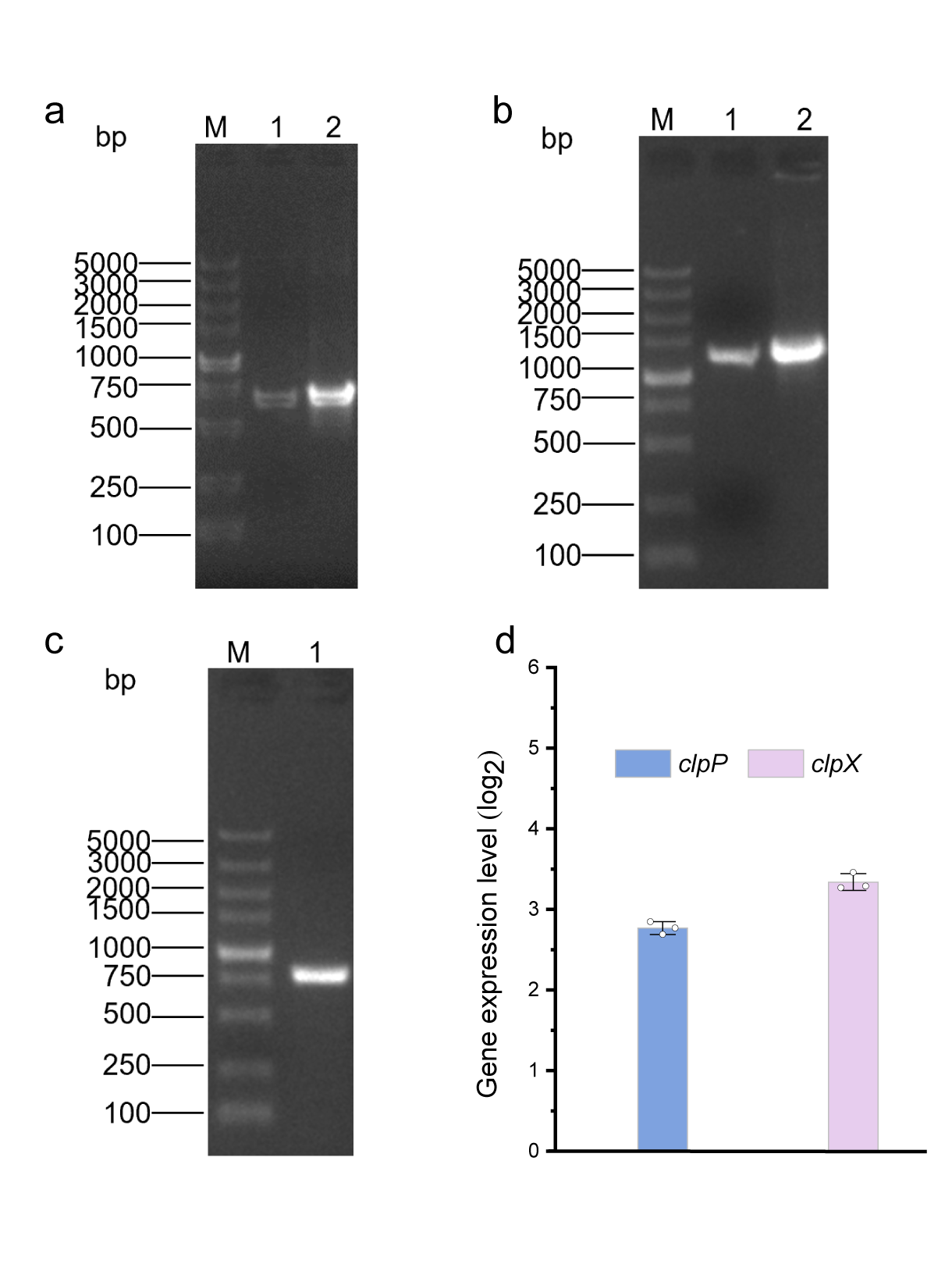


**Fig. S29 Construction of D184-*clpP*-*clpX* engineered strain. a-b** PCR verification of *clpP* and *clpX* in the pOJ260-P*kasO**-*clpP*-P*kasO**-*clpX* construct. **c** PCR verification of the *apr* gene in the *D184-clpP-clpX.***d** Transcriptional levels of *clpP* and *clpX.*


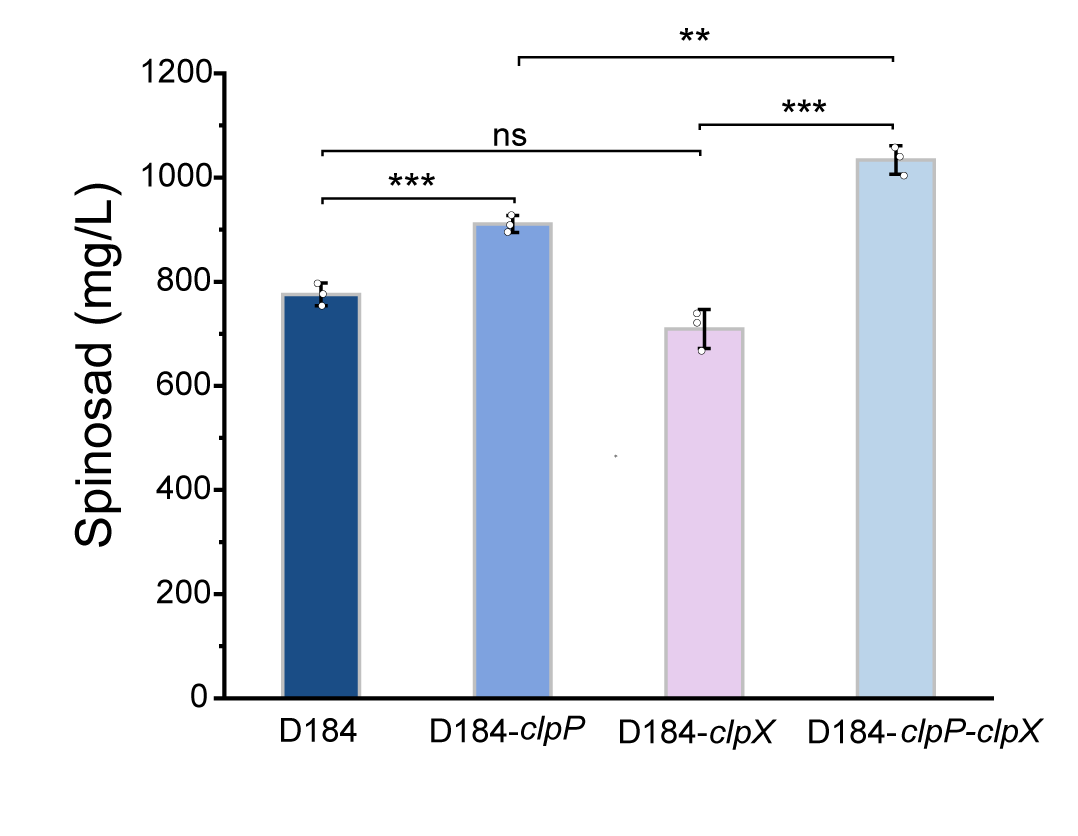


**Fig. S30 The titer of D184-*clpP*-*clpX* in shake flask cultures, showing the synthesis levels of spinosad.** Multiple comparison significance was tested to **P* < 0.05, ***P* < 0.01, ****P* < 0.001 by one-way ANOVA followed by Tukey’s post-hoc test.

**Table**

Table S1. Strains used in this study

| strains | Description | source |
| --- | --- | --- |
| *S. spinosa* (CCTCC M206084) | WT-strain | Lab store |
| D184 | ARTP mutation from S. spinosa | This study |
| D184-*dap* | The *dap* overexpression strain, derived from D184 | This study |
| D184-*alp* | The *alp* overexpression strain, derived from D184 | This study |
| D184-*pepP* | The *pepP* overexpression strain, derived from D184 | This study |
| D184-*htpX* | The *htpX* overexpression strain, derived from D184 | This study |
| D184-*metAP* | The *metAP* overexpression strain, derived from D184 | This study |
| D184-*clpP* | The *clpP* overexpression strain, derived from D184 | This study |
| D184-*pepN* | The *pepN* overexpression strain, derived from D184 | This study |
| D184-*ybbJ* | The *ybbJ* overexpression strain, derived from D184 | This study |
| D184- *clpA/clpB* | The *clpA/clpB* overexpression strain, derived from D184 | This study |
| D184-*dpp* | The *dpp* overexpression strain, derived from D184 | This study |
| D184-*lon* | The *lon* overexpression strain, derived from D184 | This study |
| D184-*yctJ* | The *yctJ* overexpression strain, derived from D184 | This study |
| D184-*vanY* | The *vanY* overexpression strain, derived from D184 | This study |
| D184-*mbp* | The *mbp* overexpression strain, derived from D184 | This study |
| D184-*ppl* | The *ppl* overexpression strain, derived from D184 | This study |
| D184-*clpX* | The *clpX* overexpression strain, derived from D184 | This study |
| D184-*abh* | The *abh* overexpression strain, derived from D184 | This study |
| D184-*serA* | The *serA* overexpression strain, derived from D184 | This study |
| D184-*zpeA* | The *zpeA* overexpression strain, derived from D184 | This study |
| D184-*ctr* | The *ctr* overexpression strain, derived from D184 | This study |
| D184-*clpC* | The *clpC* overexpression strain, derived from D184 | This study |
| D184-t*hrC* | The *thrC* overexpression strain, derived from D184 | This study |
| D184-*cysK* | The *cysK* overexpression strain, derived from D184 | This study |
| D184-*ivd* | The *ivd* overexpression strain, derived from D184 | This study |
| D184*-dCas9-only* | derived from D184, serves as a control strain carrying only the dCas9 gene without the CRISPRi targeting system. | This study |
| D184*-dCas9-dap* | The *dap* CRISPRi knockdown strain, derived from D184 | This study |
| D184*-dCas9-alp* | The *alp* CRISPRi knockdown strain, derived from D184 | This study |
| D184*-dCas9-pepP* | The *pepP* CRISPRi knockdown strain, derived from D184 | This study |
| D184*-dCas9-htpX* | The *htpX* CRISPRi knockdown strain, derived from D184 | This study |
| D184*-dCas9-metAP* | The *metAP* CRISPRi knockdown strain, derived from D184 | This study |
| D184*-dCas9-clpP* | The *clpP* CRISPRi knockdown strain, derived from D184 | This study |
| D184*-dCas9-pepN* | The *pepN* CRISPRi knockdown strain, derived from D184 | This study |
| D184*-dCas9-ybbJ* | The *ybbJ* CRISPRi knockdown strain, derived from D184 | This study |
| D184-*dap*-*alp* | The *dap* and *alp* co-overexpression strain, derived from D184 | This study |
| D184-*dap*-*pepP* | The *dap* and *pepP* co-overexpression strain, derived from D184 | This study |
| D184-*dap*-*htpX* | The *dap* and *htpX* co-overexpression strain, derived from D184 | This study |
| D184-*dap*-*metAP* | The *dap* and *metAP* co-overexpression strain, derived from D184 | This study |
| D184-*dap*-*clpP* | The *dap* and *clpP* co-overexpression strain, derived from D184 | This study |
| D184-*alp*-*pepP* | The *alp* and *pepP* co-overexpression strain, derived from D184 | This study |
| D184-*alp*-*htpX* | The *alp* and *htpX* co-overexpression strain, derived from D184 | This study |
| D184-*alp*-*metAP* | The *alp* and *metAP* co-overexpression strain, derived from D184 | This study |
| D184-*alp*-*clpP* | The *alp* and *clpP* co-overexpression strain, derived from D184 | This study |
| D184-*pepP-htpX* | The *pepP* and *htpX* co-overexpression strain, derived from D184 | This study |
| D184-*pepP-metAP* | The *pepP* and *metAP* co-overexpression strain, derived from D184 | This study |
| D184-*pepP-clpP* | The *pepP* and *clpP* co-overexpression strain, derived from D184 | This study |
| D184-*htpX-metAP* | The *htpX* and *metAP* co-overexpression strain, derived from D184 | This study |
| D184-*htpX-clpP* | The *htpX* and *clpP* co-overexpression strain, derived from D184 | This study |
| D184-*clpP-metAP* | The *clpP* and *metAP* co-overexpression strain, derived from D184 | This study |
| D184-*pepP-clpP-htpX* | The *pepP*,*clpP*, and *htpX* co-overexpression strain, derived from D184 | This study |
| D184-*pepP-clpP-metAP* | The *pepP*,*clpP*, and *metAP* co-overexpression strain, derived from D184 | This study |
| D184-*dap-clpP-htpX* | The *dap*,*clpP*, and *htpX* co-overexpression strain, derived from D184 | This study |
| ET12567/pUZ8002 | A donor strain used for conjugation. | Lab store |
| *E. coli* TOP10 | A *E. coli* host strain with replication and high transformation efficiency. | Lab store |

Table S2. Nucleotide sequences of primers

| Primers | Sequences (5'-3') | Source/reference |
| --- | --- | --- |
| F*_dap_* | ACT**TCTAGA**GTGCGGATCTTGCTCTCAGC | This study |
| R*_dap_* | GCG**GGATCC**TCAGCCGTAGCTGCCCTG | This study |
| F*_alp_* | ACT**TCTAGA**ATGAAGCGCAGACTCGCG | This study |
| R*_alp_* | GCG**GGATCC**TTACTGGGTGAGCAAGCTCAG | This study |
| F*_pepP_* | ACT**TCTAGA**AATGTCCGACACGCACTCC | This study |
| R*_pepP_* | GCG**GGATCC**TCAGACTTCGACGAGCTCC | This study |
| F*_htpX_* | ACT**TCTAGA**GTGCACAGTCACGTCAACGG | This study |
| R*_htpX_* | GCG**GGATCC**AGTCCGGTGCGCCGATATT | This study |
| F*_metAP_* | ACT**TCTAGA**AATGCCGGTTCGAGCTCC | This study |
| R*_metAP_* | GCG**GGATCC**TCAGGGCAGAGTCAGGATTTC | This study |
| F*_clpP_* | ACT**TCTAGA**ATGTGGCCCCGGTGGACAA | This study |
| R*_clpP_* | GCG**GGATCC**TCAGCCGGGCAGGTTCGC | This study |
| F*_ybbJ_* | ACT**TCTAGA**ATGAGAGACATGACACCCGCG | This study |
| R*_ybbJ_* | GCG**GGATCC**TCACAGCCCGGTCCCCA | This study |
| F*_pepN_* | ACT**TCTAGA**ATGGCACCACCGAACCTCAC | This study |
| R*_pepN_* | GCG**GGATCC**TCAGGAGCGATCGAACTCCC | This study |
| F*_serA_* | ACT**TCTAGA**ATGACCACCAACGAGGAGAG | This study |
| R*_serA_* | GCG**GGATCC**CTACGGCGCCTTGACCAC | This study |
| F*_clpC_* | ACT**TCTAGA**ATGGCGACGTTCTTCGGAC | This study |
| R*_clpC_* | GCG**GGATCC**GTTGCGGTGCCGGATCAG | This study |
| F*_dpp_* | ACT**TCTAGA**ATGCGTCGCGTTCGGTTG | This study |
| R*_dpp_* | GCG**GGATCC**TCACCCGATGGACCCGAC | This study |
| F*_abh_* | ACT**TCTAGA**ATGAAGCGTTCGATCGCACT | This study |
| R*_abh_* | GCG**GGATCC**CAGCACAGGTCGAAGGTCC | This study |
| F*_vanY_* | ACT**TCTAGA**ATGCGCTGCGGTGCGGTT | This study |
| R*_vanY_* | GCG**GGATCC**TCAGTCGGAATGCGTGCCG | This study |
| F*_zpeA_* | ACT**TCTAGA**ATGAGAGACATGACACCCGCG | This study |
| R*_zpeA_* | GCG**GGATCC**TCACAGCCCGGTCCCCA | This study |
| F*_YtcJ_* | ACT**TCTAGA**ATGCGCGAGGTGGCCTTC | This study |
| R*_YtcJ_* | GCG**GGATCC**CTAACGCTCGTGCACGACCA | This study |
| F*_clpX_* | ACT**TCTAGA**ATGGCACGTATCGGTGACG | This study |
| R*_clpX_* | GCG**GGATCC**GATATCTCAGGCGGACTTCTCGCG | This study |
| F*_mbp_* | ACT**TCTAGA**ATGACAGCAGGAGCGCAAAC | This study |
| R*_mbp_* | GCG**GGATCC**TCATGCGGACGGGCGGT | This study |
| F*_clpA/clpB_* | ACT**TCTAGA**ATGGCTGATTCAGTTGAGTTCT | This study |
| R*_clpA/clpB_* | GCG**GGATCC**TCATTTCTTCTTCTGGGCAA | This study |
| F*_lon_* | ACT**TCTAGA**AATGGACGTGCTGCCGCTC | This study |
| R*_lon_* | GCG**GGATCC**TCAGTTCACGCTGGTGTGCT | This study |
| F*_ctr_* | ACT**TCTAGA**ATGGTCGATGCCACACGTC | This study |
| R*_ctr_* | GCG**GGATCC**TCAGGAACCGATCTGCGC | This study |
| F*_ppl_* | ACT**TCTAGA**ATGCCGTCAGCGTCGTTTC | This study |
| R*_ppl_* | GCG**GGATCC**TTAGCGCGCCAGCGACTT | This study |
| F*_cysK_* | ACT**TCTAGA**ATGATCGACAGCACCCAAC | This study |
| R*_cysK_* | GCG**GGATCC**TCAGTCTGCCCGGAACTC | This study |
| F*_ivd_* | ACT**TCTAGA**AGTGGACCCGAAGTTCGGC | This study |
| R*_ivd_* | GCG**GGATCC**TCAGCCTTTGAGCAGCGAG | This study |
| F*_thrC_* | ACT**TCTAGA**AAGTGGAGGTGTGCGTTGTC | This study |
| R*_thrC_* | GCG**GGATCC**GGCGAGTTCAGTCGTAGATG | This study |
| F*_pepP_* | ACT**TCTAGA**AATGTCCGACACGCACTCC | This study |
| R*_pepP-clpP_* | TTGTCCACCGGGGCCACATTCAGACTTCGACGAGCTCC | This study |
| F*_pepP-clpP_* | GGAGCTCGTCGAAGTCTGAATGTGGCCCCGGTGGACAA | This study |
| R*_clpP_* | GCG**GAATTC**TCAGCCGGGCAGGTTCGC | This study |
| F*_dap_* | ACT**TCTAGA**GTGCGGATCTTGCTCTCAGC | This study |
| R*_dap-clpP_* | TTGTCCACCGGGGCCACATTCAGCCGTAGCTGCCCTG | This study |
| F*_dap-clpP_* | CAGGGCAGCTACGGCTGAATGTGGCCCCGGTGGACAA | This study |
| R*_clpP_* | GCG**GAATTC**TCAGCCGGGCAGGTTCGC | This study |
| F*_apr_* | TTATGTCATCAGCGGTGGAGTG | This study |
| R*_apr_* | TTAGCGGCATCGCATTCTT | This study |
| RT-qPCR |  |  |
| qF-*16S* | CGTCAGCTCGTGTCGTGAGA | This study |
| qR-*16S* | GTGAAGCCCTGGGCATAAGG | This study |
| qF-*dap* | CTTCCGCCGCCTGTTCAC | This study |
| qR-*dap* | CCGTCCACATCGGAATCGA | This study |
| qF-*alp* | GCTGCTCGGTCGGGTTCT | This study |
| qR-*alp* | CTGACGTTGCTGCCGTTGTA | This study |
| qF-*pepP* | GCCACGCCCTCCGTAACC | This study |
| qR-*pepP* | GCCATCGGTGCAGAACACG | This study |
| qF-*htpX* | CCAACTTCGGGCTGCTCTT | This study |
| qR-*htpX* | ATCAGGTGCGACTGCGACA | This study |
| qF-*metAP* | TTCGGCTACGGCGTGGTCC | This study |
| qR-*metAP* | ATTTCGTTGCCTTCTTCGGTGATT | This study |
| qF-*clpP* | CATTGTGGGAGAAGGCAGGG | This study |
| qR-*clpP* | GGCGAGTTGATGTAGAGCGAAA | This study |
| qF-*ybbJ* | CTCATCGCTGGGGTGCTACTC | This study |
| qR-*ybbJ* | AGGGCGTGCTTGCCGACT | This study |
| qF-*pepN* | CCACCACCAAGCCGATGTC | This study |
| qR-*pepN* | CCGAACGCCTTGTGGTAGAA | This study |
| qF-*serA* | GCATCCGCTGCGCCAAC | This study |
| qR-*serA* | GTGAACTCGCCGTCCTCCA | This study |
| qF-*clpC* | AGCACAGCACTGAGCTGATCGTTTTC | This study |
| qR-*clpC* | GCACCTGGACAGGCTGGAAGC | This study |
| qF-*dpp* | CGGTGGTGGACTGGCTGAACTC | This study |
| qR-*dpp* | GCCCCGTCGGAGCGGTAAT | This study |
| qF-*abh* | TGGTATCGCAACAACCTCAAC | This study |
| qR-*abh* | TGAATGTGATGTCGATGAACTCC | This study |
| qF-*vanY* | GCGGGCAAGGGCTACGAC | This study |
| qR-*vanY* | GCCACCACCGAGACCTGCTT | This study |
| qF-*zpeA* | AGCACCTCCTGTTCAAGGGCAC | This study |
| qR-*zpeA* | GTCGCAGAGCATGTCGATGGC | This study |
| qF-*ytcJ* | TACGACGACACGCTGGAGACCTAC | This study |
| qR-*YtcJ* | GACATCGGGGTCCACGAAGGA | This study |
| qF-*clpX* | CGAGACCGGGATCATCTACATCG | This study |
| qR-*clpX* | GAACTCCTGGTGCGGGTGCTT | This study |
| qF-*mbp* | TGGTCTCCTCCGGGCGTACC | This study |
| qR-*mbp* | CGTCGTCGTCCTCCCAGCAA | This study |
| qF-*clpA/clpB* | ATTCAGTTGAGTTCTCCGGTCAG | This study |
| qR-*clpA/clpB* | CCCATGCTCTTCCCGATTT | This study |
| qF-*lon* | CACCGCCCCGGAGAACG | This study |
| qR-*lon* | TTCCACGGTCAGCACGCAAT | This study |
| qF-*ctr* | CATCGCCGACCACCCGTTC | This study |
| qR-*ctr* | GCCCTGCGAGGCGTCCTG | This study |
| qF-*ppl* | CGGTGCCTCCGTGCTCTTCC | This study |
| qR-*ppl* | AACAGCCGCCCCGAGATGG | This study |
| qF-*spnB* | AGCATCGCTCAAGGAGAACG | This study |
| qR-*spnB* | CAGCCAGTTGCCACAGGTC | This study |
| qF-*spnC* | AGGGCTGTGGAAACTGGTC | This study |
| qR-*spnC* | CAGGAGATTTCCAGCAGCA | This study |
| qF-*spnE* | GAGGCACAGGCGATGGAC | This study |
| qR-*spnE* | TAACCCGAAGGTGTAAGCCA | This study |
| qF-*spnR* | TCCTCAACTCGGGGACTGC | This study |
| qR-*spnR* | TAGTGGAGCAGCAATACGGC | This study |
| qF-*spnQ* | CCTGGCACTGAGTTCGCTTA | This study |
| qR-*spnQ* | GTTGTAGGTGCCCAGTTCCA | This study |
| qF-*spnK* | AACTTCCTCCGAACCACCAC | This study |
| qR-*spnK* | TAGTGGGAGGCGAGCAAGTT | This study |
| qF-*spnF* | CCGTTGCTGAACTCGGTCG | This study |
| qR-*spnF* | GTCCGTTCGGCGACAAGGT | This study |
| qF-*pfk* | ACCTCGGACCTCGTGGAACTG | This study |
| qR-*pfk* | GGGCGGCGATGAGGAAGAT | This study |
| qF-*pgk* | CGGCGGCATGGCGTACA | This study |
| qR-*pgk* | GCGGAGTCGCGGAGGATC | This study |
| qF-*pyk* | CAGACTACGCTGGCATAACG | This study |
| qR-*pyk* | CACCATCTCGTAGACCTGCT | This study |
| qF-*gltA* | GCCTGCCTCTCCGACATCA | This study |
| qR-*gltA* | GAACTGTTTGAGGTCTTCGTGC | This study |
| qF-*glnA* | GCTGTTCGGTGACAACGGTTCC | This study |
| qR-*glnA* | AGCGGTTGCGCTGCGAGTAC | This study |
| qF-*idh* | CTTCATCAAGGACAAGCTGATCCA | This study |
| qR-*idh* | TTCGGGCTCCGCCACAT | This study |
| qF-*crr* | GTGAGCACCGAGGTTGGCA | This study |
| qR-*crr* | GACGAAAGCGTGCGGGTG | This study |
| qF-*ptsI* | GACAGCGGCGAGGTGGAGAC | This study |
| qR-*ptsI* | CGTTGGCGACGACCTTGACC | This study |
| qF-*fadA* | GAGCCGTTGACGTTGAGCC | This study |
| qR-*fadA* | CGCCGTTTGCGTGGTGA | This study |
| qF-*fadD* | GAGGTCGGCACCATCTACTTC | This study |
| qR-*fadD* | CATGGTTGCCCAGTTCTCG | This study |
| qF-*fadJ* | CGGCTGCTGACCAGGTTCATG | This study |
| qR-*fadJ* | GCTTCGCTCCGCTGACTCTTTG | This study |
| qF-*hmgL* | GGCTGCTGGAGATGGGTTG | This study |
| qR-*hmgL* | CCGTAGGTGTCGTGGAAGTG | This study |
| qF-*bcd* | GCTCACCCAGGCCACGTTC | This study |
| qR-*bcd* | GGACGAAGGTGCGGTTGACA | This study |
| qF-*PaaF* | GCGAGGTGGACGAGGTGC | This study |
| qR-*PaaF* | CTTCAGGGTGACCTTGAGGGA | This study |
| qF-*echA* | GGCCAAGGAAATGCTGTTC | This study |
| qF-*echA* | CGAGGCTGTTGTTGACGCT | This study |
| qF-*thrC* | CCATCGTCCGTTCGCTGT | This study |
| qR-*thrC* | GTCGATCTCGCCCGCTTC | This study |
| qF-*acd* | TGCTCAGCGGCGAGAAAAC | This study |
| qR-*acd* | ATGCCCGGCATCGGATAG | This study |
| qF-*ivd* | GCTTCCAGGGCGTCCAGTT | This study |
| qR-*ivd* | TTGGCGTCGCGCATCATC | This study |
| qF-*sigF* | GCAGATGACCAGCGTGAT | This study |
| qR-*sigF* | TAGAGGGTGACCACCACG | This study |
| qF-*whiB* | GAGGCGACGGACGAAGAAC | This study |
| qR-*whiB* | GCGTTCCCTTTCGGACAGT | This study |
| qF-*whiE* | CGAGGCGAAGGCGAAAG | This study |
| qR-*whiE* | CACTTCTCGCTCCGATTCC | This study |
| qF-*wblE* | AGTGGTCCTGCTGTGCTGC | This study |
| qR-*wblE* | CCGCTCGTCTTCGCTCAT | This study |
| qF-*whiA* | CCGACGGGCTGAGGTTTC | This study |
| qR-*whiA* | GTGCCCGAACAGCTCGTG | This study |
| qF-*ssgA* | GTTGGCTCCGCAGACACCG | This study |
| qR-*ssgA* | AGACCGTCGGCGAGCAGG | This study |
| sgRNA |  | This study |
| sgRNA-*dap*-F | AGTTCTAGAGGAATGCGTGAGCCGCTACGGTTTTAGAGCTAGAAATA | This study |
| sgRNA-*alp*-F | AGTTCTAGATCGTTCCCGGACAACGACTAGTTTTAGAGCTAGAAATA | This study |
| sgRNA-*pepP*-F | AGTTCTAGAACTGGAGACCCGGATGCTCGGTTTTAGAGCTAGAAATA | This study |
| sgRNA-*htpX*-F | AGTTCTAGAGCTCTACATCAGTCCGACGC GTTTTAGAGCTAGAAATA | This study |
| SgRNA-*metAP*-F | AGTTCTAGACGCCGTAGCCGAACCGCTTGGTTTTAGAGCTAGAAATA | This study |
| sgRNA-*clpP*-F | AGTTCTAGAACAACCGACCCTAACGAACGGTTTTAGAGCTAGAAATA | This study |
| sgRNA-*ybbJ*-F | AGTTCTAGAAAGCGACGGATGCACCACCCGTTTTAGAGCTAGAAATA | This study |
| sgRNA-*pepN*-F | AGTTCTAGAATCGGTATCTACGACGACGAGTTTTAGAGCTAGAAATA | This study |
| sgRNA-R | TACGAATTCGGGTGTACATCCAGTAATG | This study |

Table S3. Differential Amino Acid Metabolism in *S. spinosa* and D184

| Amino acid | Fold Change | P-value |
| --- | --- | --- |
| Val | 0.604578 | 0.005417 |
| Tyr | 0.597082 | 0.009832 |
| Trp | 0.608361 | 0.000838 |
| Thr | 0.670821 | 0.004195 |
| Ser | 0.612168 | 0.007864 |
| Phe | 0.64842 | 0.002745 |
| His | 0.698823 | 0.043857 |
| Gly | 0.582771 | 0.017448 |
| Pro | 0.792235 | 0.017529 |
| Asp | 0.696406 | 0.029759 |
| Arg | 0.502432 | 0.012137 |
| Ala | 0.715984 | 0.003177 |
| Asn | 0.609205 | 0.0222516 |
| Ile | 0.606677 | 0.0061417 |
| Gln | 0.459138 | 0.0078527 |
| Leu | 0.639493 | 0.0032054 |
| Lys | 0.649319 | 0.0069396 |
| Met | 0.639936 | 0.0080523 |
| Glu | 0.788947 | 0.0063427 |
| glycylphenylalanine | 0.290176 | 0.00626302 |
| Homoserine | 0.692075 | 0.05378131 |
| argininosuccinic acid | 0.529242 | 0.001773355 |
| O-Phospho-L-Serine | 2.353813 | 0.023602539 |
| L-Theanine | 0.48599 | 0.015124904 |
| L-Ornithine | 0.706127 | 0.014583179 |
| Succinic Acid | 0.345798 | 0.00301548 |
| Sarcosine | 0.488016 | 0.005765218 |
| S-Sulfo-L-Cysteine | 1.50629 | 0.01335268 |
| L-Citrulline | 0.465547 | 0.001351925 |

Note: The table presents the upregulated and downregulated amino acid metabolism data from the metabolomics analysis of *S. spinosa* and D184. Statistical significance was determined using a two-tailed Student’s *t*-test (P < 0.05). FC values ≥ 1.5 are considered upregulated, while FC values≤ 0.6667 are considered downregulated.

Table S4 Differential Glucose and Energy Metabolism in S. spinosa and D184

|  | Fold Change | P-value |
| --- | --- | --- |
| DL-Glyceric-Acid | 5.006856 | 0.0018 |
| Ureidopropionate | 0.378798 | 0.0034 |
| Gluconate | 1.229366 | 0.0446 |
| Cysteic-acid | 5.52045 | 0.0028 |
| D(+)-Glucose | 0.438318 | 0.0020 |
| Succinyl-CoA | 1.454662 | 0.0549 |
| Acetyl-CoA | 1.736873 | 0.0699 |
| Guanosine | 6.651916 | 0.0308 |
| ATP | 9.889201 | 0.0055 |
| UMP | 1.508259 | 0.0682 |
| AMP | 1.398835 | 0.0451 |
| Uracil | 0.418599 | 0.0243 |
| IMP | 1.548942 | 0.0134 |
| ADP | 3.556534 | 0.0029 |
| Inosine | 3.090116 | 0.0267 |
| NicotinaMide-adenine-dinucleotide(NAD) | 0.597723 | 0.0073 |
| Malic-acid | 3.883714 | 0.0005 |
| Oxaloacetate | 0.676523 | 0.0387 |
| cis-Aconitic-acid | 0.140927 | 0.0054 |
| Argininosuccinic-acid | 2.242267 | 0.0418 |
| Pyruvic-acid | 0.269676 | 0.0032 |
| Succinic-Acid | 2.084598 | 0.0284 |
| 3-phenyllactic-acid | 0.29814 | 0.0015 |
| D-Erythrose-4-phosphate | 0.550479 | 0.0397 |
| D-Fructose-6-phosphate | 0.593755 | 0.0198 |
| D-Ribulose-5-phosphate | 0.739427 | 0.0254 |
| D-Mannose-6-phosphate | 0.692003 | 0.0066 |
| D-Glucose-6-phosphate | 0.613668 | 0.0041 |
| D-Glucose-1-phosphate | 0.59568 | 0.0320 |
| Dihydroxyacetone-phosphate | 0.263234 | 0.0012 |
| Glycerol-3-phosphate | 1.756119 | 0.0305 |
| Phosphoenolpyruvic-acid | 2.497873 | 0.0007 |
| 3-phosphoglycerate | 2.102288 | 0.0117 |
| 6-Phosphogluconic-acid | 2.678901 | 0.0020 |
| Glyceraldehyde-3-phosphate | 0.243472 | 0.0009 |

Note: The table presents the upregulated and downregulated glucose and energy metabolism data from the metabolomics analysis of *S. spinosa* and D184. Statistical significance was determined using a two-tailed Student’s *t*-test (P < 0.05). FC values ≥ 1.5 are considered upregulated, while FC values≤ 0.6667 are considered downregulated.

Table S5. Functional Profiling of 21 Protease Candidates

| functional modules | Gene | ORF names | Peptidase | Function |
| --- | --- | --- | --- | --- |
| ATP-dependent proteostasis | ***clpP*** | A8926_2493 | ClpP-dependent Clp protease proteolytic subunit | A key component of the Clp protease complex, involved in protein degradation, particularly for damaged or misfolded proteins ^[2]^. |
|  | *clpX* | A8926_2495 | ATP-dependent Clp protease ATP-binding subunit ClpX | Part of the ClpXP protease complex, it recognizes and unfolds substrates for degradation by the ClpP subunit, powered by ATP hydrolysis. |
|  | *clpC* | A8926_3530 | ATP-dependent Clp protease ATP-binding subunit ClpC | Similar to ClpX, it helps in the unfolding of substrates for degradation, playing a role in protein homeostasis and stress response. |
|  | *clpA/ClpB* | A8926_2731 | Clp protease N-terminal domain-containing protein | ClpA and ClpB are ATPases that work in conjunction with ClpP, involved in protein unfolding and degradation, particularly under stress conditions. |
|  | *lon* | A8926_7555 | Lon N-terminal domain-containing protein | A protease involved in the degradation of damaged proteins, regulating the turnover of cellular components and stress response pathways. |
| Amino acid supply (exopeptidase cascade) | ***metAP*** | A8926_7726 | Methionine aminopeptidase | An enzyme responsible for the N-terminal methionine removal of nascent polypeptides, enhancing protein maturation and reducing folding burden ^[3]^. |
|  | ***pepP*** | A8926_3905 | Xaa-Pro aminopeptidase | An enzyme that removes amino acids from the N-terminus of peptides, particularly those with proline as the second residue, is involved in peptide processing ^[4]^. |
|  | ***pepN*** | A8926_2467 | Aminopeptidase N | Responsible for the removal of the N-terminal amino acid from peptides, playing a key role in protein degradation and the regulation of peptide pools ^[5, 6]^. |
|  | *dpp* | A8926_2916 | Xaa-Pro dipeptidyl-peptidase | A dipeptidase enzyme that cleaves dipeptides with proline at the second position, involved in peptide turnover and metabolism. |
|  | *vanY* | A8926_4838 | Zinc carboxypeptidase | A carboxypeptidase that removes C-terminal amino acids from peptides, potentially involved in antibiotic resistance mechanisms. |
|  | ***dap*** | A8926_7086 | D-aminopeptidase | A specific enzyme that hydrolyzes D-amino acids from peptides, playing a role in peptide metabolism and maintaining cellular homeostasis ^[7]^. |
| Amino acid supply (processing endoproteolysis) | ***alp*** | A8926_1520 | alpha-lytic protease | A protease that cleaves peptide bonds, often involved in the breakdown of proteins under specific conditions, such as stress or nutrient limitation ^[8]^. |
|  | *ctr* | A8926_2313 | Chymotrypsin | A serine protease that cleaves peptide bonds at the carboxyl side of aromatic amino acids, important for protein digestion and processing. |
|  | *serA* | A8926_5341 | S8 family serine peptidase | A member of the S8 family of serine proteases, involved in various proteolytic activities, including protein maturation and degradation. |
|  | *ppl* | A8926_3445 | prolyl oligopeptidase family serine peptidase | A protease that cleaves peptide bonds involving proline, involved in the processing of peptides during protein degradation. |
|  | *zpeA* | A8926_7643 | M16 family peptidase-Putative Zn-dependent peptidase | A zinc-dependent protease from the M16 family, likely involved in the breakdown of peptides in the cell. |
|  | *ytcJ* | A8926_5689 | Amidohydrolase 3 domain-containing protein | A hydrolytic enzyme that may break down amides, contributing to various metabolic processes, including peptide and protein degradation. |
|  | *abh* | A8926_5556 | Alpha/beta hydrolase | An enzyme with a broad range of hydrolytic activities, typically involved in the breakdown of ester bonds in lipids, proteins, or other macromolecules. |
| membrane-integrated proteostasis | ***htpX*** | A8926_7053 | Zinc metalloprotease HtpX | A metalloprotease that participates in protein degradation, likely involved in maintaining cellular protein homeostasis under stress ^[9]^. |
|  | ***ybbJ*** | A8926_5157 | Membrane protein (regulator of membrane protease activity) | Membrane protein (regulator of membrane protease activity): Likely involved in the regulation of membrane-bound proteases, influencing protein processing or turnover in the membrane environment ^[10]^. |
|  | *mbp* | A8926_6504 | membrane dipeptidase | An enzyme located in the membrane, likely involved in the breakdown of dipeptides within the membrane environment. |

Note: Gene-level information can be retrieved via the National Center for Biotechnology Information (NCBI) (<https://www.ncbi.nlm.nih.gov/#!/edu/home/principal/inicio>) by searching the **ORF names** provided

Table S6. Functional classification of 21 protease candidates

| functional modules | Gene | Peptidase | ID (uniprot) | MEROPS family | Mode(endo/exo) | Localization |
| --- | --- | --- | --- | --- | --- | --- |
| ATP-dependent proteostasis | ***clpP*** | ClpP-dependent Clp protease proteolytic subunit | A0A2N3XW48 | S14(ClpP endopeptidase; Ser endopeptidase) | Endopeptidase | Cytosol |
|  | *clpX* | ATP-dependent Clp protease ATP-binding subunit ClpX | A0A2N3XW07 | non-peptidase; AAA^+^ unfoldase ATPase |  | Cytosol |
|  | *clpC* | ATP-dependent Clp protease ATP-binding subunit ClpC | A0A2N3XYP3 | non-peptidase; AAA^+^ unfoldase ATPase |  | Cytosol |
|  | *clpA/ClpB* | Clp protease N-terminal domain-containing protein | A0A2N3XWJ7 | non-peptidase; AAA^+^ unfoldase ATPase |  | Cytosol |
|  | *Lon* | Lon N-terminal domain-containing protein | A0A2N3Y8Z8 | S16 (Lon protease; ATP-dependent Ser endopeptidase) | Endopeptidase | Cytosol |
| Amino acid supply (exopeptidase cascade) | ***metAP*** | Methionine aminopeptidase | A0A2N3Y9E7 | M24 (methionine aminopeptidase; often M24A/B) | Exopeptidase (N-term) | Cytosol |
|  | ***pepP*** | Xaa-Pro aminopeptidase | A0A2N3XZN3 | M24B (aminopeptidase P; metallopeptidase) | Exopeptidase (N-term) | Cytosol |
|  | ***pepN*** | Aminopeptidase N | A0A2N3XVW5 | M1 (aminopeptidase N; gluzincin metallopeptidase) | Exopeptidase (N-term) | Cytosol |
|  | *dpp* | Xaa-Pro dipeptidyl-peptidase | A0A2N3XX17 | S15 (Xaa-Pro dipeptidyl-peptidase; Ser exopeptidase) | Exopeptidase (dipeptidyl, N-term) | Cytosol |
|  | *vanY* | Zinc carboxypeptidase | A0A2N3Y207 | M15 (DD-carboxypeptidase; Zn carboxypeptidase) | Exopeptidase (C-term) | Cytosol |
|  | ***dap*** | D-aminopeptidase | A0A2N3Y7M8 | M20 (D-aminopeptidase; species-dependent) | Exopeptidase (N-term) | Cytosol |
| Amino acid supply (processing endoproteolysis) | ***alp*** | alpha-lytic protease | A0A2N3XTP0 | S1A (chymotrypsin family; Ser endopeptidase) | Endopeptidase | Cytosol |
|  | *ctr* | Chymotrypsin | A0A2N3XVK7 | S1A (chymotrypsin family; Ser endopeptidase) | Endopeptidase | Cytosol |
|  | *serA* | S8 family serine peptidase | A0A2N3Y393 | S8 (subtilisin-like serine endopeptidase) | Endopeptidase | Cytosol |
|  | *ppl* | prolyl oligopeptidase family serine peptidase | A0A2N3XYD9 | S9 (prolyl oligopeptidase family; Ser endopeptidase), | Endopeptidase (Pro-specific) | Cytosol |
|  | *zpeA* | M16 family peptidase-Putative Zn-dependent peptidase | A0A2N3Y995 | M16 (pitrilysin family; metallopeptidase) | Endo-/Exopeptidase (context-dependent) | Cytosol |
|  | *ytcJ* | Amidohydrolase 3 domain-containing protein | A0A2N3Y459 | amidohydrolase 3 domain; peptidase status TBD |  | Cytosol |
|  | *abh* | Alpha/beta hydrolase | A0A2N3Y3S0 | alpha/beta hydrolase; putative peptidase, family TBD | Endopeptidase (putative) | Cytosol |
| membrane-integrated proteostasis | ***htpX*** | Zinc metalloprotease HtpX | A0A2N3Y7J2 | M48 (membrane Zn metalloprotease) | Endopeptidase | Membrane |
|  | ***ybbJ*** | Membrane protein (regulator of membrane protease activity) | A0A2N3Y2T4 | regulatory membrane protein;non-peptidase |  | Membrane |
|  | *mbp* | membrane dipeptidase | A0A2N3Y6B7 | M19 (membrane dipeptidase;metallopeptidase) | Exopeptidase (dipeptidyl) | Membrane |

Note: Genes that were overexpressed to improve spinosad titer are highlighted in bold. Protein-level information can be retrieved in **UniProt** (<https://www.uniprot.org/>) by searching the **protein IDs** listed

Table S7. Overexpression strain analysis of insecticidal activity

| Strains | LT_50_ (h) | 95% confidence interval |
| --- | --- | --- |
| WT-strain *S. spinosa* | 114.3095 | 102.947-121.053 |
| D184 | 67.7245 | 54.319-75.681 |
| D184-*dap* | 41.6205 | 31.187-47.813 |
| D184-*alp* | 44.284 | 33.046-50.954 |
| D184-*pepP* | 44.812 | 33.437-51.563 |
| D184-*htpX* | 47.9755 | 35.796-55.204 |
| D184-*metAP* | 49.962 | 37.848-57.152 |
| D184-*clpP* | 51.066 | 38.441-58.559 |
| D184-*pepN* | 54.6705 | 41.532-62.468 |
| D184-*ybbJ* | 56.1535 | 43.099-63.901 |

Table S8. List of proteins with abbreviations

| Protein (abbreviation) | Full protein name | Gene |
| --- | --- | --- |
| AroB | 3-dehydroquinate synthase | *aroB* |
| AroC | chorismate synthase | *aroC* |
| PheA1 | chorismate mutase | *pheA1* |
| TrpB | tryptophan synthase beta chain | *trpB* |
| HisC | histidinol-phosphate aminotransferase | *hisC* |
| KynU | kynureninase | *kynU* |
| DLD | dihydrolipoyl dehydrogenase | *DLD* |
| PaaF | enoyl-CoA hydratase | *paaF* |
| Hpd | 4-hydroxyphenylpyruvate dioxygenase | *hpd* |
| Hgd | homogentisate 1,2-dioxygenase | *hgd* |
| PaaK | phenylacetate-CoA ligase | *paaK* |
| IlvC | ketol-acid reductoisomerase | *ilvC* |
| IlvD | dihydroxy-acid dehydratase | *ilvD* |
| IlvE | branched-chain amino acid aminotransferase | *ilvE* |
| LeuB | 3-isopropylmalate dehydrogenase | *leuB* |
| LeuC | 3-isopropylmalate dehydrogenase | *leuC* |
| LeuD | 3-isopropylmalate/(R)-2-methylmalate dehydratase | *leuD* |
| BkdA1 | 2-oxoisovalerate dehydrogenase E1 component subunit alpha | *bkdA1* |
| BkdB | 2-oxoisovalerate dehydrogenase E2 component (dihydrolipoyl transacylase) | *bkdB* |
| AcdH | short-chain 2-methylacyl-CoA dehydrogenase | *acdH* |
| HmgL | hydroxymethylglutaryl-CoA lyase | *hmgL* |
| FadA | acetyl-CoA acyltransferase | *fadA* |
| SerB | phosphoserine phosphatase | *serB* |
| GlyA | glycine hydroxymethyltransferase | *glyA* |
| ItaE | L-threonine aldolase | *itaE* |
| Pgi | glucose-6-phosphate isomerase | *pgi* |
| Pfk | 6-phosphofructokinase | *pfk* |
| GapA | glyceraldehyde 3-phosphate dehydrogenase (phosphorylating) | *gapA* |
| Pgk | phosphoglycerate kinase | *pgk* |
| Zwf | glucose-6-phosphate 1-dehydrogenase | *zwf* |
| Gnd | 6-phosphogluconate dehydrogenase | *gnd* |
| GltA | citrate synthase | *gltA* |
| Icd | isocitrate dehydrogenase | *icd* |
| FumA | Fumarate hydratase | *fumA* |
| Mdh | malate dehydrogenase | *mdh* |
| FadJ | 3-hydroxyacyl-CoA dehydrogenase / enoyl-CoA hydratase | *fadJ* |
| FabG | 3-oxoacyl-[acyl-carrier protein] reductase | *fabG* |
| ALDH | aldehyde dehydrogenase (NAD+) | *ALDH* |
| CysK | cysteine synthase | *cysK* |
| ThrC | threonine synthase | *thrC* |
| Ald | alanine dehydrogenase | *ald* |
| MatB | malonyl-CoA/methylmalonyl-CoA synthetase | *matB* |
| Fba | fructose-bisphosphate aldolase | *fba* |
| Pyk | pyruvate kinase | *pyk* |
| Aco | aconitate hydratase | *aco* |
| FabF | 3-oxoacyl-[acyl-carrier-protein] synthase II | *fabF* |

**References**

[1] H. Yan, X. Lu, D. Sun, S. Zhuang, Q. Chen, Z. Chen, J. Li, Y. Wen, *Molecular Microbiology* **2020**, *113* (1), 123, <https://doi.org/10.1111/mmi.14405>

[2] A. O. Olivares, T. A. Baker, R. T. Sauer, *Nature Reviews Microbiology* **2016**, *14* (1), 33, <https://doi.org/10.1038/nrmicro.2015.4>

[3] I. J. Berry, V. M. Jarocki, J. L. Tacchi, B. B. A. Raymond, M. Widjaja, M. P. Padula, S. P. Djordjevic, *Scientific Reports* **2017**, *7*, <https://doi.org/10.1038/s41598-017-11296-9>

[4] M. Matsushita-Morita, S. Tada, S. Suzuki, R. Hattori, K.-I. Kusumoto, *Journal of Bioscience and Bioengineering* **2017**, *124* (5), 534, <https://doi.org/10.1016/j.jbiosc.2017.06.007>

[5] A. Addlagatta, L. Gay, B. W. Matthews, *Proceedings of the National Academy of Sciences of the United States of America* **2006**, *103* (36), 13339, <https://doi.org/10.1073/pnas.0606167103>

[6] G. Yu, L. Li, X. Liu, G. Liu, Z. Deng, M. T. Zabriskie, M. Jiang, X. He, *Scientific Reports* **2015**, *5*, <https://doi.org/10.1038/srep17641>

[7] S. Rigali, F. Titgemeyer, S. Barends, S. Mulder, A. W. Thomae, D. A. Hopwood, G. P. van Wezel, *Embo Reports* **2008**, *9* (7), 670, <https://doi.org/10.1038/embor.2008.83>

[8] L. P. Tripathi, R. Sowdhamini, *Bmc Genomics* **2008**, *9*, <https://doi.org/10.1186/1471-2164-9-549>

[9] H. H. Huang, Y. T. Lin, P. Y. Chen, L. H. Li, H. C. Ning, T. C. Yang, *Antimicrobial Agents and Chemotherapy* **2018**, *62* (8), <https://doi.org/10.1128/AAC.00554-18;e00554-18>

[10] W. Liu, M. Schoonen, T. Wang, S. McSweeney, Q. Liu, *Communications Biology* **2022**, *5* (1), <https://doi.org/10.1038/s42003-022-03213-2>
